# Photolysis of ortho-Nitrobenzyl Esters: Kinetics and Substituent Effects

**DOI:** 10.1021/acsomega.5c08422

**Published:** 2025-11-20

**Authors:** Anthony L. Fink, Alexander G. Groß, Florian Puch, Robert Geitner

**Affiliations:** † Group of Physical Chemistry/Catalysis, Department of Natural Sciences and Mathematics, 26559 Technische Universität Ilmenau, Weimarer Str. 32, 98693 Ilmenau, Germany; ‡ Plastics Technology Group, Department of Mechanical Engineering, Thuringian Center of Innovation in Mobility, 26559 Technische Universität Ilmenau, Gustav-Kirchhoff Str. 5, 98693 Ilmenau, Germany; § Thüringisches Institut für Textil- und Kunststoff-Forschung e.V., Breitscheidstr. 97, 07407 Rudolstadt, Germany

## Abstract

ortho-Nitrobenzyl (oNB) esters are widely employed as photolabile protecting groups and linkers in diverse applications ranging from materials science to chemical biology. While their photochemical decomposition mechanism has been well studied, a comprehensive understanding of the structure–property relationships governing their photolysis kinetics remains lacking. In this study, the synthesis and kinetic analysis of 11 novel oNB esters with varying ester and aromatic substituents are reported. Using time-resolved NMR spectroscopy, we determined first-order rate constants for the photochemical cleavage of each compound under UV irradiation. A clear correlation between the reaction rate and the acidity (p*K*_a_) of the corresponding acid anion, with more acidic leaving groups yielding faster decomposition, was found. In contrast, the nature of the substituents on the aromatic ring shows no strong correlation with Hammett parameters or steric hindrance, quantified by Sterimol parameters. A multilinear model incorporating both steric and electronic parameters confirms this observation. Our findings highlight the critical role of the ester moiety in tuning photolysis rates while suggesting that aromatic substitution can be strategically employed for functionalization (e.g., polymer cross-linking) without substantially affecting reactivity. These insights lay the foundation for the rational design of photoresponsive oNB-based linkers for advanced materials and chemical applications.

## Graphical abstract

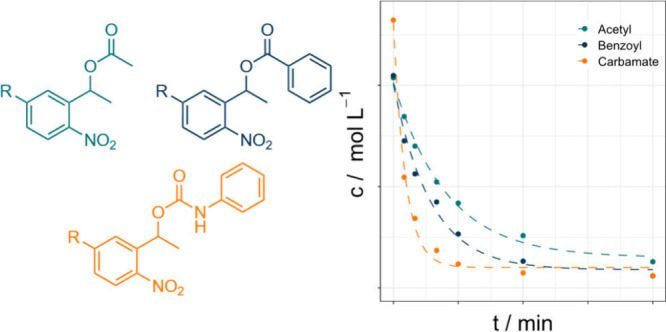

## Introduction

ortho-Nitrobenzyl (oNB) alcohols and their derivatives are well known for their application as photolinkers. Upon irradiation with UV or blue light, the nitro group gets photoactivated, leading to a triplet state (**I**).^1^ The unpaired electrons undergo a hydrogen transfer from the neighboring benzyl position forming a C-centered radical and leaving behind an O-centered radical at the former nitro group (**II**).^2^ These radicals recombine to form a five-membered ring,^3^ an isoxazole (**III**), which finally decomposes into a nitroso group and a carbonyl group at the former benzyl position (**IV**). This ring-opening reaction also eliminates the former hydroxyl group at the benzyl position (**V**, see Scheme 1). This elimination is not limited to alcohols but also extends to their derivatives like esters, ethers, and amides.^4–6^ This opens the possibility to use oNB derivatives as photosensitive linkers^7,8^ or protecting groups.^9^ For example, Tomaya et al. used oNB alcohols as photocleavable protecting groups during RNA synthesis.^10^ Szychowski et al. used oNB esters to attach biotin probes to proteins and subsequently trigger a release of the biotin fragment.^11^ oNB esters were also used in material science where they created releasable side groups in copolymers^12^ or dynamic polymer networks.^13,14^

**Scheme 1:**
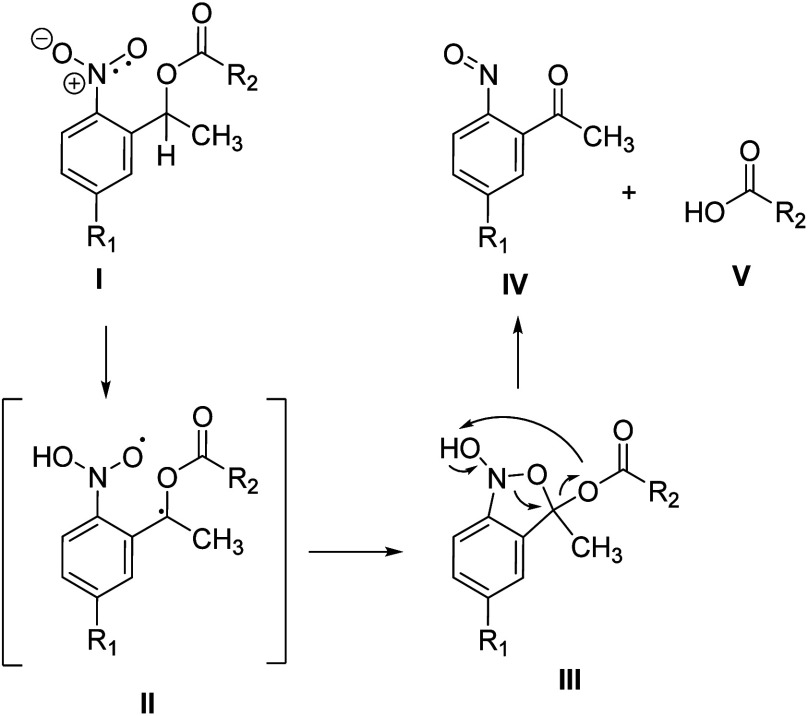
Photochemical Decomposition Mechanism for oNB Esters via a Triplet State and an Intramolecular H-Abstraction

The broad application of oNB derivatives also sparked interest in their fundamental photophysical properties including their absorption behavior, quantum yields,^15^ reaction kinetics,^16^ isotope effects,^17^ and reaction mechanism.^2,18–20^ Many studies found first-order reaction kinetics when it comes to the photochemical decomposition of oNB derivatives.^16^ Most interestingly, Blanc and Bochet found by isotope studies that the internal H-abstraction by the radical is the rate-limiting step.^17^ This effect is diminished when shorter wavelengths are used for the initial excitation, which is assigned to the involvement of higher excited states.

We recently used oNB esters to produce photolabile polyamide fibers which can be useful for the recycling of fiber-composite materials.^21^ While the oNB esters are a well-studied class of molecules, it was surprising to find that very little is known about the structure–property relationship regarding photochemical decomposition. The data of Blanc and Bochet^17^ indicates that the substituents have an effect on the reaction kinetics, but they did not further elaborate on the substituent effect in general. Holmes showed that increasing the electron density in the aromatic ring via the incorporation of two alkoxy groups at the benzene ring increased the rate of cleavage.^22^ Reichmanis et al. investigated the photosensitivity of six different 2-nitrobenzyl ester deep UV resists with varying methoxy, nitro and cyano substitution patterns at the phenyl ring.^23^ Besides these reports, to the best of our ability, we could not find any systematic studies on the influence of substituents on the photochemical decomposition of oNB esters.

Consequently, in this study 11 novel oNB esters including their syntheses, their properties, and their light-driven decomposition behavior are presented. The photolysis behavior correlates with the *pK*_*a*_ values of the released acid anions as well as weakly with the substituents on the phenyl ring. We intend to use our molecular understanding in the future to synthesize rationally designed oNB esters for cross-linking tasks in polyamides.

## Results and Discussion

To test the influence of the leaving group and the substituents at the phenyl ring, 11 new oNB esters were synthesized (Scheme 2). Details about the synthesis protocols can be found in the SI. The synthesis of acid chloride **2** occurred without any issues; however, it was found that the ratio of acid to thionyl chloride can be increased without any significant changes to the yield of the reaction in comparison to the literature protocol.^24^ The synthesis of **3** ^25^ produced varying yields which can be attributed to the loss during workup after the decarboxylation step. The best results were achieved when the suspension that was produced in the alkalization step was first vacuum filtrated before the extraction. The resulting residue was then extracted with diethyl ether before being dried. In a second extraction, additional product can be recovered from the dried residue. The radical bromination of **3** ^24^ is the step with the lowest yield and has the most potential for improvement. The reason is the formation of a side product due to secondary bromination at the benzyl position. To prevent the formation of the dibrominated side product during the brominating step, only 1.1 equiv of *N*-bromosuccinimide was used. The reduction of brominated ketone **4** and the subsequent substitution of the bromide in **5** ^11^ proceeded as expected. However, the purification of the resulting product **6e** by flash chromatography proved challenging to scale up, as the solubility in the eluent was poor. Increasing the polarity of the eluent improved the solubility at the cost of separation performance. The purification by flash chromatography is the limiting step in the scale up of this synthesis. The direct esterification of the hydroxyl group in alcohols **6** proved to be challenging, as the hydroxyl group has low reactivity. A reaction with acid chlorides did not occur under mild conditions. Instead, *n*-butyllithium had to be used to deprotonate the hydroxyl group. While the reaction did occur under these conditions, the product could not be purified. Instead, the esterification using the corresponding acid anhydrides and catalytic amounts of 4-dimethyl­aminopyridine proved superior to direct esterification. Compared to the esters, carbamate **10f** formed even under mild conditions using triethylamine.

**Scheme 2:**
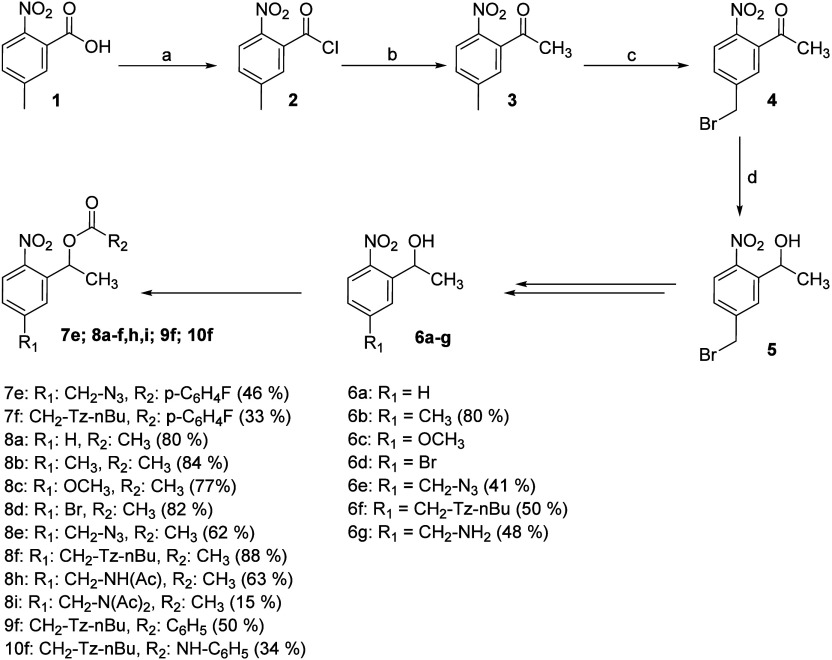
Synthesis of oNB Derivatives^a^

a Reaction conditions and yields: (a) SOCl_2_, DMF (cat.), 90%. (b) 1. Mg­(OEt)_2_ (1.1 equiv), diethylmalonate (1.1 equiv), THF; 2. AcOH, H_2_SO_4_, 78%. (c) NBS (1.1 equiv), benzoyl peroxide (5 mol %), CCl_4_, 40%. (d) NaBH_4_, MeOH:1,4-dioxane (10:7), 88–96%. The reactions conditions for the last two steps vary depending on the substituents. Details can be found in the Supporting Information.

After the successful synthesis of different oNB esters, time-dependent ^1^H NMR was used to follow the photochemical decomposition of these molecules. Figure 1a depicts a typical series of NMR spectra. As can be seen, the signals can be assigned to the starting oNB ester and the resulting acid anion after decomposition. In Figure 1a, the signal at 1.99 ppm can be assigned to the methyl group of the acetyl moiety in **8f** while the signal at 1.92 ppm can be assigned to the methyl group in acetate. After the ^1^H NMR spectra are normalized, the signal integrals can be used to derive the concentration of the oNB ester and the acid anion and track the concentrations over increasing irradiation times (Figure 1b and Figures S1–S15; figures numbered “S” can be seen in the Supporting Information). As done in other reports,^16^ a first-order kinetic fit was applied to analyze the photochemical decomposition behavior. Consequently, an apparent kinetic rate constant *k*, which consists of the constant photon flux density and the molecule specific decomposition rate, and a half-life time τ can be extracted for each oNB ester describing its photolysis (Table 1).

**Figure 1:**
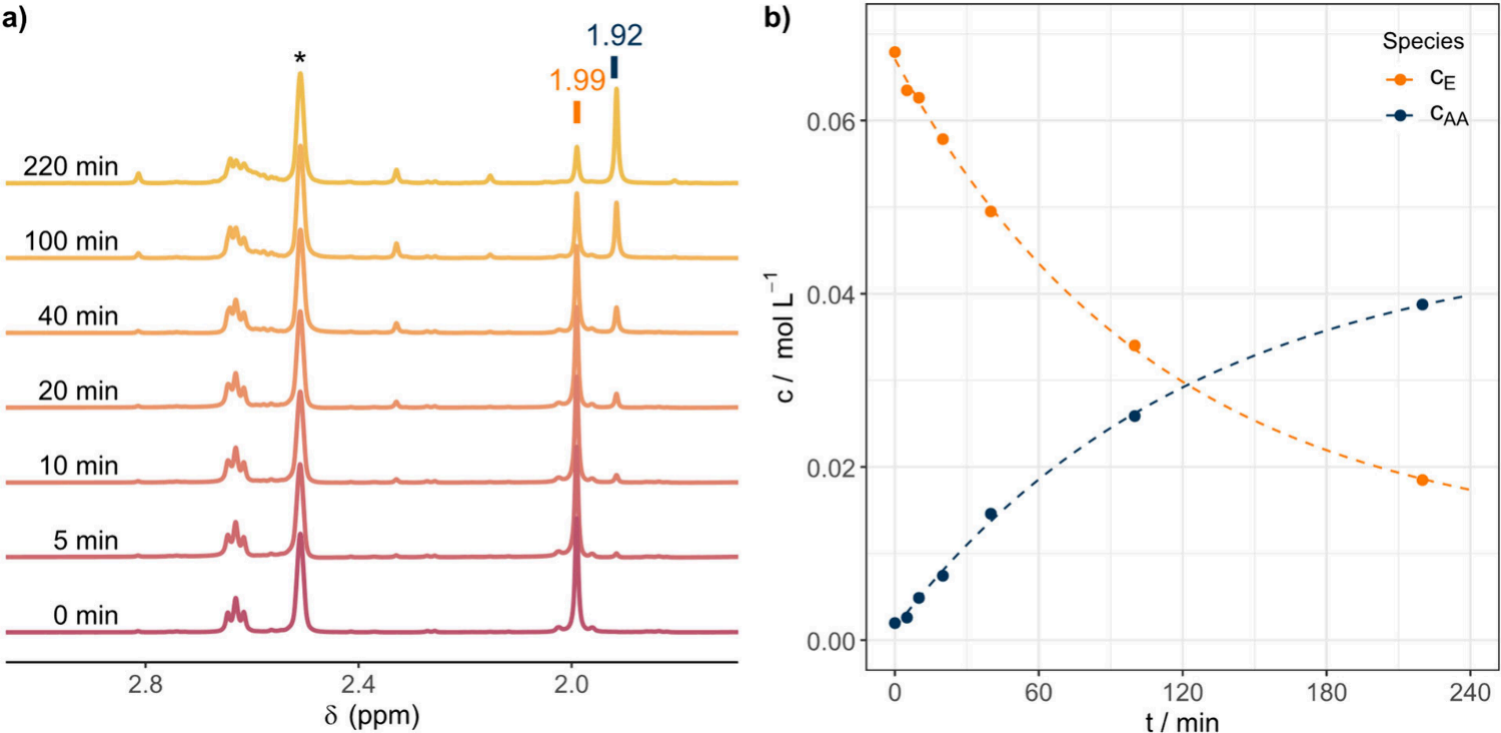
Kinetic evaluation of the photochemical decomposition of oNB esters. a) Vector-normalized ^1^H NMR spectra after different irradiation times for **8f**. The CH_3_ signal at 1.99 ppm belonging to the oNB educt (*c*_*E*_) is decreasing, while the signal of the acetate ion (*c*_*AA*_) increases at 1.92 ppm. b) Depiction of the NMR-deduced concentrations over irradiation time as well as first-order kinetic fits for **8f**.

**Table 1:** Overview of the Conducted Experiments^a^

Tag	R_1_	R_2_	c_0_ [mmol L^–1^]	k [10^–5^ s^–1^]	τ [min]
**7e**	–CH_2_–N_3_	–p-C_6_H_4_F	25	41.4	27.9
**7e**	–CH_2_–N_3_	–p-C_6_H_4_F	50	27.1	42.7
**7e**	–CH_2_–N_3_	–p-C_6_H_4_F	95	14.9	77.7
**7e**	–CH_2_–N_3_	–p-C_6_H_4_F	50	26.1^a^	44.2^a^
**8a**	–H	–CH_3_	29	50.8	22.7
**8b**	–CH_3_	–CH_3_	27	72.7	15.9
**8c**	–OCH_3_	–CH_3_	27	34.2	33.8
**8d**	–Br	–CH_3_	27	47.8	24.1
**8e**	–CH_2_–N_3_	–CH_3_	25	58.1	19.9
**8h**	–CH_2_–NH(Ac)	–CH_3_	26	95.5	12.1
**8i**	–CH_2_–N(Ac)_2_	–CH_3_	25	50.8	22.8
**7f**	–CH_2_–Tz–nBu	–p-C_6_H_4_F	27	54.1	21.4
**8f**	–CH_2_–Tz–nBu	–CH_3_	21	34.1	33.9
**9f**	–CH_2_–Tz–nBu	–C_6_H_5_	21	48.4	23.9
**10f**	–CH_2_–Tz–nBu	NH–C_6_H_5_	26	149.0	7.7

a c_0_ represents the starting concentration of the oNB ester, k is the rate constant, and τ represents the half-life time.

b Irradiation through a window glass plate.

A high-intensity Hg/Xe lamp was used to irradiate the samples. To verify which wavelengths are needed for the photochemical decomposition, a sample of **7e** was also irradiated through a window glass block with a cutoff wavelength of 320 nm. As depicted, the decomposition rate remained constant. Thus, it can be concluded that the longer emission band of the HG/Xe lamp at 365 nm is responsible for the decomposition. Illumination with a 275 nm light-emitting diode (LED) essentially yields the same kinetic behavior, with the same signals appearing in the ^1^H NMR spectra. The only difference was a much slower decomposition due to the lower light intensity of the LED (Figure S16). The explanation is that the 275 nm LED spectrum is broad and contains longer-wavelength light, which then leads to the observed but slower photolysis. An ON/OFF experiment in which an additional waiting time was introduced is shown in Figure S17. Photochemical decomposition does not take place when the sample is not irradiated.

The first interesting aspect is the concentration dependence of photochemical decomposition. Figure 2a depicts the concentration–time profiles for **7e** at three different starting concentrations (25, 50, and 95 mmol L^–1^). The first-order kinetic fit explains the decreasing concentrations well.

**Figure 2:**
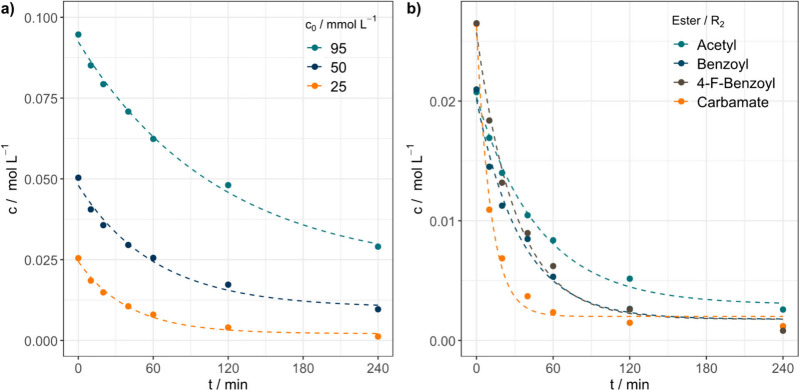
c/t profiles derived from ^1^H NMR spectroscopy for the photochemical decomposition of different oNB esters. a) Concentration-dependent decomposition of **7e**. As expected, lower starting concentrations lead to faster decompositions. b) c/t curves for different oNB ester derivatives. Phenyl carbamate **10f** showed the fastest decomposition.

When looking at the rate constants *k* (41.4 × 10^–5^, 27.1 × 10^–5^, and 14.9 × 10^–5^ s^–1^), it becomes clear that a lower starting concentration leads to faster photochemical decomposition. This finding correlates with an observed discoloration of the solutions with increasing irradiation times (Figure S18). With increasing irradiation time, the UV/vis spectra reveal an emerging strong absorption band centered at 312 nm (Figure S19), which blocks part of the Hg/Xe lamp spectrum (Figure S20). Our explanation for this finding is that the decomposition and its follow-up reactions lead to small concentrations of strongly absorbing molecules. The concentration must be smaller than the limit of detection in ^1^H NMR as no significant new NMR signals could be identified, which could be assigned to the color-intensive impurity. The impurity decreases the number of available photons for the decomposition reaction, which in turn slows down the reaction rate. This result is important for applications in bulk material where the concentration of oNB esters is high and cannot easily be diluted. Materials with higher concentration thus need longer irradiation times for full decomposition. In the context of the present study, lower starting concentrations are needed to disentangle the absorption effect of the impurity from the photochemical behavior of the oNB esters.

Figure 2b illustrates the influence of ester group **R**_**2**_ on the decomposition rate. Acetyl ester **8f** shows a rate constant of 34.1 × 10^–5^ s^–1^, corresponding benzyl ester **9f** decomposed faster with a rate constant of 48.4 × 10^–5^ s^–1^, 4-fluoro-benzyl ester **7f** decomposed with nearly the same rate constant of 54.1 × 10^–5^ s^–1^, and carbamate **10f** showed the highest value for *k* at 149.3 × 10^–5^ s^–1^ in this series. The leaving group thus has a significant influence on the decomposition kinetics. Schmidt et al. reported that for (coumarin-4-yl)­methyl esters the logarithmic rate constant log­(*k*) correlates with the *pK*_*a*_ value of the acid anion that is released.^26^ Figure 3a reveals a similar correlation for oNB esters. Using acids with a low *pK*_*a*_ value as esters thus accelerates the reaction kinetics and leads to faster decomposition of the oNB ester. This observation correlates with the findings of Blanc and Bochet that the H abstraction at the benzyl position is the rate-limiting step.^17^ A more electron-withdrawing ester most likely decreases the stability of the relevant C–H bond, enhancing the radical H-abstraction. By looking at the UV/vis spectra (Figures S21–S33) of the three esters **7f**, **8f**, and **9f** and carbamate **10f**, a simple increase in absorption behavior as the origin of this effect can be ruled out as all three derivatives have comparable absorption spectra and absorption coefficients at the relevant wavelength positions (327, 245, 316, and 253 L cm^–1^ mol^–1^ at 365 nm, respectively; see also Table S1). Therefore, it can be concluded that the esters do not act as simple antennas for the oNB photosystem.

**Figure 3:**
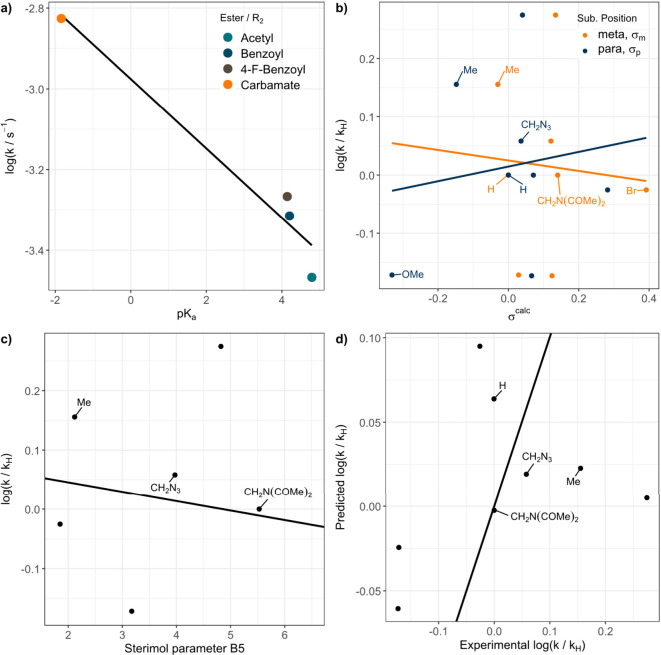
Structure–property relations for the photochemical decomposition of different oNB linkers. a) The log­(*k*/*k*_*H*_)–*pK*_*a*_ plot shows that lower *pK*_*a*_ values of the resulting acid anion led to faster photochemical decompositions. b) Hammet parameters for para (σ_*p*_) and meta positions (σ_*m*_) show only a weak correlation with the logarithmically normalized photochemical decomposition rates log­(*k*/*k*_*H*_ *)*. c) log­(*k*/*k*_*H*_ *)* correlates with the Sterimol parameter *B5*. d) A combined electronic–steric model leads to a reasonable explanation for the observed structure–decomposition rate correlations.

Finally, the influence of the substituent on the phenyl ring of the oNB system is worth investigating. For this, eight oNB acetyl esters **8a**–**f**, **8h**, and **8i** with different substituents were synthesized. To capture the electronic influence, Hammett parameters for para (σ_p_) and meta (σ_m_) positions were used.^27^ Figure 3b shows the correlation between the logarithmic rate constant quotient log­(*k*/*k*_*H*_) and the Hammet parameters σ according to Hammett’s equation (eq 1):^28^

$$\log\left(\frac{k}{k_{H}}\right)=\rho \cdot \sigma$$ 1

As can be seen, there is a weak correlation between the Hammett parameters and the rate constants independent of which type of Hammett parameter (meta vs para position) is used for the analysis. This indicates that electronic parameters are not the main factor driving the different decomposition kinetics. Figure 3b shows that larger, more bulky substituents lead to lower rate constants. Figure 3c tests this hypothesis by plotting the Sterimol parameter *B5* ^29^ against the logarithmic rate constant quotient log­(*k*/*k*_*H*_). As can be seen, a linear trend emerges. Therefore, it can be concluded that the bulkiness of substituent **R**_**1**_ is more important than its electronic influence. This is in line with the observations from Blanc and Bochet^17^ that the intramolecular H abstraction is the rate-limiting step.

To quantify the electronic and steric influence of the substituent **R**_**1**_, a multilinear regression model similar to other linear free-energy relationships like the Taft equation^30^ was built (eq 2):

$$\log\left(\frac{k}{k_{H}}\right)=\rho \cdot \sigma +\delta \cdot B5$$ 2

σ is the Hammett parameter, *B5* is the Sterimol parameter, ρ is the sensitivity factor toward electronic parameters, and δ is the sensitivity factor toward steric parameters. Figure 3d shows a pairs plot of the model and reveals good agreement between the observed and predicted logarithmic rate constant quotient log­(*k*/*k*_*H*_). The model was built with Hammett parameters for para and meta positions of the substituent. σ_*p*_ lead to a better fit result in which the sensitivity toward the Hammett parameter (ρ = 0.157) is positive while the sensitivity toward *B5* (δ = −0.018) is negative. This means that substituents with a large positive Hammett parameter σ_*p*_ accelerate the photochemical cleavage while sterically large substituents inhibit it. As Figure 3 depicts, the correlation is small for both electronic and steric parameters; therefore, it can be concluded that **R**_**1**_ can be chosen freely without influencing the decompositions kinetics strongly.

Taken together, our results show that for a fast photochemical decomposition of oNB esters the ester is the main tuning point. As shown, acids with lower *pK*_*a*_ values lead to faster decompositions. The substituents on the phenyl ring are of lesser importance to the photochemical reaction. While slight electronic and steric influences of the substituents were observed, the photochemical reaction is not strongly influenced by the substituents. Consequently, the substituent position can be used, for example, for cross-linking tasks in polymer networks and can be tuned to these tasks without the fear of altering the decomposition kinetics of the oNB system.

## Conclusions

This study demonstrated that for ortho-nitrobenzyl esters the kinetic rate of photolysis increases as the *pK*_*a*_ of the leaving ester group decreases, highlighting a clear dependence on the leaving group’s acidity. However, it was also found that the reaction rate does not exhibit a straightforward, linear correlation with the nature of the substituents on the phenyl ring of the ortho-nitrobenzyl system. These results suggest that while the intrinsic properties of the leaving group strongly influence the photoreaction kinetics, the effects of ring substituents are more complex. Surprisingly, steric effects dominate electronic effects when it comes to ring substituents. Larger substituents lead to slower decomposition, while less bulky substituents accelerate the photochemical reaction. Our findings contribute to a deeper mechanistic understanding of ortho-nitrobenzyl photochemistry and provide a basis for the rational design of photolabile protecting groups with tailored reactivity. In the future, we are aiming to use multifunctional ortho-nitrobenzyl esters in polymer networks to make these polymer networks photresponsive. Our results support the approach that the ring substituents can be freely used for cross-linking tasks while more care must be taken when altering the structure of the ester.

## Experimental Details

### Photochemistry

NMR measurements were carried out using a Bruker Ascend 500 MHz spectrometer from Bruker Switzerland AG. The instrument was controlled using Bruker’s TopSpin software, version 4.5.0. The acquired spectra were processed with MestReNova 14.3.0 software from Mestrelab Research.^31^ The residual solvent signal of the deuterated solvent was used as an internal reference for the ^1^H and ^13^C chemical shifts.

To investigate the cleavage kinetics of the oNB esters, they were dissolved in deuterated DMSO. The solutions (0.55 mL) were used to fill an NMR tube, and they were irradiated using an LQ-HXP 120-UV/150,26D xenon/mercury-vapor lamp from Leistungselektronik Jena GmbH. The sample was placed 4 cm from the optical fiber cable. Between the irradiation intervals (0, 10, 20, 40, 60, 120, and 240 min of total irradiation time), ^1^H NMR measurements were carried out to observe the decay.

### Data Analysis

The ^1^H NMR spectra were referenced to the residual solvent signal of DMSO-d_6_ and phase and background corrected. Subsequently, spectra were normalized to a characteristic peak of the starting material (4-fluorbenzoates **7**: 1.70–1.78 ppm; acetates **8**: 1.95–2.07 ppm; benzoate **9f**: 1.66–1.72 ppm; and carbamate **10f**: 7.59–7.62 ppm), and the signals of interest for the educt and the acid anion (4-fluorbenzoate **7e**: 7.30–7.35 ppm; **7f**: 7.35–7.40 ppm; acetates **8**: 1.88–1.95 ppm; benzoate **9f**: 7.47–7.53 ppm; and carbamate **10f**: 6.52–6.59 ppm (overlaps with the educt signal, which is corrected by subtracting the integral value of the educt)) were integrated. This approach is similar to the one chosen by Kim and Diamond to analyze the photolysis kinetics of oNB ethers.^32^ From the peak areas *A*_*E*_ (peak of the oNB ester educt) and *A*_*AA*_ (peak of the cleaved of acid anion) the concentrations *c*_*E*_ and *c*_*AA*_ were calculated using the respective molar masses under the assumption that the total mass of the material—educt and product combined—did not change as the NMR tube was sealed for the entirety of the measurement series (eq 3). Thus, the starting concentration *c*_0_ can be used:

$$c_{E}=\frac{A_{E}}{A_{E}+A_{AA}}\cdot c_{0}$$ 3

For the kinetic analysis, the curves were fitted with a first-order kinetic rate equation (eq 4) for the educt

$$c_{E}\left(t\right)=c_{E}\left(t=0\right)\cdot e^{-kt}$$ 4

and the acid anion (eq 5)­

$$c_{AA}\left(t\right)=c_{E}\left(t=0\right)\cdot \left(1-e^{-kt}\right)+c_{AA}\left(t=0\right)$$ 5

in which *t* is the time and *k* is the apparent rate constant. *k* is a product of the photo flux density which is a constant in all experiments and the molecule specific decomposition rate of interest. The resulting rate constants *k* are listed.

From the rate constant *k*, the half-life time τ can be calculated as follows using eq 6:

$$\tau =\frac{\ln\left(2\right)}{k}$$ 6

The data analysis was done in R (4.4.3)^33^ using its packages tidyverse,^34^ readxl,^35^ colorspace,^36,37^ reshape2,^38^ ggrepel,^39^ and cowplot.^40^ The *pK*_*a*_ values were predicted using ACDLabs PhysChem Suite,^41^ as phenylcarbamic acid is unstable. The Sterimol parameters^29^ were calculated using python package morfeus^42^ and R package reticulate^43^ after optimizing the substituent structures using the 3D structure generation implemented in OpenBabel (3.1.1),^44^ which uses MMFF94 force fields.^45^

## Supplementary Material



## Notes

References 11, 24, 25, 27, and 46; additional synthesis protocols; concentration–time profiles; and UV/vis and IR spectra (PDF)

## Abbreviations

Tz: 1,4-Triazole
oNB: ortho-nitrobenzyl
NMR: Nucelar Magnetic Resonance
IR: Infrared
HPLC: High-Performance Liquid Chromatography.
