# Supplementary material for: Photolysis of ortho-Nitrobenzyl Esters: Kinetics and Substituent Effects
Source: ACS Omega. 2025 Nov 20;10(47):57560–7. doi: 10.1021/acsomega.5c08422 (PMC12676297; doi:10.1021/acsomega.5c08422)
Supplement: Supplementary file 1 [file ao5c08422_si_001.pdf]

Photolysis of ortho-Nitrobenzyl Esters: Kinetics and Substituent Effects

Anthony L. Fink[1], Alexander G. Groß[1], Florian Puch[2,3] and Robert Geitner[1,]*

[1]Group of Physical Chemistry/Catalysis, Department of Natural Sciences and Mathematics, Technische Universität Ilmenau, Weimarer Str. 32, 98693 Ilmenau, Germany

[2]Plastics Technology Group, Department of Mechanical Engineering, Thuringian Center of Innovation in Mobility, Technische Universität Ilmenau, Gustav-Kirchhoff Str. 5, 98693 Ilmenau, Germany

[3]Thüringisches Institut für Textil- und Kunststoff-Forschung e.V., Breitscheidstr. 97, 07407 Rudolstadt, Germany

*e-mail: robert.geitner@tu-ilmenau.de

1. Synthesis protocols

All commercially obtained reagents were used without further purification unless otherwise noted. 1-(2-Nitrophenyl)ethanol and 5-methyl-2-nitrobenzoic acid were purchased from BLDpharm. 1-(5-Methoxy-2-nitrophenyl)ethanol and 1-(5-bromo-2-nitrophenyl)ethanol were obtained from Aurora Fine Chemicals. 1-Hexyne and magnesium ethoxide were purchased from Thermo Fisher Scientific. Sodium borohydride and sodium azide were obtained from Merck. Triphenylphosphine was purchased from Carl ROTH. Thionyl chloride, diethyl malonate, phenyl isocyanate and benzoyl peroxide were obtained from Sigma-Aldrich. 4-Fluorobenzoic anhydride was synthesized from 4-fluorobenzoic acid, which was purchased from BLDpharm. For flash chromatography silica gel (particle size 40-63 µm), purchased from VWR Chemicals, was used. The TLC plates used were ALUGRAM® SIL G $_{UV254}$ MACHEREY-NAGEL.

NMR measurements were carried out using a Bruker Ascend 500 MHz spectrometer from Bruker Switzerland AG. The instrument was controlled using Bruker's TopSpin software, version 4.5.0. The acquired spectra were processed with MestReNova 14.3.0 software from Mestrelab Research. For this, the recorded free induction decays were Fourier-transformed, followed by baseline and phase correction. The residual solvent signals of the deuterated solvents were used as internal references for the ^{1}H and ^{13}C chemical shifts. HPLC analysis was performed using a Dionex UltiMate RS pump from Thermo Scientific in combination with a Kinetex 2.6 µm XB-C18 100 Å 100 × 2.1 mm column from Phenomenex Inc. The HPLC system was coupled to an Orbitrap Exploris 240 mass spectrometer from Thermo Scientific, enabling mass spectroscopic analysis of the separated substances. The UV spectra were recorded in acetonitrile using a Specord 200 UV/Vis-spectrometer from Analytik Jena. FTIR spectra were recorded using a Cary 630 FTIR spectrometer from Agilent Technologies.

5-Methyl-2-nitrobenzoylchloride (**2**)[1]

1 (15 g, 82.8 mmol) and thionyl chloride (90 ml), DMF (0.5 ml) were added to a round-bottom flask. A reflux condenser and a drying tube charged with anhydrous calcium chloride were added. The mixture was refluxed for 3 h. The excess thionyl chloride was then distilled off. The residue was dissolved in diethyl ether and washed with water and brine. The aqueous phases were combined and extracted with diethyl ether (50 ml). The organic phases were combined and dried over anhydrous magnesium sulfate. The solvent was then removed in vacuo. The product **2** was obtained as a yellow oil which slowly crystallized with a yield of 15.034 g (90%). **^{1}H NMR** (500 MHz, $CDCl_3$, 25 °C): δ = 8.02 (d, *J* = 8,37 Hz, 1H, 3-Ph*H*); 7.49 (d, *J* = 8.37 Hz, 1H, 4-Ph*H*); 7.45 (d, *J* = 1.86 Hz, 1H, 6-Ph*H*); 2.53 (s, 3H, CH_3); **^{13}C NMR** (126 MHz, $CDCl_3$, 25 °C): δ = 166.1 (*C*OCl); 146.2 (5-*Ph*CH_3); 142.8 (2-*Ph*NO_2); 133.0 (4-*Ph*H); 132.7 (1-*Ph*COCl); 128.5 (6-*Ph*H); 124.7 (3-*Ph*H); 21.6 (5-PhCH_3).

1-(5-Methyl-2-nitrophenyl)ethan-1-one (**3**)[2]

To an oven-dried three-necked round-bottom flask magnesium ethoxide (9.48 g, 82.9 mmol) was added. After the flask was charged with argon tetrahydrofuran (76ml) and diethylmalonate (12.64 ml, 82.9 mmol) were added. The reaction mixture was refluxed for 4 h before **2** (15.034 g, 75.3 mmol) was slowly added as a solution in tetrahydrofuran (38 ml). Heating was continued for another 20 min before the solvent was removed in vacuo. The residue was dissolved in ethyl acetate (200 ml) and washed with dilute sulfuric acid (10 ml concentrated sulfuric acid in 100 ml water). The aqueous phase was extracted with ethyl acetate (50 ml) before the combined organic phases were washed with brine and dried over magnesium sulfate. The solvent was then removed in vacuo. To the residue glacial acetic acid (20 ml) and sulfuric acid (3.8 ml) were added and the mixture was refluxed for 1.5 h (until the evolution of carbon dioxide stopped). The mixture was then cooled on an ice bath and alkalized using a precooled potassium

hydroxide solution. The resulting suspension was filtered, and the filtrate was extracted using diethyl ether and dried using brine and magnesium sulfate before the solvent was removed in vacuo to give the product as a brown oil (8.783 g). After the filter residue was dried it was extracted with diethyl ether. The solvent was removed in vacuo, and the product was obtained as a brown oil (0.954 g). The product was used without further purification, and the total yield of this reaction was 9.737 g (72%). **^{1}H NMR** (500 MHz, $CDCl_3$, 25 °C): δ = 8.02 (d, *J* = 8.39 Hz, 1H, 3-Ph*H*); 7.36 (d, *J* = 8.39 Hz, 1H, 4-Ph*H*); 7.17 (s, 1H, 6-Ph*H*); 2.53 (s, 3H, 5-PhCH_3); 2.47 (s, 3H, 1-PhCOCH_3); **^{13}C NMR** (126 MHz, $CDCl_3$, 25 °C): δ = 200.5 (*C*OCH_3); 146.2 (5*Ph*CH_3); 143.5 (2*Ph*NO_2); 138.6 (1-*Ph*COCH_3); 131.0 (4-*Ph*H); 127.7 (6-*Ph*H); 124.6 (3-*Ph*H); 30.5 (1-PhCOCH_3); 21.7 (5-PhCH_3).

1-(5-(Bromomethyl)-2-nitrophenyl)ethan-1-one (**4**)[1]

To a three-necked round-bottom flask *N*-bromosuccinimide (4.83 g, 27.1 mmol) was added. After the apparatus was flushed with argon, **3** (4.417 g, 24.7 mmol) dissolved in carbon tetrachloride (44 ml) and benzoyl peroxide (302 mg, 1.2 mmol) were added. The reaction mixture was refluxed for 6 h and subsequently filtered. After the solvent was distilled off the residue was dissolved in dichloromethane and washed with water and brine. The aqueous phases were extracted again with dichloromethane. The organic phases were dried over magnesium sulfate, and the solvent was removed in vacuo. The raw product (5.734 g) was further purified by recrystallisation from a mixture of dichloromethane and hexane. The Product **4** was obtained as a yellow crystalline solid with a yield of 2.478 g (39%). **^{1}H NMR** (500 MHz, $CDCl_3$, 25 °C): δ = 8.08 (d, *J* = 8.39 Hz, 1H, 3-Ph*H*); 7.60 (d, *J* = 8.54 Hz, 1H, 4-Ph*H*); 7.42 (s, 1H, 6-Ph*H*); 4.49 (s, 2H, CH_2Br); 2.56 (s, 3H, CH_3); **^{13}C NMR** (126 MHz, $CDCl_3$, 25 °C): δ = 199.5 (1-Ph*C*OCH_3); 145.2 (2-*Ph*NO_2); 144.9 (5-*Ph*CH_2Br); 138.8 (1-*Ph*COCH_3); 131.1 (4-*Ph*H); 127.9 (6-*Ph*H); 125.1 (3-*Ph*H); 30.4 (1-PhCOCH_3); 30.3 (CH_2Br).

1-(5-(Bromomethyl)-2-nitrophenyl)ethan-1-ol (**5**)[1]

To a round-bottom flask **4** (2.868 g, 11.1 mmol), methanol (30 ml) and 1,4-dioxane (21.5 ml) were added. The mixture was cooled on an ice bath and sodium borohydride (420 mg, 11.1 mmol) was added under vigorous stirring. After the mixture was stirred for 2 h, acetone was added to quench the excess sodium borohydride. Then the solvent was removed in vacuo, and the residue was dissolved in chloroform (75 ml) and washed with brine (75 ml). The aqueous phase was back-extracted with chloroform (50 ml). The combined organic phases were dried over magnesium sulfate, and the solvent was removed in vacuo. The product **5** was obtained as a brownish yellow oil with a yield of 2.775 g (96%); **^{1}H NMR** (500 MHz, $CDCl_3$, 25 °C): δ = 7.91 (d, *J* = 8.43 Hz, 1H, 3-Ph*H*); 7.87 (s, 1H, 6-Ph*H*); 7.45 (dd, *J* = 8.36; 2.22 Hz, 1H, 4-Ph*H*); 5.46 (q, *J* = 6.34 Hz, 1H, C*H*OH); 4.51 (s, 2H, C*H*$_2$Br); 2.48 (s, 1H, O*H*); 1.58 (m, 3H, C*H*$_3$); **^{13}C NMR** (126 MHz, $CDCl_3$, 25 °C): δ = 147.1 (2-*Ph*NO_2); 143.9 (5-*Ph*CH_2Br); 141.9 (1-*Ph*CHOH); 128.7 (4-*Ph*H); 128.3 (6-*Ph*H); 125.2 (3-*Ph*H); 65.6 (*C*HOH); 31.3 (*C*H_2Br); 24.5 (*C*H_3).

1-(5-methyl-2-nitrophenyl)ethan-1-ol (**6b**)

3 (545 mg, 3mmol), methanol (6 ml), 1,4-dioxane (4 ml) and sodium borohydride (173 mg, 4,6 mmol) were added to a round-bottom flask. After the mixture was stirred for 30 min at room temperature, dilute hydrochloric acid (0,1 ml concentrated hydrochloric acid and 5,6 ml water) was added. Ethyl acetate (20 ml) and brine (10 ml) were added, and the phases were separated. The aqueous phase was extracted again with ethyl acetate (20 ml). The combined organic phases were washed with brine (20 ml) and dried over magnesium sulfate. The solvent was removed in vacuo to obtain the product **6b** as a yellow oil with a yield of 440 mg (80 %). **^{1}H NMR** (500 MHz, DMSO-d6, 25 °C): δ = 7.81 (d, *J* = 8.24 Hz, 1H, 3-Ph*H*); 7.64 (d, *J* = 1.94

Hz, 1H, 6-Ph*H*); 7.29 (dd, *J* = 8.39; 1.95 Hz, 1H, 4-Ph*H*); 5.47 (d, *J* = 4.30 Hz, 1H, CHO*H*); 5.16 (qd, *J* = 6.35, 4.42 Hz, 1H, C*H*OH); 2.41 (s, 3H, CH_3); 1.36 (d, *J* = 6.27 Hz, 3H, CH_3); **^{13}C NMR** (126 MHz, DMSO-d6, 25 °C): δ = 145.1 (2-*Ph*NO_2); 144.2 (5-*Ph*CH_2N_3); 142.3 (1-*Ph*$CHOHCH_3$); 128.3 (4-*Ph*H); 128.0 (6-*Ph*H); 124.0 (3-*Ph*H); 63.8 (1-Ph*C*$HOHCH_3$); 25.2 (1-PhCHOH*C*H_3); 21.2 (CH_3).

1-(5-(Azidomethyl)-2-nitrophenyl)ethan-1-ol (**6e**)[3]

To a round-bottom flask **5** (3.515 g, 13.5 mmol), acetone (72 ml), water (14.5 ml) and sodium azide (1.318 g, 20.3) were added. After the mixture was refluxed for 18 h, the acetone was removed in vacuo. Ethyl acetate (100 ml) and brine (50 ml) were added, and the phases were separated. The aqueous phase was extracted with ethyl acetate (50 ml), and the combined organic phases were dried over magnesium sulfate. The solvent was removed in vacuo to obtain the crude product (3.282 g) which was further purified by flash column chromatography with ethyl acetate/hexane (1:3). The product **6e** was obtained as an orange oil with a yield of 1.222 g (41%). **^{1}H NMR** (500 MHz, $CDCl_3$, 25 °C): δ = 7.93 (d, *J* = 8.40 Hz, 1H, 3-Ph*H*); 7.79 (s, 1H, 6-Ph*H*); 7.36 (dd, *J* = 8.40; 2.00 Hz, 1H, 4-Ph*H*); 5.44 (q, *J* = 6.37 Hz, 1H, C*H*OH); 4.48 (s, 2H, CH_2N_3); 2.52 (s, 1H, O*H*); 1.56 (d, *J* = 6.37 Hz, 3H, CH_3); **^{13}C NMR** (126 MHz, $CDCl_3$, 25 °C): δ = 147.2 (2-*Ph*NO_2); 142.0 (1-*Ph*$CHOHCH_3$); 141.8 (5-*Ph*CH_2N_3); 127.4 (4-*Ph*H); 127.0 (6-*Ph*H); 125.2 (3-*Ph*H); 65.6 (1-Ph*C*$HOHCH_3$); 54.0 (CH_2N_3); 24.5 (1-PhCHOH*C*H_3); **^{15}N NMR** (51 MHz, $CDCl_3$, 25 °C): δ = 373.5 (*N*O_2); 247.6 (C-N=*N*=N); 214.8 (C-N=N=*N*); 73.4 (C-*N*=N=N); **HRMS** (ESI, m/z) calcd for $C_9H_9N_4O_2$ $[M-OH]^+$: 205.0721, found: 205.0717.

1-(5-((4-Butyl-1H-1,2,3-triazole-1-yl)methyl)-2-nitrophenyl)ethan-1-ol (**6f**)

To a round-bottom flask **6e** (374 mg, 1.7 mmol), acetonitrile (30 ml), 1-hexyne (0.4 ml, 3.4 mmol), triethylamine (0.7 ml, 5 mmol) and copper(I) chloride (8 mg, 0.08 mmol) were added. The reaction mixture was stirred at room temperature for 4.5 h, after which the solvent was removed in vacuo. The residue was dissolved in ethyl acetate (75 ml) and washed with brine (75 ml). The aqueous phase was extracted with brine (75 ml), and the combined organic phases were dried over magnesium sulfate. The solvent was removed in vacuo to obtain the crude product as a brownish yellow oil (517 mg). The crude product was further purified using flash column chromatography with ethyl acetate/hexane (1:1) to yield 254 mg (50 %) of **6f** as a slightly yellow oil with high viscosity. **^{1}H NMR** (500 MHz, DMSO-d6, 25 °C): δ = 7.96 (s, 1H, 5-1,2,3-triazol*H*); 7.89 (d, *J* = 8.31 Hz, 1H, 3-Ph*H*); 7.72 (d, *J* = 2.03 Hz, 1H, 6-Ph*H*); 7.30 (dd, *J* = 8.40; 1.99 Hz, 1H, 4-Ph*H*); 5.70 (d, *J* = 15.89 Hz, 1H, C*H*$_2$N); 5.67 (d, *J* = 15.89 Hz, 1H, C*H*$_2$N); 5.54 (d, *J* = 4.20 Hz, 1H, CHO*H*); 5.11 (qd, 1H, *J* = 6.25; 4.02 Hz, C*H*OH); 2.62 (t, *J* = 7.60 Hz, 2H, C*H*$_2$CH$_2$CH$_2$CH$_3$); 1.57 (p, *J* = 7.52 Hz, 2H, CH$_2$C*H*$_2$CH$_2$CH$_3$); 1.34 (d, *J* = 6.35 Hz, 3H, C*H*$_3$); 1.29 (m, 2H, CH$_2$CH$_2$C*H*$_2$CH$_3$); 0.88 (t, *J* = 7.35 Hz, 3H, CH$_2$CH$_2$CH$_2$C*H*$_3$); **^{13}C NMR** (126 MHz, DMSO-d6, 25 °C): δ = 147.4 (4-1,2,3-triazol); 146.8 (2-*Ph*NO$_2$); 142.4 (1-*Ph*CHOHCH$_3$); 141.8 (5-*Ph*CH$_2$N); 127.0 (4-*Ph*H); 126.9 (6-*Ph*H); 124.4 (3-*Ph*H); 122.3 (5-1,2,3-triazolH); 63.7 (*C*HOHCH$_3$); 51.9 (*C*H$_2$N); 31.3 (CH$_2$*C*H$_2$CH$_2$CH$_3$); 25.0 (CHOH*C*H$_3$); 24.7 (*C*H$_2$CH$_2$CH$_2$CH$_3$); 21.7 (CH$_2$CH$_2$*C*H$_2$CH$_3$); 13.7 (CH$_2$CH$_2$CH$_2$*C*H$_3$); **^{15}N NMR** (51 MHz, DMSO-d6, 25 °C): δ = 375.7 (*N*O$_2$); 361.7 (2-1,2,3-triazol); 353.8 (3-1,2,3-triazol); 245.7 (1-1,2,3-triazol); **HRMS** (ESI, m/z): calcd for $C_{15}H_{21}N_4O_3$ $[M+H]^+$ = 305.1609, found: 305.1600; **IR**: 755 (w), 787 (m), 839 (m), 1029 (w), 1053 (m), 1108 (m), 1156 (w), 1219 (w), 1342 (m), 1457 (m), 1520 (vs), 2860 (w), 2927 (w), 2959 (w), 3078 (w), 3133 (w), 3288 (w) cm^{-1}.

1-(5-amino-2-nitrophenyl)ethan-1-ol (**6g**)[1]

To a round-bottom flask **6e** (2.024 g, 9.2 mmol), tetrahydrofuran (30.5 mL), water (511 µL) and triphenylphosphine (2.651 g, 10.1 mmol) were added. The reaction mixture was stirred at 60 °C for 4 h. The solvent was removed in vacuo to obtain the crude product as a brown oil (4.777 g). The crude product was further purified by flash column chromatography with MeOH/DCM (1:5). The product **6g** was obtained as a yellow solid with a yield of 848 mg (48 %). **^{1}H NMR** (500 MHz, DMSO-d6, 25 °C): δ = 7.84 (d, *J* = 8.37 Hz, 1H, 3-Ph*H*); 7.79 (d, *J* = 1.92 Hz, 1H, 6-Ph*H*); 7.44 (dd, *J* = 8.42; 1.93 Hz, 1H, 4-Ph*H*); 5.49 (s, 1H, O*H*); 5.16 (q, *J* = 6.31 Hz, 1H, C*H*OH); 3.81 (s, 2H, CH_2NH_2); 1.37 (d, *J* = 6.33 Hz, 3H, CH_3); **^{13}C NMR** (126 MHz, DMSO-d6, 25 °C): δ = 150.3 (5-*Ph*CH_2NH_2); 145.6 (2-*Ph*NO_2); 142.0 (1-*Ph*$CHOHCH_3$); 126.2 (4-*Ph*H); 126.0 (6-*Ph*H); 123.8 (3-*Ph*H); 63.9; 45.2; 25.2; **HRMS** (ESI. m/z): calcd for $C_9H_{13}N_2O_3$ $[M+H]^+$ = 197.0921, found: 197.0917.

1-(5-azido-2-nitrophenyl)ethyl 4-fluorobenzoate (**7e**)

To an oven-dried round-bottom flask **6e** (266 mg, 1.2 mmol), ketylated tetrahydrofuran (30 mL), 4-fluorobenzoic anhydride (471 mg, 1.8 mmol) and 4-dimethylaminopyridine (16 mg, 0.13 mmol) were added. The flask was equipped with an oven-dried reflux condenser and a drying tube charged with calcium chloride. The reaction mixture was stirred under reflux overnight (17 h). The solvent was removed in vacuo, and the residue was dissolved in ethyl acetate. The organic phase was washed with water, and the aqueous phase was back-extracted with ethyl acetate. The combined organic phases were then washed with brine and dried over magnesium sulfate. The solvent was removed in vacuo to obtain the crude product (629 mg). The crude product was further purified by flash column chromatography using ethyl acetate/hexane (1:5). The product (**7e**) was obtained as a pale yellow waxy solid with a yield of 191 mg (46%). **^{1}H NMR** (500 MHz, $CDCl_3$, 25 °C): δ = 8,07 (dd, *J* = 8,65; 5,58 Hz, 2H, 2,6-

p-fluorophenyl*H*); 8,00 (d, *J* = 8,39 Hz, 1H, 3-Ph*H*); 7,62 (d, *J* = 1,96 Hz, 1H, 6-Ph*H*); 7,39 (dd, *J* = 8,46; 1,94 Hz, 1H, 4-Ph*H*); 7,13 (t, *J* = 8,60 Hz, 2H, 3,5-*p*-fluorophenyl*H*); 6,55 (q, *J* = 6,53 Hz, 1H, C*H*OR); 4,47 (d, *J* = 14,82 Hz, 1H, CH_2N_3); 4,44 (d, *J* = 14,82 Hz, 1H, CH_2N_3); 1,79 (d, *J* = 6,55 Hz, 3H, CH_3); **^{13}C NMR** (126 MHz, $CDCl_3$, 25 °C): δ = 166,1 (d; *J* = 255 Hz, 4-*p*-fluorophenyl); 164,6 (*C*OOR); 147,3 (2-*Ph*NO_2); 141,9 (5-*Ph*CH_2N_3); 138,3 (1-*Ph*$CHORCH_3$); 132,4 (d; *J* = 9 Hz, 2,6-*p*-fluorophenyl); 127,8 (4-*Ph*H); 126,3 (6-*Ph*H); 126,1 (d; *J* = 3 Hz, 1-*p*-fluorophenyl); 125,4 (3-*Ph*H); 115,8 (d; *J* = 22 Hz, 3,5-*p*-fluorophenyl); 69,0 ($CHORCH_3$); 53,8 (CH_2N_3); 22,2 ($CHORCH_3$); (470 MHz, $CDCl_3$, 25 °C): δ = -104,8; **^{15}N NMR** (51 MHz, $CDCl_3$, 25 °C): δ = 371,8 (*N*O_2); 247,4 (C-N=*N*=N); 72,9 (C-*N*=N=N); **HRMS** (ESI, m/z): calcd for $C_9H_9N_4O_2$ $[M-C_7H_4OF]^+$: 205.0721, found: 205.0719; **IR**: 806 (m), 834 (vs), 846 (s), 859 (s), 879 (w), 929 (w), 968 (m), 977 (w), 1013 (m), 1020 (m), 1062 (m), 1080 (m), 1096 (m), 1111 (m), 1126 (m), 1157 (vs), 1171 (w), 1198 (m), 1230 (vs), 1247 (s), 1273 (vs), 1316 (s), 1341 (m), 1352 (m), 1416 (w), 1452 (w), 1506 (s), 1521 (vs), 1601 (m), 1708 (m), 2069 (w), 2096 (m), 2875 (vw), 2937 (w), 3003 (w), 3065 (w) cm^{-1}.

1-(5-((4-butyl-1H-1,2,3-triazol-1-yl)methyl)-2-nitrophenyl)ethyl 4-fluorobenzoate (**7f**)

To an oven-dried round-bottom flask **6f** (223 mg, 0,7 mmol), ketylated tetrahydrofuran (30 mL), 4-fluorobenzoic anhydride (292 mg, 1.1 mmol) and 4-dimethylaminopyridine (10 mg, 0.11 mmol) were added. The flask was equipped with an oven-dried reflux condenser and a drying tube charged with calcium chloride. The reaction mixture was stirred under reflux overnight (18 h). The solvent was removed in vacuo, and the residue was dissolved in ethyl acetate (30 ml). The organic phase was washed with water (2 x 30 ml), and the aqueous phase was back-extracted with ethyl acetate (30 ml). The combined organic phases were then washed with brine (30 ml) and dried over magnesium sulfate. The solvent was removed in vacuo to

obtain the crude product (399 mg). The crude product was further purified by flash column chromatography using ethyl acetate/hexane (1:1). The product (**7f**) was obtained as a yellow solid with a yield of 104 mg (33%) and a purity of 84 %. **^{1}H NMR** (500 MHz, DMSO-d6, 25 °C): δ = 8.01 (m, 3H, 3-Ph*H*, 2,6-*p*-fluorophenyl*H*); 7.91 (s, 1H, 5-1,2,3-triazole*H*); 7.67 (d, *J* = 1.87 Hz, 1H, 6-Ph*H*); 7.38 (m, 3H, 6-Ph*H*, 3,5-*p*-fluorophenyl*H*); 6.32 (q, *J* = 6.44 Hz, 1H, C*H*OR); 5.73 (d, *J* = 15.65 Hz, 1H, CH_2N); 5.69 (d, *J* = 15.70 Hz, 1H, CH_2N); 2.53 (m, 2H, $CH_2CH_2CH_2CH_3$); 1.68 (d, *J* = 6.53 Hz, 3H, $CHORCH_3$); 1.47 (pd, *J* = 7.17; 2.48 Hz, 2H, $CH_2CH_2CH_2CH_3$); 1.26 (h, *J* = 7.29 Hz, 2H, $CH_2CH_2CH_2CH_3$); 0.85 (t, *J* = 7.35 Hz, 3H, $CH_2CH_2CH_2CH_3$); **^{13}C NMR** (126 MHz, DMSO-d6, 25 °C): δ = 171.2 (d; *J* = 212 Hz, 4-*p*-fluorophenyl); 163.9 (*C*OOR); 147.5 (4-1,2,3-triazole); 146.8 (2-*Ph*NO_2); 142.9 (5-*Ph*CH_2N); 137.2 (1-*Ph*$CHORCH_3$); 132.3 (d; *J* = 10 Hz, 2,6-p-fluorophenyl); 128.1 (4-*Ph*H); 126.3 (6-*Ph*H); 125.8 (1-*p*-fluorophenyl); 125.0 (3-*Ph*H); 122.3 (5-1,2,3-triazole); 116.0 (d; *J* = 22 Hz, 3,5-*p*-Fluorphenyl); 68,3 (*C*$HORCH_3$); 51.7 (*C*H_2N); 31.0 ($CH_2CH_2CH_2CH_3$); 24.6 ($CH_2CH_2CH_2CH_3$); 21.7 ($CH_2CH_2CH_2CH_3$); 21.5 ($CHORCH_3$); 13.7 ($CH_2CH_2CH_2CH_3$); **^{15}N NMR** (51 MHz, DMSO-d6, 25 °C): δ = 374.0 (*N*O_2); 362.1 (2-1,2,3-triazole); 354.2 (3-1,2,3-triazole); 245.2 (1-1,2,3-triazole); **HRMS** (ESI, m/z): calcd for $C_{22}H_{24}FN_4O_4$ $[M+H]^+$: 427.1777, found: 427.1767; **IR**: 690 (m), 722 (w), 740 (m), 768 (vs), 786 (w), 806 (w), 838 (m), 854 (m), 879 (w), 919 (w), 1017 (m), 1047 (m), 1061 (m), 1077 (m), 1090 (m), 1108 (m), 1128 (m), 1152 (m), 1176 (w), 1228 (m), 1268 (vs), 1327 (m), 1355 (m), 1411 (w), 1431 (w), 1457 (w), 1507 (m), 1523 (m), 1604 (m), 1702 (m), 2855 (w), 2871 (w), 2928 (w), 2956 (w), 3002 (w), 3066 (w), 3143 (vw) cm^{-1}.

8a-f,h,i

To a round-bottom flask the alcohol **6a-g**, acetic anhydride (10 ml) and DMAP (0,1 eq.) were added. The mixture was heated to 100 °C overnight. The excess acetic anhydride was removed

in vacuo. The residue was dissolved in ethyl acetate (30 ml) and washed twice with brine (2 x 30 ml). The aqueous phases were extracted with ethyl acetate (30 ml). Then the organic phases were dried over magnesium sulfate, and the solvent was removed in vacuo to obtain the crude product as brown oil.

1-(2-nitrophenyl)ethyl acetate (**8a**)

Starting material: **6a** (3.8 mmol): purification: flash column chromatography using ethyl acetate/hexane (1:3); yield: 80 %; **^{1}H NMR** (500 MHz, DMSO-d6, 25 °C): δ = 7.96 (dd, *J* = 8.22, 1.30 Hz, 1H, 3-Ph*H*); 7.77 (td, *J* = 7.57, 1.33 Hz, 1H, 5-Ph*H*); 7.72 (dd, *J* = 7.91; 1.53 Hz, 1H, 6-Ph*H*); 7.57 (ddd, *J* = 8.53; 7.19; 1.52 Hz, 1H, 4-Ph*H*); 6.06 (q, *J* = 6.55 Hz, 1H, C*H*OR); 2.01 (s, 3H, CH_3COOR); 1.57 (d, *J* = 6.55 Hz, 3H, CHORCH_3); **^{13}C NMR** (126 MHz, DMSO-d6, 25 °C): δ = 169,8 (CH_3*C*OOR); 147.7 (2-*Ph*NO_2); 136.7 (1-*Ph*CHOHCH_3); 134.1 (5-*Ph*H); 129.1 (4-*Ph*H); 127.5 (6-*Ph*H); 124.2 (3-*Ph*H); 67.3 (1-Ph*C*HORCH_3); 21.4 (1-PhCHOH*C*H_3); 20.8 (*C*H_3COOR); **HRMS** (ESI, m/z) calcd for $C_8H_8NO_2$ [M-CH_3COO]$^+$: 150.0550, found: 150.0547; **IR**: 678 (m), 708 (m), 748 (m), 790 (m), 858 (m), 941 (m), 1019 (m), 1059 (m), 1150 (w), 1200 (m), 1232 (vs), 1302 (m), 1347 (m), 1445 (m), 1521 (vs), 1580 (w), 1612 (w), 1742 (s), 2871 (w), 2936 (w), 2984 (w) cm^{-1}.

1-(5-methyl-2-nitrophenyl)ethyl acetate (**8b**)

Starting material: **6b** (2.4 mmol); purification: flash column chromatography using ethyl acetate/hexane (1:3); yield: 84 %; **^{1}H NMR** (500 MHz, DMSO-d6, 25 °C): δ = 7.89 (d, *J* = 8.32 Hz, 1H, 3-Ph*H*); 7.51 (d, *J* = 1.90 Hz, 1H, 6-Ph*H*); 7.36 (dd, *J* = 8.45; 1.88 Hz, 1H, 4-Ph*H*); 6.10 (q, *J* = 6.52 Hz, 1H, C*H*OR); 2.43 (s, 3H, 5-PhCH_3); 2.03 (s, 3H, CH_3COOR); 1.55

(d, $J = 6.53$ Hz, 3H, CHORCH_3); **^{13}C NMR** (126 MHz, DMSO-d6, 25 °C): δ = 169,7 (CH_3*C*OOR); 145.3 (2-*Ph*NO_2); 145.0 (5-*Ph*CH_3); 137.0 (1-*Ph*CHOHCH_3); 129.4 (4-*Ph*H); 127.5 (6-*Ph*H); 124.3 (3-*Ph*H); 67.2 (1-Ph*C*HORCH_3); 21.4 (1-PhCHOH*C*H_3); 21.1 (1-Ph*C*H_3); 20.8 (*C*H_3COOR); **HRMS** (ESI, m/z) calcd for $C_9H_{10}NO_2$ [M-CH_3COO]$^+$: 164.0707, found: 164.0704; **IR**: 706 (m), 734 (w), 756 (m), 836 (s), 856 (w), 907 (m), 955 (m), 1033 (s), 1059 (m), 1079 (m), 1110 (w), 1168 (m), 1240 (vs), 1339 (m), 1355 (m), 1371 (m), 1447 (w), 1509 (s), 1588 (w), 1608 (w), 1726 (m), 2845 (w), 2936 (w), 2984 (w), 3004 (w) cm^{-1}.

1-(5-methoxy-2-nitrophenyl)ethyl acetate (**8c**)

Starting material: **6c** (1.9 mmol); purification: flash column chromatography using ethyl acetate/hexane (1:3); yield: 77 %; **^{1}H NMR** (500 MHz, DMSO-d6, 25 °C): δ = 8.07 (dd, $J = 8.55$; 0.82 Hz, 1H, 3-Ph*H*); 7.09 (m, 2H, 6-Ph*H*, 4-Ph*H*); 6.20 (q, $J = 6.48$ Hz, 1H, C*H*OR); 3.90 (s, 3H, OCH_3); 2.05 (s, 3H, CH_3COOR); 1.55 (d, $J = 6.49$ Hz, 3H, CHORCH_3); **^{13}C NMR** (126 MHz, DMSO-d6, 25 °C): δ = 169,8 (CH_3*C*OOR); 163,6 (5-*Ph*OCH_3); 140.7 (1-*Ph*CHOHCH_3); 140.1 (2-*Ph*NO_2); 127.5 (3-*Ph*H); 113.5 (4-*Ph*H); 112.2 (6-*Ph*H); 67.5 (1-Ph*C*HORCH_3); 56.2 (O*C*H_3); 21.4 (1-PhCHOH*C*H_3); 20.8 (*C*H_3COOR); **HRMS** (ESI, m/z) calcd for $C_9H_{10}NO_3$ [M-CH_3COO]$^+$: 180.0656, found: 180.0652; **IR**: 706 (w), 758 (m), 834 (m), 883 (w), 905 (w), 949 (m), 1023 (m), 1057 (m), 1090 (m), 1170 (m), 1194 (m), 1232 (vs), 1290 (m), 1321 (m), 1337 (m), 1369 (m), 1425 (m), 1457 (m), 1483 (m), 1511 (m), 1578 (m), 1612 (m), 1740 (m), 2843 (w), 2940 (w), 2980 (w) cm^{-1}.

1-(5-bromo-2-nitrophenyl)ethyl acetate (**8d**)

Starting material: **6d** (1.4 mmol); purification: flash column chromatography using ethyl acetate/hexane (1:3); yield: 82 %; **^{1}H NMR** (500 MHz, DMSO-d6, 25 °C): δ = 7.94 (d, $J = 8.68$ Hz, 1H, 3-Ph*H*); 7.88 (d, $J = 2.12$ Hz, 1H, 6-Ph*H*); 7.80 (dd, $J = 8.69$; 2.19 Hz, 1H, 4-

Ph*H*); 6.04 (q, *J* = 6.53 Hz, 1H, C*H*OR); 2.03 (s, 3H, CH_3COOR); 1.57 (d, *J* = 6.56 Hz, 3H, CHORCH_3); **^{13}C NMR** (126 MHz, DMSO-d6, 25 °C): δ = 169,8 (CH$_3$*C*OOR); 146.6 (2-*Ph*NO$_2$); 138.9 (1- *Ph*CHOHCH$_3$); 132.1 (4-*Ph*H); 130.3 (6-*Ph*H); 127,7 (5-*Ph*Br); 126.4 (3-*Ph*H); 67.0 (1-Ph*C*HORCH$_3$); 56.2 (O*C*H$_3$); 21.2 (1-PhCHOH*C*H$_3$); 20.7 (*C*H$_3$COOR); **HRMS** (ESI, m/z) calcd for $C_8H_7NO_2{}^{81}Br$ [M-CH$_3$COO]$^+$: 229.9635, found: 229.9631; **IR**: 696 (w), 758 (m), 832 (m), 867 (m), 891 (w), 943 (m), 1025 (m), 1059 (m), 1075 (m), 1096 (m), 1150 (w), 1188 (m), 1230 (vs), 1294 (m), 1351 (s), 1371 (m), 1451 (m), 1523 (vs), 1566 (m), 1602 (m), 1742 (s), 2869 (w), 2936 (w), 2982 (w), 3102 (w) cm^{-1}.

1-(5-azido-2-nitrophenyl)ethyl acetate (**8e**)

Starting material: **6e** (1.6 mmol); purification: flash column chromatography using ethyl acetate/hexane (1:3); yield: 62 %; **^{1}H NMR** (500 MHz, DMSO-d6, 25 °C): δ = 8.01 (d, *J* = 8.40 Hz, 1H, 3-Ph*H*); 7.69 (d, *J* = 1.84 Hz, 1H, 6-Ph*H*); 7.55 (dd, *J* = 8.39; 1.89 Hz, 1H, 4-Ph*H*); 6.08 (q, *J* = 6.54 Hz, 1H, C*H*OR); 4.67 (d, *J* = 14.23 Hz, 1H, CH_2N$_3$); 4.63 (d, *J* = 14.23 Hz, 1H, CH_2N$_3$); 2.02 (s, 1H, CH_3); 1.57 (d, *J* = 6.53 Hz, 1H, CH_3); **^{13}C NMR** (126 MHz, DMSO-d6, 25 °C): δ = 169.8 (ROO*C*CH$_3$); 146.9 (2-*Ph*NO$_2$); 142.3 (5-*Ph*CH$_2$N$_3$); 137.3 (1-*Ph*CHORCH$_3$); 128.5 (4-*Ph*H); 126.8 (6-*Ph*H); 124.9 (3-*Ph*H); 67.2 (*C*HORCH$_3$); 52.4 (*C*H$_2$N$_3$); 21.4 (CHOR*C*H$_3$); 20.8 (ROOC*C*H$_3$); ^{15}N NMR (51 MHz, DMSO-d6, 25 °C): δ = 374.0 (*N*O$_2$); 248.2 (C-N=*N*=N); 74.2 (C-*N*=N=N); **HRMS** (ESI, m/z): calcd for $C_9H_9N_4O_2$ [M-OAc]$^+$: 205.0721, found: 205.0716; **IR**: 834 (m), 948 (m), 1013 (m), 1030 (m), 1060 (m), 1082 (m), 1165 (m), 1232 (vs), 1339 (m), 1370 (m), 1437 (m), 1457 (w), 1522 (vs), 1590 (w), 1611 (w), 1739 (s), 2098 (s), 2937 (w), 2984 (w) cm^{-1}.

1-(5-((4-butyl-1H-1,2,3-triazol-1-yl)methyl)-2-nitrophenyl)ethyl acetate **(8f)**

Starting material: **6f** (1.2 mmol); purification: flash column chromatography using ethyl acetate/hexane 1:1; yield: 88 %; **^{1}H NMR** (500 MHz, DMSO-d6, 25 °C): δ = 7.99 (d, *J* = 8.50 Hz, 1H, 3-Ph*H*); 7.98 (s, 1H, 5-1,2,3-triazole*H*); 7.53 (d, *J* = 1.98 Hz, 1H, 6-Ph*H*). 7.35 (dd, *J* = 8.47; 1.91 Hz, 1H, 4-Ph*H*); 6.05 (q, *J* = 6.55 Hz, 1H, C*H*OR); 5.71 (d, *J* = 2.51 Hz, 2H, CH_2N); 2.62 (t, *J* = 7.55 Hz, 2H, $CH_2CH_2CH_2CH_3$); 1.98 (s, 3H, $CHORCH_3$); 1.58 (p, *J* = 7.50 Hz, 2H, $CH_2CH_2CH_2CH_3$); 1.53 (d, *J* = 6.59 Hz, 3H, CH_3); 1.31 (m, 2H, $CH_2CH_2CH_2CH_3$); 0.88 (t, *J* = 7.35 Hz, 3H, $CH_2CH_2CH_2CH_3$); **^{13}C NMR** (126 MHz, DMSO-d6, 25 °C): δ = 169,6 (CH3*C*OOR); 147.4 (4-1,2,3-triazole); 146.8 (2-*Ph*NO_2); 142.7 (5-*Ph*CH_2N); 137.3 (1-*Ph*$CHORCH_3$); 127.8 (4-*Ph*H); 126.1 (6-*Ph*H); 124.9 (3-*Ph*H); 122.4 (5-1,2,3-triazole); 67.1 ($CHORCH_3$); 51.7 (CH_2N); 31.1 ($CH_2CH_2CH_2CH_3$); 24.6 ($CH_2CH_2CH_2CH_3$); 21.6 ($CH_2CH_2CH_2CH_3$); 21.3 ($CHORCH_3$); 20.6 (CH_3COOR); 13.6 ($CH_2CH_2CH_2CH_3$); **^{15}N NMR** (51 MHz, DMSO-d6, 25 °C): δ = 374.2 (NO_2); 362.1 (2-1,2,3-triazole); 353.9 (3-1,2,3-triazole); 244.9 (1-1,2,3-triazole); **HRMS** (ESI, m/z) calcd for $C_{17}H_{23}N_4O_4$ $[M+H]^+$: 347.1714, found: 347.1710; **IR**: 670 (w), 702 (w), 746 (m), 790 (m), 810 (m), 838 (m), 852 (m), 935 (w), 953 (w), 993 (m), 1027 (m), 1045 (m), 1073 (m), 1148 (m), 1162 (m), 1218 (s), 1236 (vs), 1296 (m), 1339 (m), 1373 (m), 1429 (w), 1437 (w), 1457 (w), 1507 (m), 1521 (m), 1558 (m), 1616 (m), 1686 (w), 1718 (s), 1742 (m), 2855 (w), 2871 (w), 2928 (w), 2954 (w), 3157 (vw) cm^{-1}.

1-(5-acetamido-2-nitrophenyl)ethyl acetate (**8h**)

Starting material **6g** (1.3 mmol); purification: flash column chromatography using methanol/dichloromethane 1:20; yield: 63 %; **^{1}H NMR** (500 MHz, $CDCl_3$ 25 °C): δ = 7.91 (d, *J* = 8.40 Hz, 1H, 3-Ph*H*); 7.51 (d, *J* = 1.93 Hz, 1H, 6-Ph*H*); 7.32 (dd, *J* = 8.56; 1.94 Hz, 1H, 4-Ph*H*); 6.31 (q, *J* = 6.47 Hz, 1H, C*H*OR); 5.99 (s, 2H, N*H*); 4.53 (d, *J* = 16.52 Hz, 1H, CH_2N);

4.48 (d, *J* = 16.39 Hz, 1H, C*H*$_2$N); 2.07 (s, 6H, C*H*$_3$COOR, C*H*$_3$CONR); 1.61 (d, *J* = 6.44 Hz, 3H, CHORC*H*$_3$); **^{13}C NMR** (126 MHz, CDCl$_3$, 25 °C): δ = 170.3 (CH$_3$*C*ONR); 170.0 (CH$_3$*C*OOR); 146.8 (2-*Ph*NO$_2$); 144.9 (5-PhCH$_2$N); 138.7 (4-*Ph*CHORCH$_3$); 127.4 (4-*Ph*H); 126.3 (6-*Ph*H); 125.2 (3-*Ph*H); 68.2 (*C*HORCH$_3$); 43.1 (*C*H$_2$N); 23.3 (*C*H$_3$); 22.1 (CHOR*C*H$_3$); 21.2 (*C*H$_3$); **^{15}N NMR** (51 MHz, CDCl$_3$, 25 °C): δ = 372.2 (*N*O$_2$); 115.9 (CH$_3$CO*N*); **HRMS** (ESI, m/z): calcd for $C_{11}H_{13}N_2O_3$ [M-OAc]$^+$: 221.0921, found: 221.0918; **IR**: 837 (m), 906 (w), 947 (m), 1033 (m), 1059 (m), 1080 (m), 1165 (m), 1233 (s), 1286 (m), 1339 (m), 1370 (m), 1419 (m), 1437 (m), 1456 (m), 1519 (vs), 1590 (m), 1610 (m), 1653 (m), 1739 (m), 2934 (w), 2984 (w), 3068 (w) cm^{-1}.

1-(5-(N-acetylacetamido)-2-nitrophenyl)ethyl acetate (**8i**)

Starting material **6g** (1.3 mmol); purification: flash column chromatography using methanol/dichloromethane 1:20; yield: 15 %; **^{1}H NMR** (500 MHz, CDCl$_3$, 25 °C): δ = 7.95 (d, *J* = 8.43 Hz, 1H, 3-Ph*H*); 7.40 (d, *J* = 2.06 Hz, 1H, 6-Ph*H*); 7.20 (dd, *J* = 8.55; 2.05 Hz, 1H, 4-Ph*H*); 6.33 (q, *J* = 6.49 Hz, 1H, C*H*OR); 5.05 (d, *J* = 17.11 Hz, 1H, C*H*$_2$N); 4.98 (d, *J* = 17.13 Hz, 1H, C*H*$_2$N); 2.43 (s, 6H, C*H*$_3$CONR); 2.06 (s, 3H, C*H*$_3$COOR); 1.61 (d, *J* = 6.51 Hz, 3H, C*H*$_3$); **^{13}C NMR** (126 MHz, CDCl$_3$, 25 °C): δ = 173.2 (CH$_3$*C*ONR); 169.9 (CH$_3$*C*OOR); 146.8 (2-*Ph*NO$_2$); 143.4 (5-*Ph*CH$_2$N); 139.1 (4-*Ph*CHORCH$_3$); 126.0 (4-*Ph*H); 125.5 (3-*Ph*H); 124.9 (6-*Ph*H); 68.2 (*C*HORCH$_3$); 47.5 (CH$_2$N); 26.6 (*C*H$_3$CONR); 22.1 (CHOR*C*H$_3$); 21.1 (*C*H$_3$COOR); **^{15}N NMR** (51 MHz, CDCl$_3$, 25 °C): δ = 371.3 (*N*O$_2$); 173.1 ((CH$_3$CO)$_2$*N*); **HRMS** (ESI, m/z): calcd for $C_{13}H_{15}N_2O_4$ [M-OAc]$^+$: 263.1027found: 263.1024; **IR**: 839 (m), 875 (w), 908 (w), 948 (m), 977 (m), 1029 (m), 1059 (m), 1082 (m), 1164 (m), 1204 (vs), 1233 (s), 1262 (m), 1334 (m), 1368 (s), 1419 (m), 1521 (s), 1590 (w), 1611 (w), 1701 (s), 1739 (m), 2938 (w), 2981 (w) cm^{-1}.

1-(5-((4-butyl-1H-1,2,3-triazol-1-yl)methyl)-2-nitrophenyl)ethyl benzoate (**9f**)

To an oven-dried two-necked round-bottom flask **6f** (171 mg, 0.56 mmol) was added. A stopcock joint and a septum were attached. The flask was sealed and filled with argon before ketylated tetrahydrofuran (20 mL) was added. The reaction mixture was cooled to -45 °C using a cold bath. Freshly titrated n-butyllithium in hexane (0.1 mL, 0.86 mmol) was slowly added dropwise. The reaction mixture was stirred at -45 °C for one hour, and then benzoyl chloride (0.54 mL, 0.68 mmol) was added. The cold bath was removed; the reaction mixture was stirred for another hour before it was quenched with a saturated sodium bicarbonate solution (10 mL). The tetrahydrofuran was removed in vacuo, and the aqueous phase was extracted with ethyl acetate (50 mL). The organic phase was washed with brine (50 ml) and dried over magnesium sulfate. The solvent was removed to afford the crude product as a reddish-orange oil (299 mg). The crude product was further purified by flash column chromatography using ethyl acetate/hexane (1:1). The product (**9f**) was obtained as a yellow oil with a yield of 114 mg (50%). **^{1}H NMR** (500 MHz, DMSO-d6, 25 °C): δ = 8.03 (d, *J* = 8.43 Hz, 1H, 3-Ph*H*); 7.94 (m, 2H, 2,6-PhCOOR); 7.91 (s, 1H, 5-1,2,3-triazole*H*); 7.69 (m, 1H, 4-PhCOOR); 7.67 (d, *J* = 1.91 Hz, 1H, 6-Ph*H*); 7.55 (t, *J* = 7.82 Hz, 2H, 3,5-PhCOOR); 7.39 (dd, *J* = 8.44; 1.94 Hz, 1H, 4-Ph*H*); 6.34 (q, *J* = 6.44 Hz, 1H, C*H*OR); 5.74 (d, *J* = 15.81 Hz, 1H, CH_2N); 5.70 (d, *J* = 15.69 Hz, 1H, CH_2N); 2.54 (m, 2H, CH_2CH$_2$CH$_2$CH$_3$); 1.68 (d, *J* = 6.49 Hz, 3H, CHORCH_3); 1.48 (m, 2H, CH$_2$CH_2CH$_2$CH$_3$); 1.27 (m, 2H, CH$_2$CH$_2$CH_2CH$_3$); 0.85 (t, *J* = 7.35 Hz, 3H, CH$_2$CH$_2$CH$_2$CH_3); **^{13}C NMR** (126 MHz, DMSO-d6, 25 °C): 164.8 (Ph*C*OOR); 147.4 (4-1,2,3-triazole); 146.8 (2-*Ph*NO$_2$); 142.8 (5-*Ph*CH$_2$N); 137.2 (1-*Ph*CHORCH$_3$); 133.6 (4-*Ph*COOR); 129.2 (2,3-*Ph*COOR); 129.2 (1-*Ph*COOR); 128.8 (3,5-*Ph*COOR); 128.0 (4-*Ph*H); 126.2 (6-*Ph*H); 125.0 (3-*Ph*H); 122.3 (5-1,2,3-triazole); 68.1 (*C*HORCH$_3$); 51.6 (*C*H$_2$N); 31.0 (CH$_2$*C*H$_2$CH$_2$CH$_3$); 24.6 (*C*H$_2$CH$_2$CH$_2$CH$_3$); 21.6 (CH$_2$CH$_2$*C*H$_2$CH$_3$); 21.5 (CHOR*C*H$_3$); 13.6 (CH$_2$CH$_2$CH$_2$*C*H$_3$); **^{15}N NMR** (51 MHz, DMSO-d6, 25 °C): δ = 374.0 (*N*O$_2$); 362.1 (2-1,2,3-

triazole); 354.2 (3-1,2,3-triazole); 245.2 (1-1,2,3-triazole); **HRMS** (ESI, m/z): calcd for $C_{22}H_{25}N_4O_4$ $[M+H]^+$: 409.1871, found: 409.1865; **IR**: 838 (m), 854 (w), 875 (w), 915 (w), 1024 (m), 1048 (m), 1062 (m), 1080 (m), 1107 (m), 1173 (m), 1216 (m), 1272 (vs), 1315 (m), 1330 (m), 1354 (m), 1450 (m), 1522 (s), 1591 (w), 1700 (m), 2858 (w), 2871 (w), 2927 (w), 2956 (w) cm^{-1}.

1-(5-((4-butyl-1H-1,2,3-triazol-1-yl)methyl)-2-nitrophenyl)ethyl phenylcarbamate (**10f**)

To an oven-dried two-necked round-bottom flask **6f** (204 mg, 0.67 mmol), ketylated tetrahydrofuran (25 mL), triethylamine (3 eq) and phenyl isocyanate (1.5 eq). The flask was equipped with an oven-dried reflux condenser, a drying tube loaded with calcium chloride, and a stopper. The reaction mixture was stirred under reflux for 24 h. The reaction was monitored by thin-layer chromatography. Additional triethylamine (3 eq) and phenyl isocyanate (1.5 eq) were added. The reaction mixture was stirred for a further 65 h at room temperature. The solvent was in vacuo. Purification was carried out by column chromatography using ethyl acetate/hexane (1:1). The product (**10f**) was obtained as a colorless solid with a yield of 132 mg (34%) and a purity of 88 %. **^{1}H NMR** (500 MHz, DMSO-d6, 25 °C): δ = 8.01 (d, J = 8.39 Hz, 1H, 3-Ph*H*); 7.90 (s, 1H, 5-1,2,3-triazole*H*); 7.60 (d, J = 2.12 Hz, 1H, 6-Ph*H*); 7.37 (m, 3H, 2,4,6-PhNHCOOR); 7.24 (t, 2H, 3,5-PhNHCOOR); 6.54 (m, 1H, 4-Ph*H*); 5.71 (d, J = 16.11 Hz, 1H, C*H*2N); 5.70 (d, J = 16.11 Hz, 1H, C*H*2N); 4.98 (s, 1H, N*H*); 2.55 (dd, J = 8.38; 6.89 Hz, 2H, CH_2 as C*H*2CH2CH2CH3); 1.59 (d, J = 6.56 Hz, 3H, CHORC*H*3); 1.51 (p, J = 7.59 Hz, 2H, CH2C*H*2CH2CH3); 1.27 (h, J = 7.36 Hz, 2H, CH2CH2C*H*2CH3); 0.85 (t, J = 7.34 Hz, 3H, CH2CH2CH2C*H*3); **^{13}C NMR** (126 MHz, DMSO-d6, 25 °C): δ = 152.4 (PhNH*C*OOR); 147.5 (4-1,2,3-triazole); 146.9 (2-*Ph*NO2); 142.6 (5-*Ph*CH2N); 138.7 (1-*Ph*NHCOOR); 138.0 (1-*Ph*CHORCH3); 128.7 (3,5-*Ph*NHCOOR); 127.9 (4-*Ph*NHCOOR); 126.0 (6-*Ph*H); 125.0 (3-

*Ph*H); 122.2 (5-1,2,3-triazole); 118.1 (2,6-*Ph*NHCOOR); 113.8 (4-*Ph*H); 67.4 (*C*HORCH$_3$); 51.8 (*C*H$_2$N); 31.0 (CH$_2$*C*H$_2$CH$_2$CH$_3$); 24.6 (*C*H$_2$CH$_2$CH$_2$CH$_3$); 21.6 (CH$_2$CH$_2$*C*H$_2$CH$_3$); 21.5 (CHOR*C*H$_3$); 13.6 (CH$_2$CH$_2$CH$_2$*C*H$_3$); **^{15}N NMR** (51 MHz, DMSO-d6, 25 °C): δ = 373.5 (*N*O$_2$); 362.1 (2-1,2,3-triazole); 353.7 (3-1,2,3-triazole); 245.2 (1-1,2,3-triazole); **HRMS** (ESI, m/z): calcd for $C_{22}H_{26}N_5O_4$ $[M+H]^+$: 424.1980, found: 424.1972; **IR**: 841 (m), 893 (w), 991 (m), 1006 (m), 1019 (m), 1043 (m), 1056 (m), 1080 (m), 1122 (m), 1167 (m), 1233 (s), 1270 (s), 1298 (m), 1342 (m), 1448 (m), 1490 (m), 1522 (vs), 1542 (m), 1594 (m), 1697 (m), 1724 (s), 2860 (w), 2929 (w), 2956 (w) cm^{-1}.

2. Results Kinetic Fitting

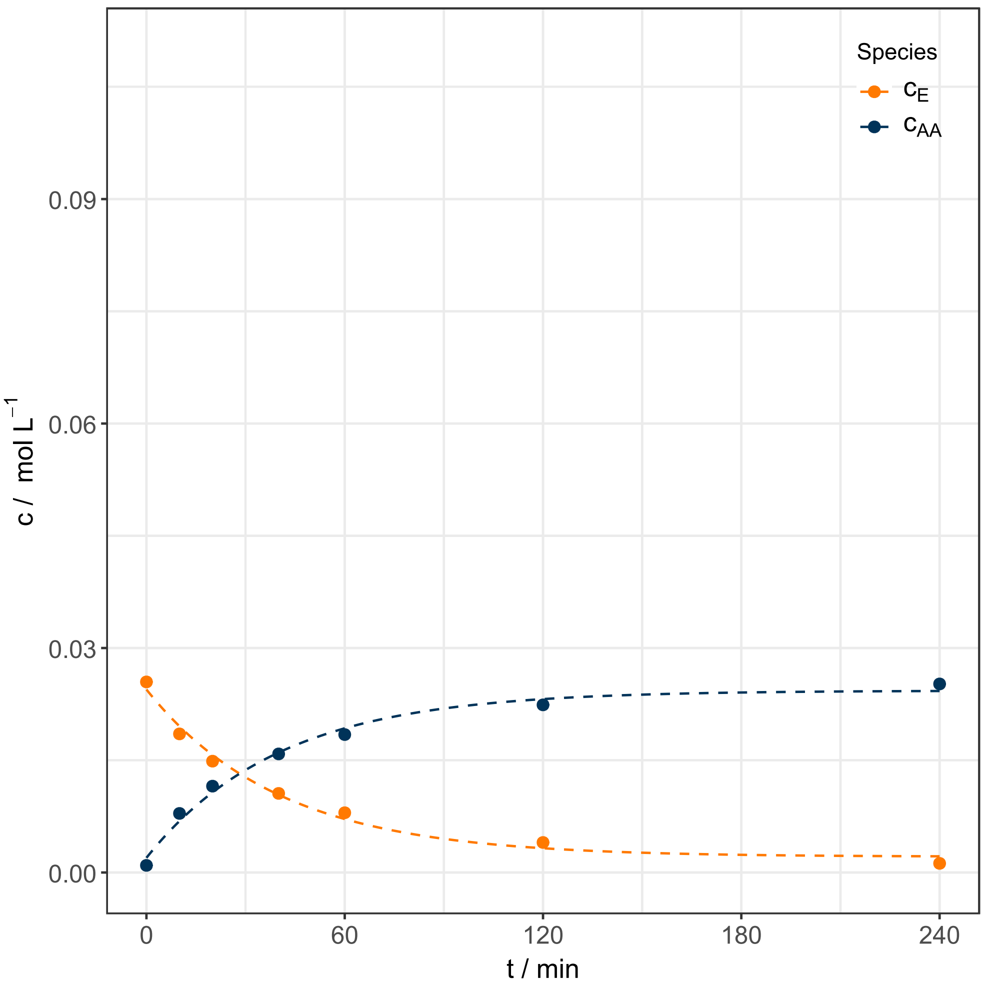

Figure S1. Concentration-time profiles for **7e** (educt) and 4-fluoro-benzoate (acid anion) during the photochemical decomposition using a HgXe-lamp and a starting concentration of 25 mmol L^{-1} in DMSO-d_6 as determined by ^1H NMR spectroscopy.

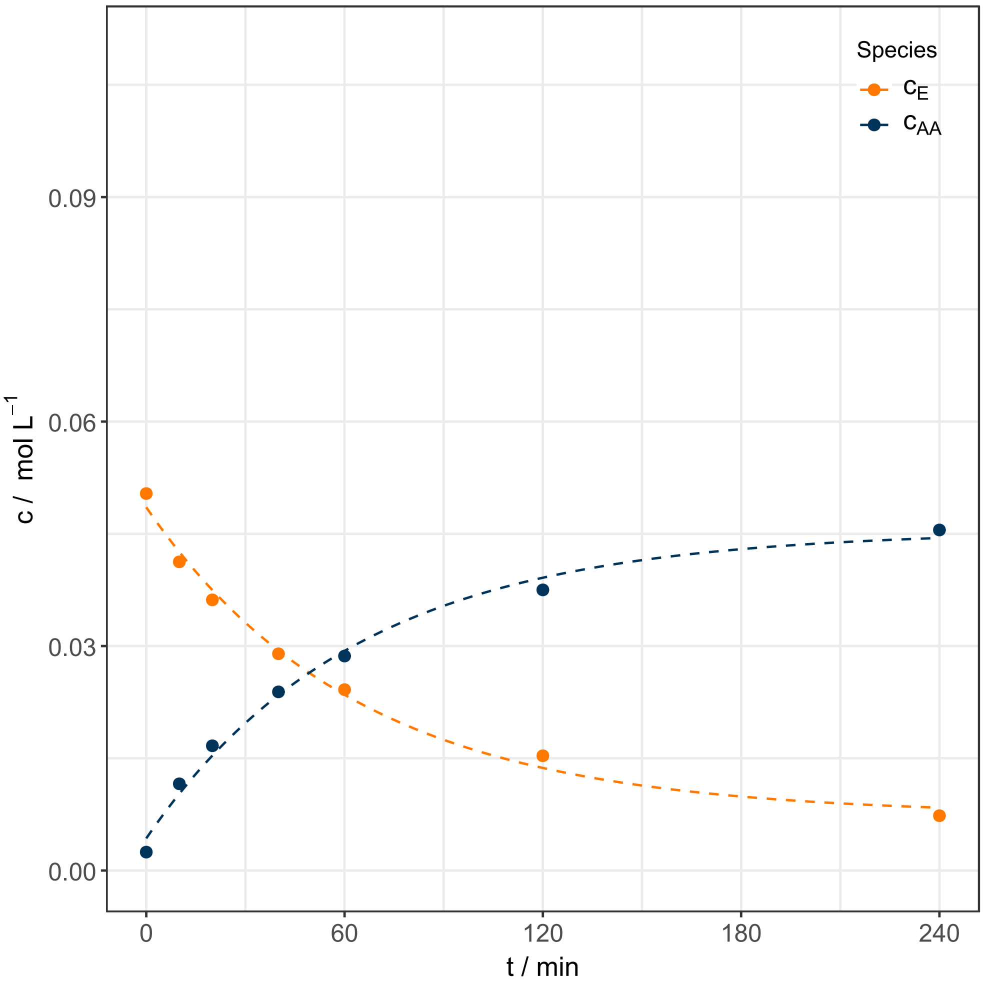

Figure S2. Concentration-time profiles for **7e** (educt) and 4-fluoro-benzoate (acid anion) during the photochemical decomposition using a HgXe-lamp and a starting concentration of 50 mmol L^{-1} in DMSO-d_6 as determined by ^1H NMR spectroscopy.

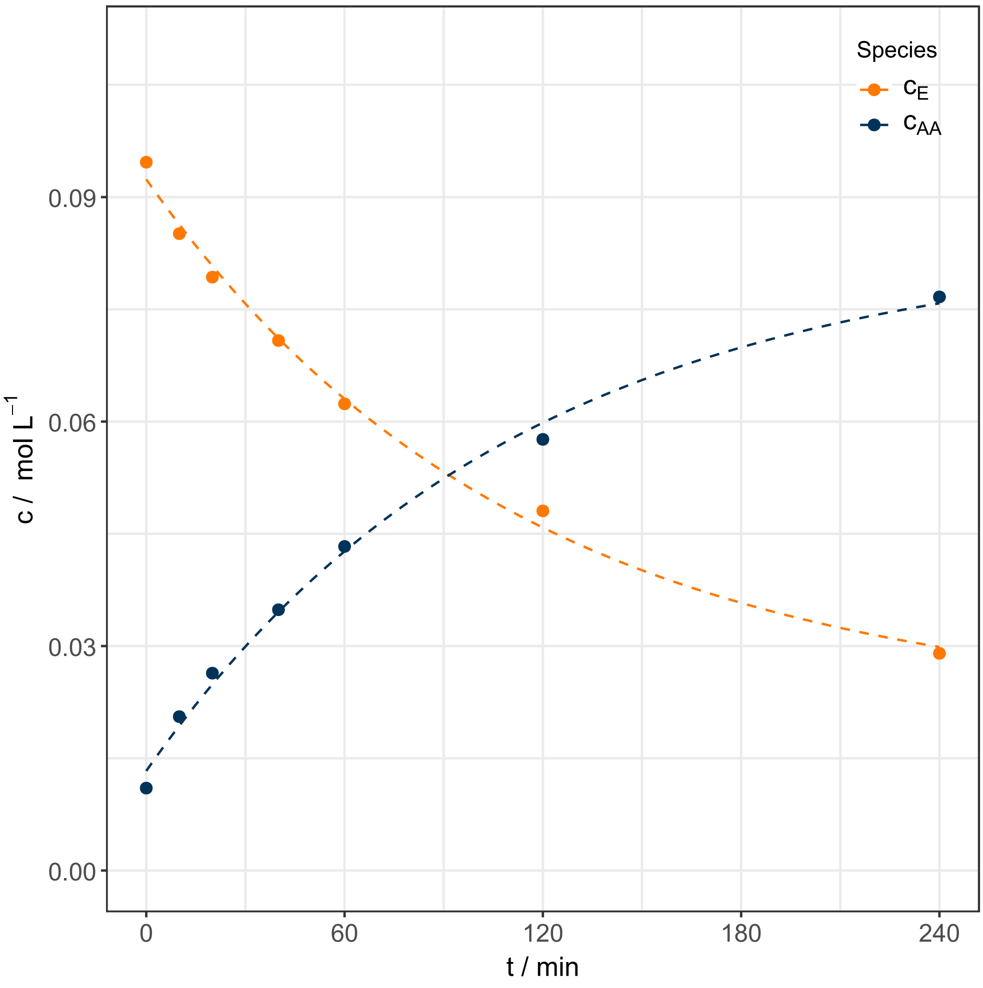

Figure S3. Concentration-time profiles for **7e** (educt) and 4-fluoro-benzoate (acid anion) during the photochemical decomposition using a HgXe-lamp and a starting concentration of 95 mmol L^{-1} in DMSO-d_6 as determined by ^1H NMR spectroscopy.

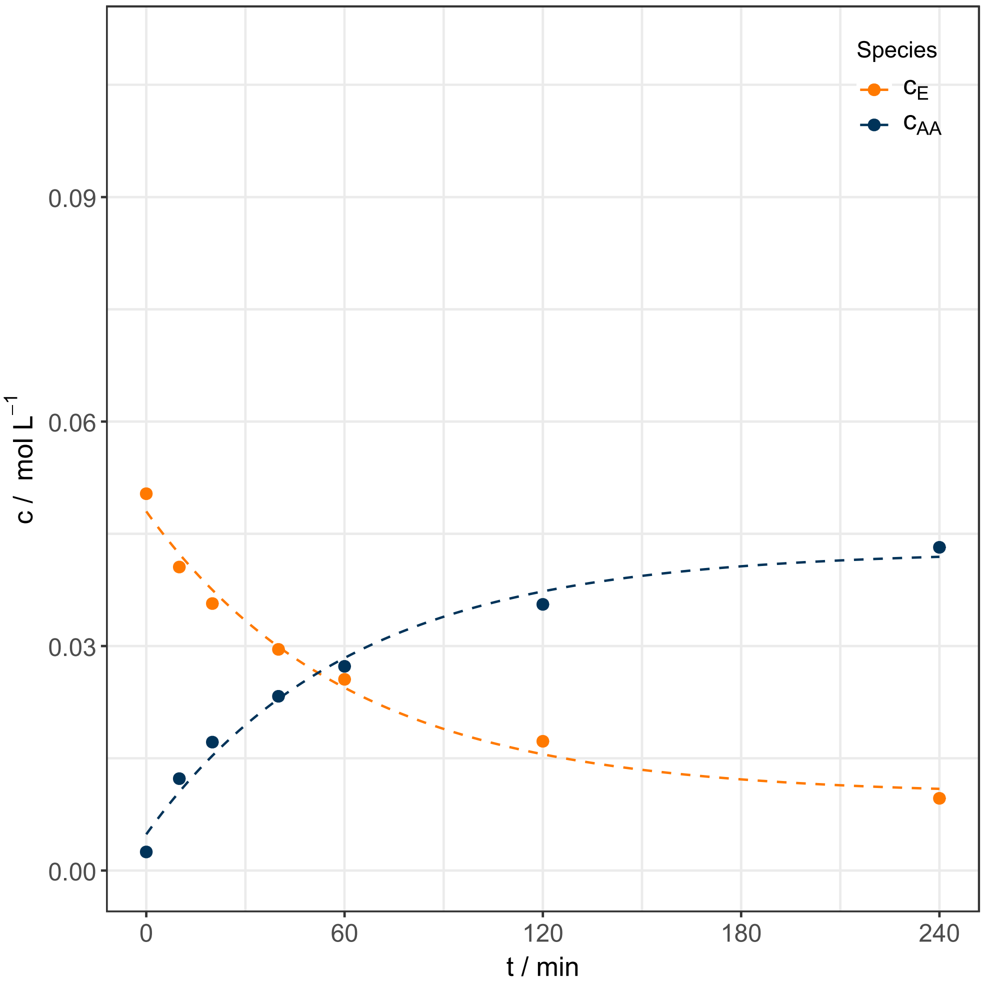

Figure S4. Concentration-time profiles for **7e** (educt) and 4-fluoro-benzoate (acid anion) during the photochemical decomposition using a HgXe-lamp *through window glass* and a starting concentration of 50 mmol L^{-1} in DMSO-d_6 as determined by ^{1}H NMR spectroscopy.

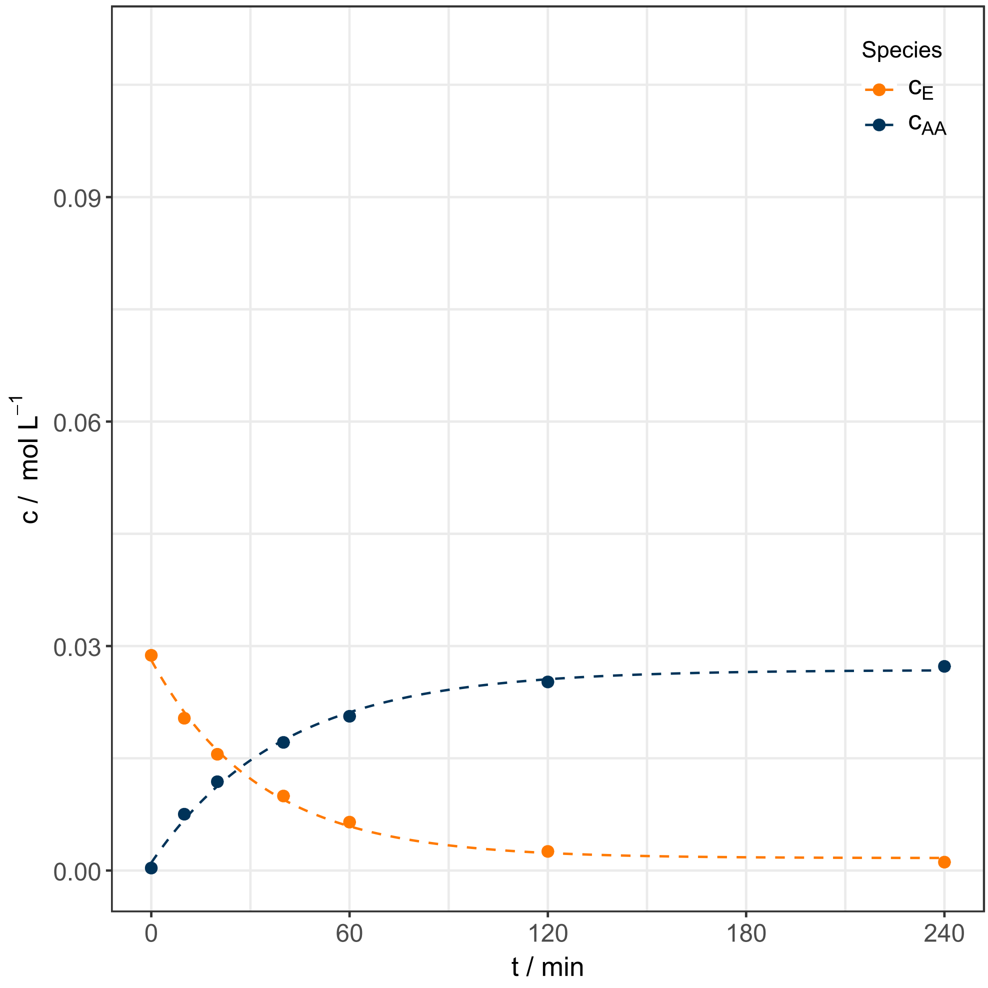

Figure S5. Concentration-time profiles for **8a** (educt) and acetate (acid anion) during the photochemical decomposition using a HgXe-lamp and a starting concentration of 29 mmol L^{-1} in DMSO-d_6 as determined by ^1H NMR spectroscopy.

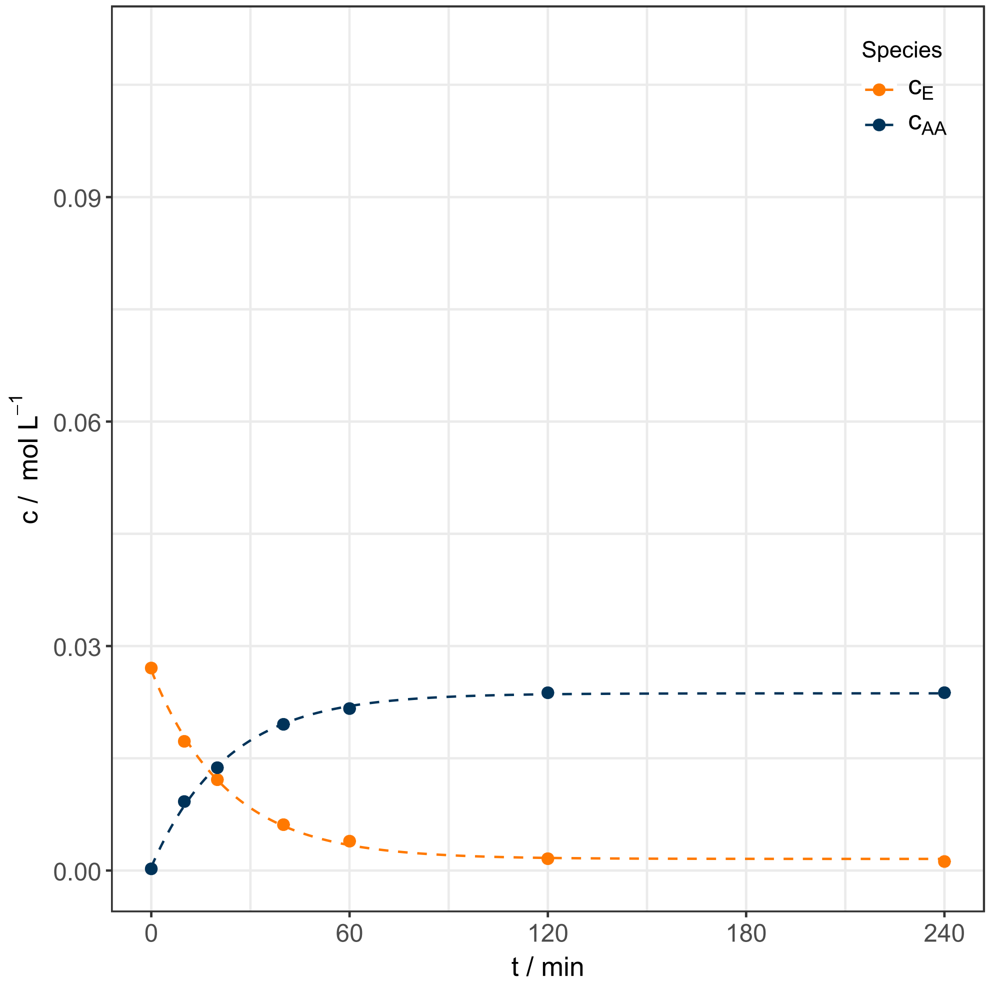

Figure S6. Concentration-time profiles for **8b** (educt) and acetate (acid anion) during the photochemical decomposition using a HgXe-lamp and a starting concentration of 27 mmol L^{-1} in DMSO-d_6 as determined by ^{1}H NMR spectroscopy.

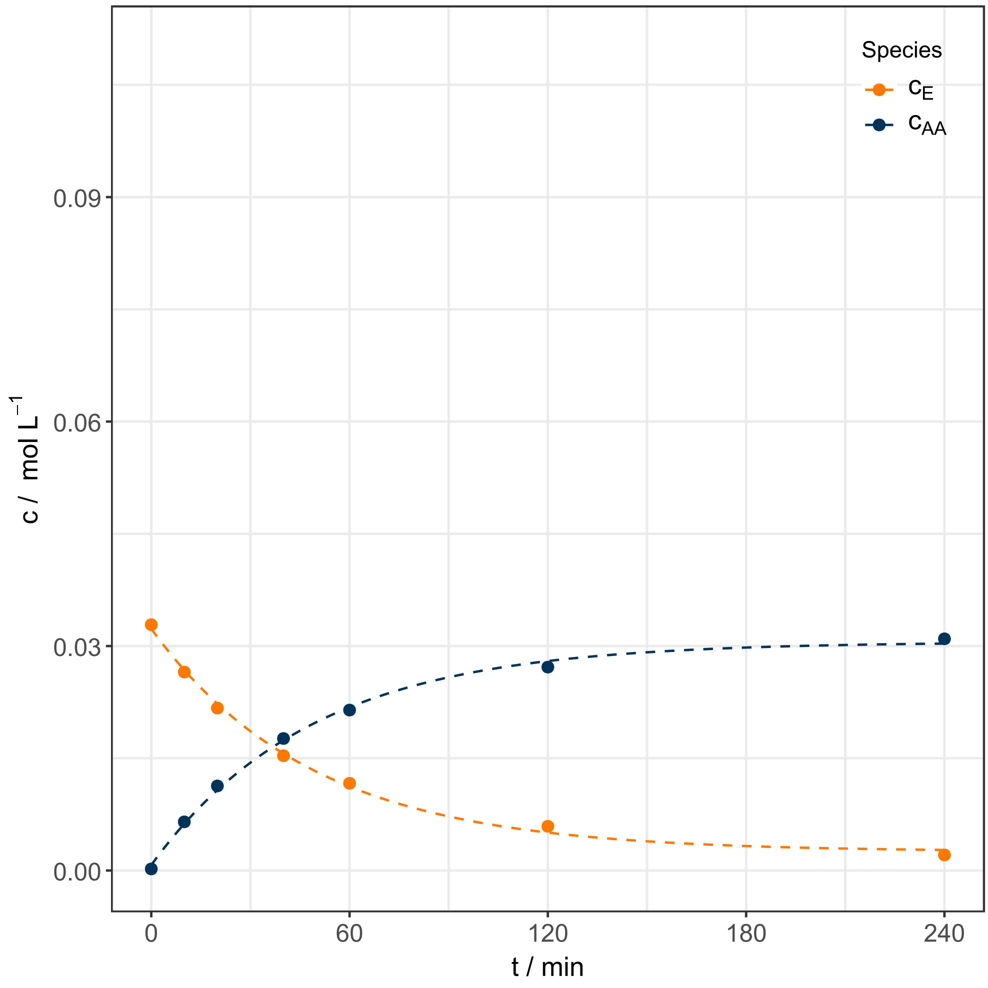

Figure S7. Concentration-time profiles for **8c** (educt) and acetate (acid anion) during the photochemical decomposition using a HgXe-lamp and a starting concentration of 27 mmol L^{-1} in DMSO-d_6 as determined by ^1H NMR spectroscopy.

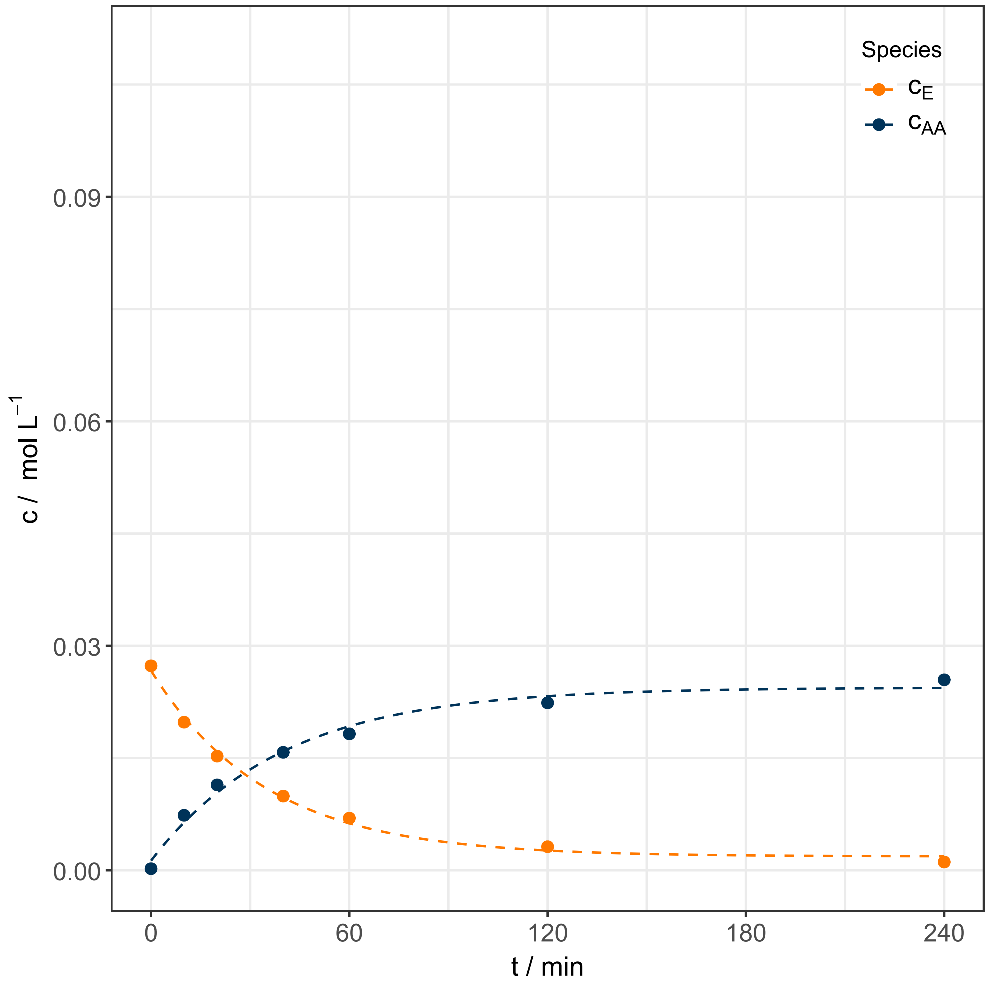

Figure S8. Concentration-time profiles for **8d** (educt) and acetate (acid anion) during the photochemical decomposition using a HgXe-lamp and a starting concentration of 27 mmol L^{-1} in DMSO-d_6 as determined by ^{1}H NMR spectroscopy.

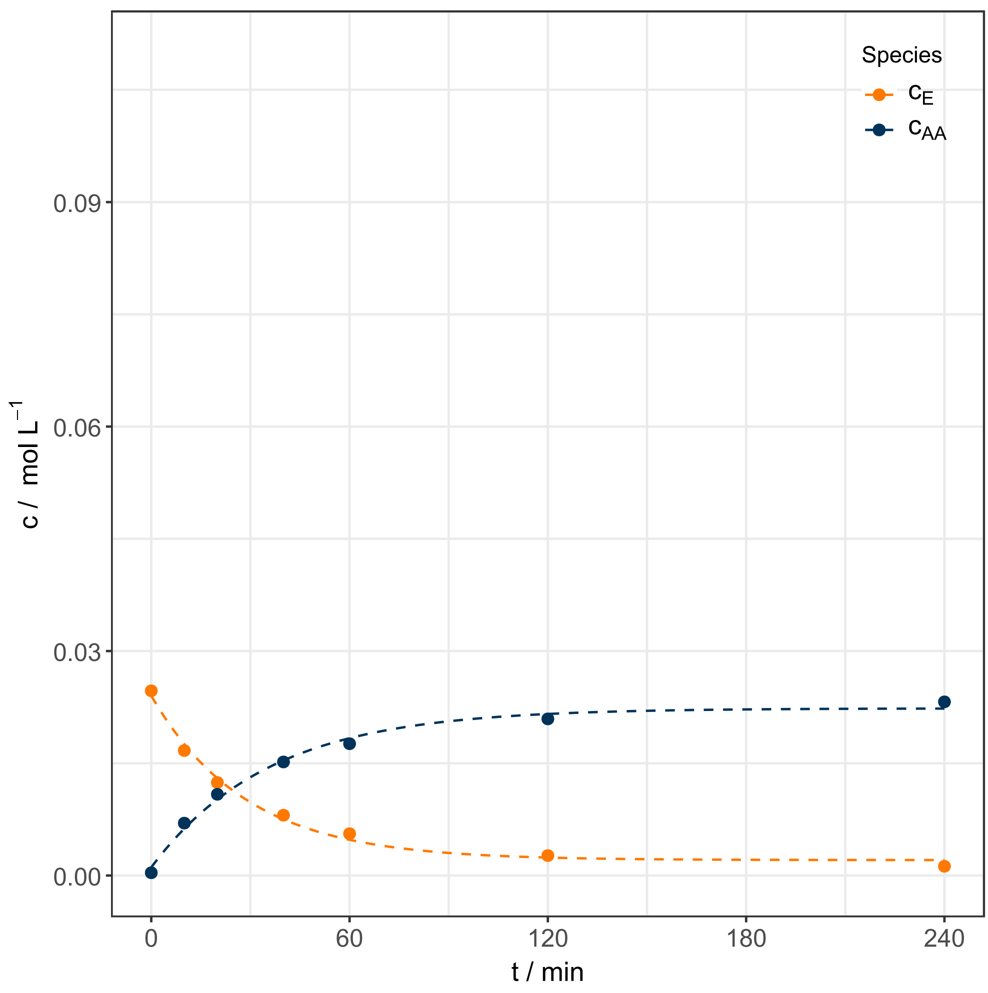

Figure S9. Concentration-time profiles for **8e** (educt) and acetate (acid anion) during the photochemical decomposition using a HgXe-lamp and a starting concentration of 25 mmol L^{-1} in DMSO-d$_6$ as determined by ^{1}H NMR spectroscopy.

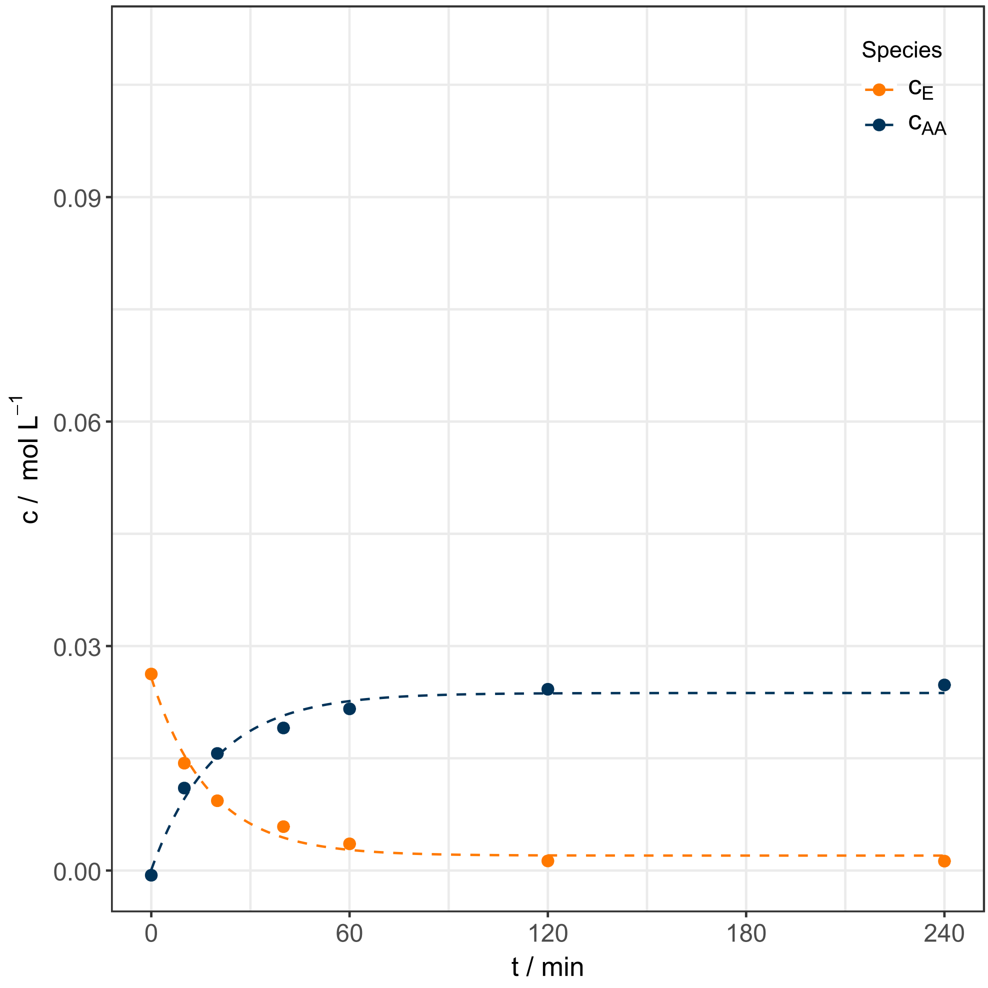

Figure S10. Concentration-time profiles for **8h** (educt) and acetate (acid anion) during the photochemical decomposition using a HgXe-lamp and a starting concentration of 26 mmol L^{-1} in DMSO-d_6 as determined by ^{1}H NMR spectroscopy.

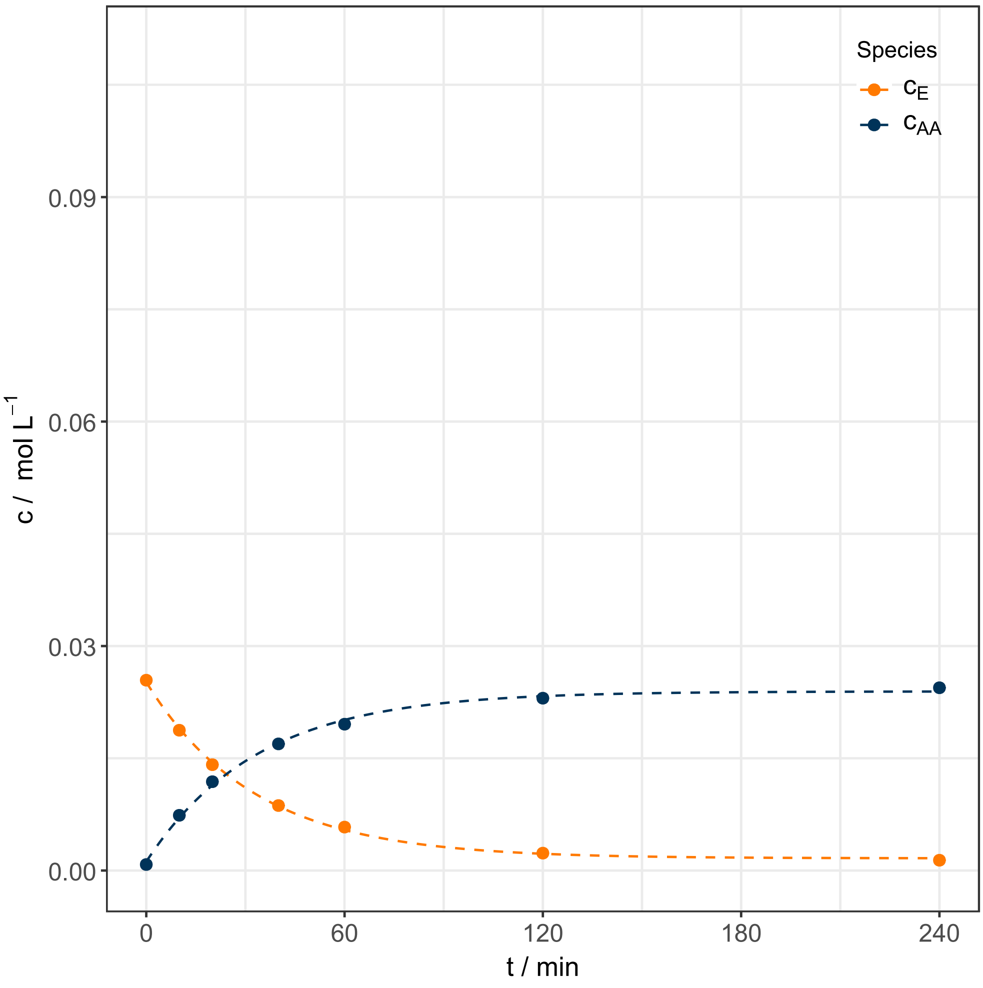

Figure S11. Concentration-time profiles for **8i** (educt) and acetate (acid anion) during the photochemical decomposition using a HgXe-lamp and a starting concentration of 25 mmol L^{-1} in DMSO-d_6 as determined by ^{1}H NMR spectroscopy.

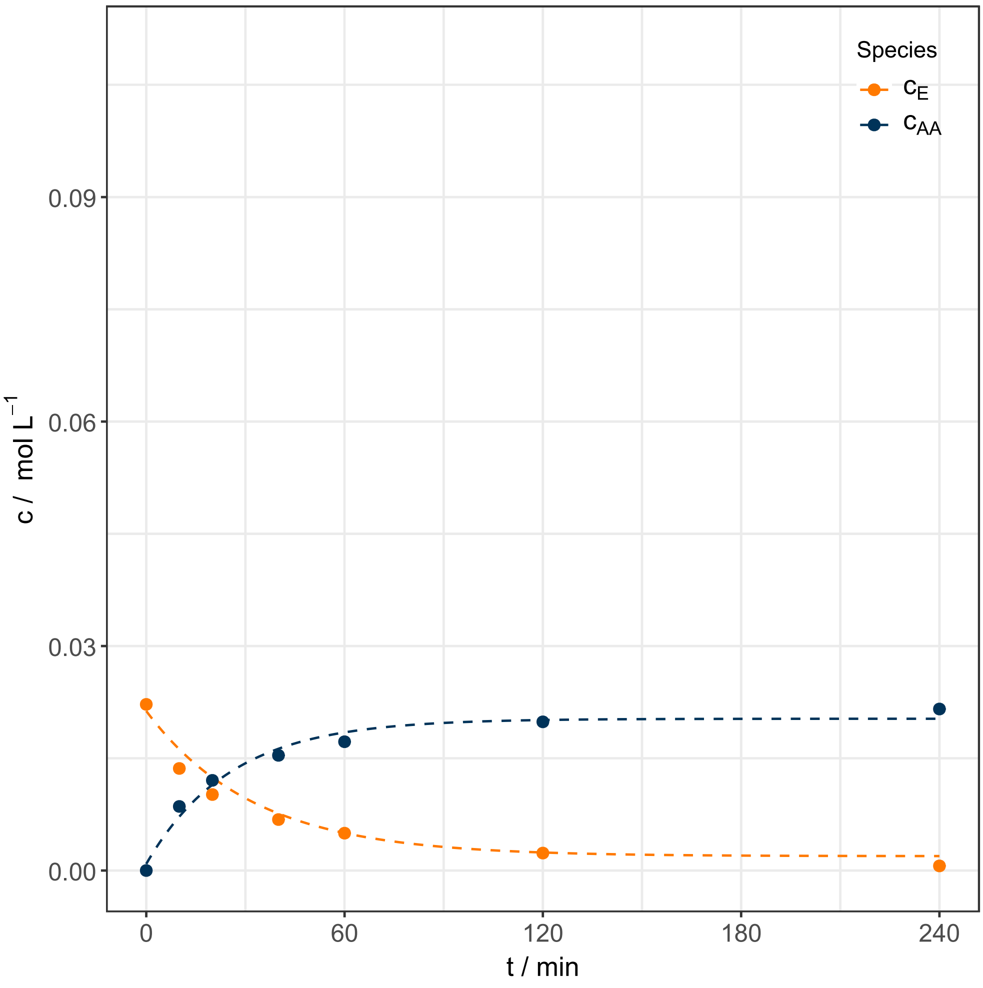

Figure S12. Concentration-time profiles for **7f** (educt) and 4-fluoro-benzoate (acid anion) during the photochemical decomposition using a HgXe-lamp and a starting concentration of 27 mmol L^{-1} in DMSO-d_6 as determined by ^{1}H NMR spectroscopy.

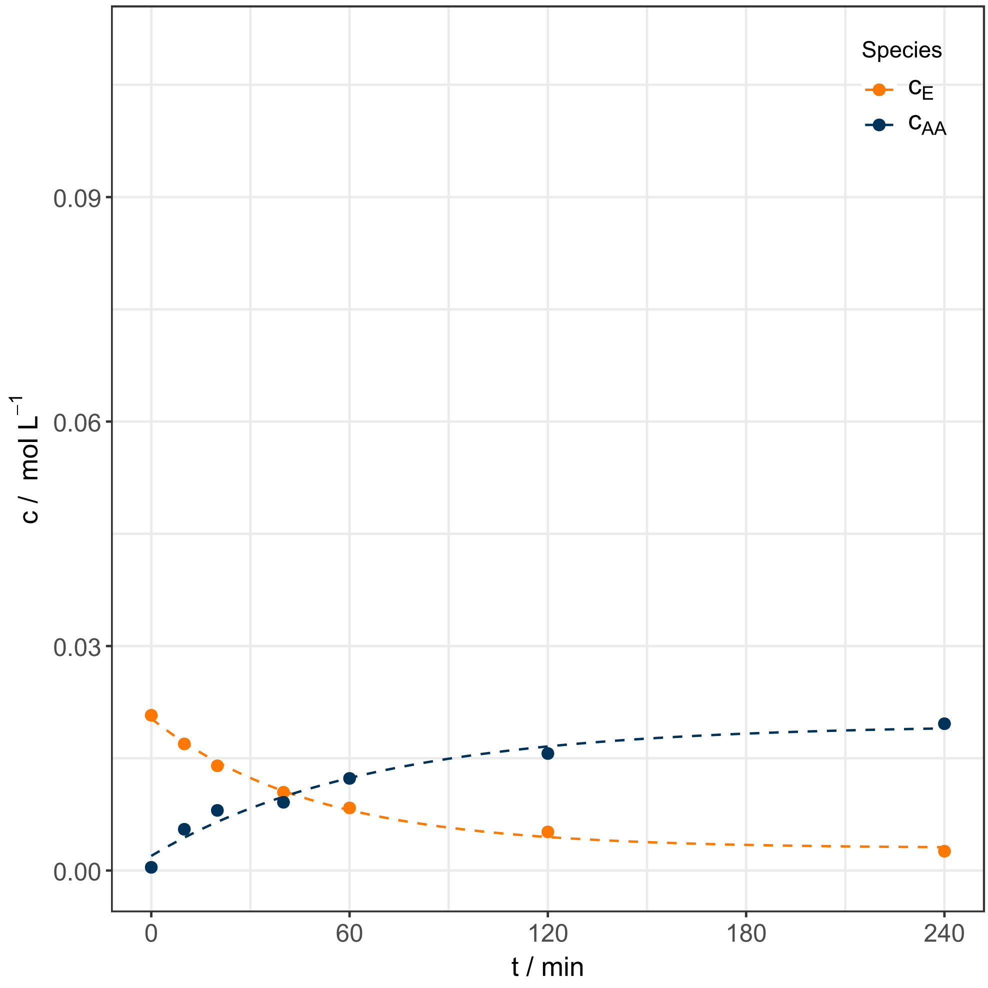

Figure S13. Concentration-time profiles for **8f** (educt) and acetate (acid anion) during the photochemical decomposition using a HgXe-lamp and a starting concentration of 21 mmol L^{-1} in DMSO-d_6 as determined by ^{1}H NMR spectroscopy.

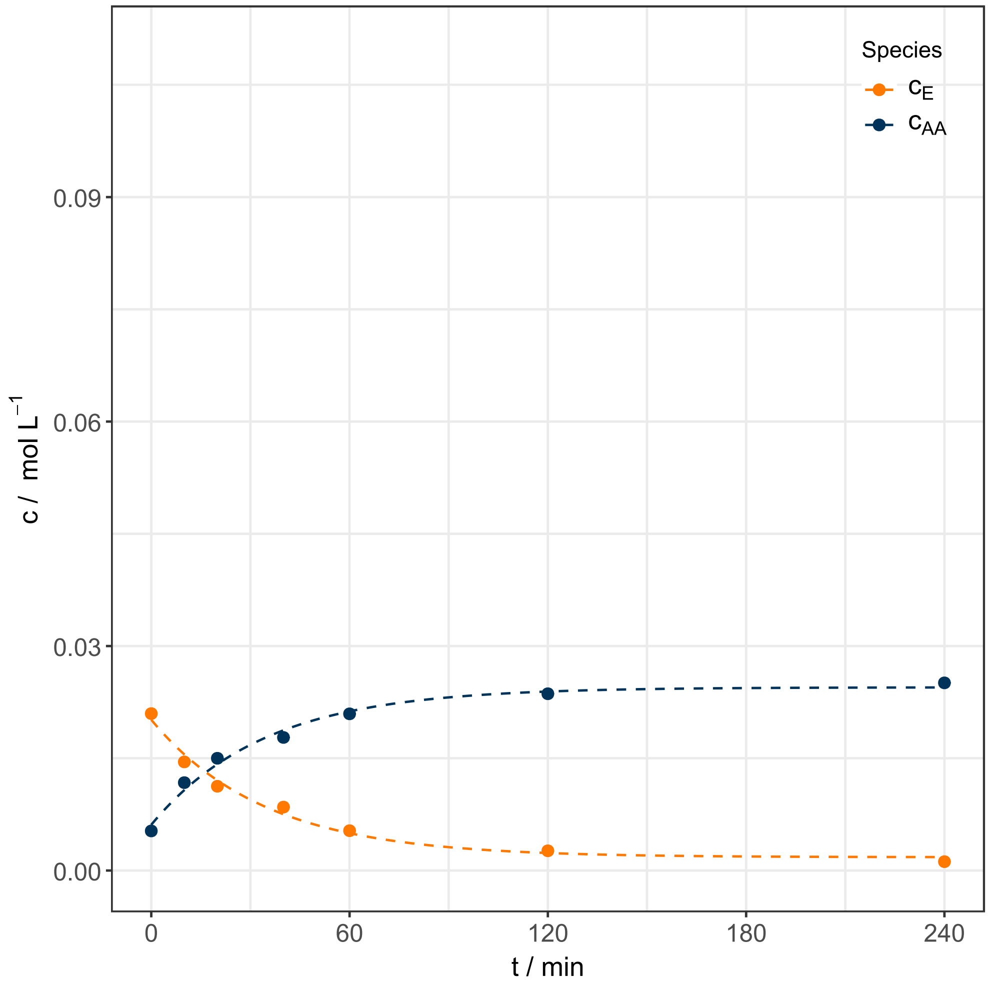

Figure S14. Concentration-time profiles for **9f** (educt) and benzoate (acid anion) during the photochemical decomposition using a HgXe-lamp and a starting concentration of 21 mmol L^{-1} in DMSO-d_6 as determined by ^{1}H NMR spectroscopy.

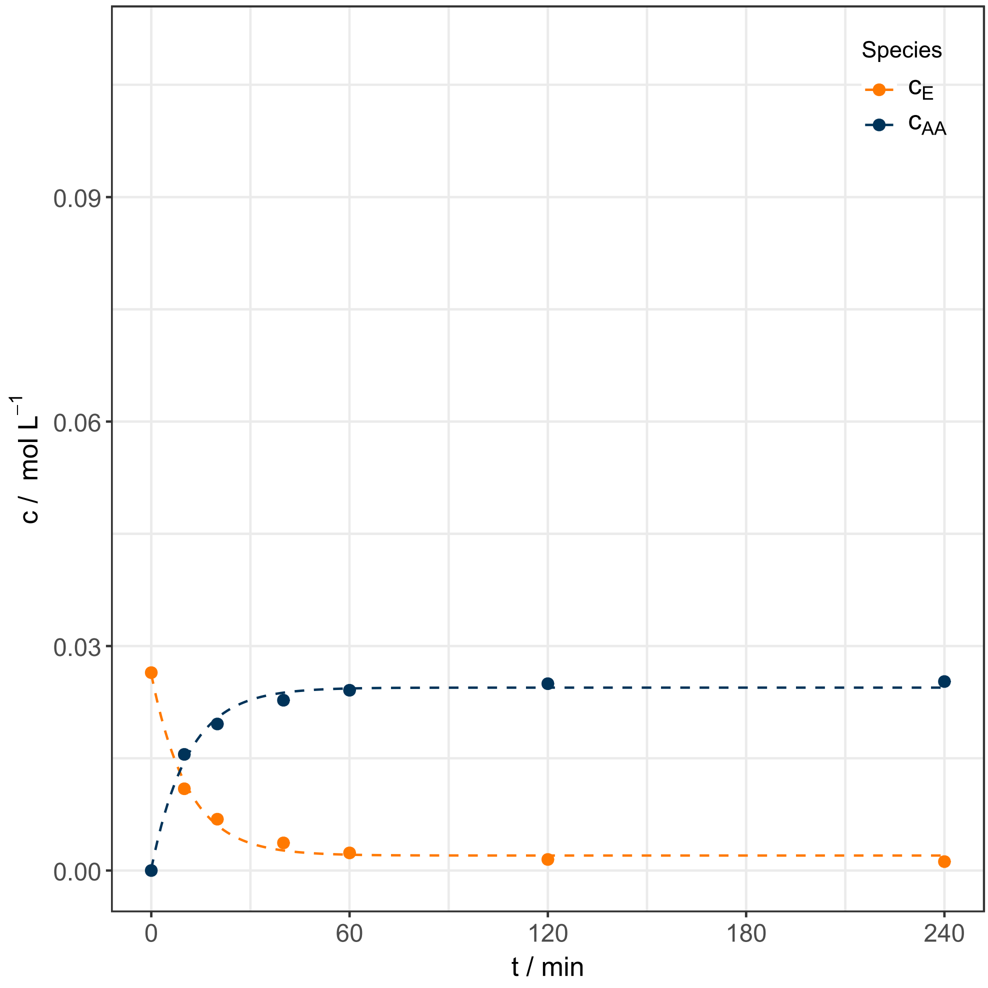

Figure S15. Concentration-time profiles for **10f** (educt) and benzoate (acid anion after decarboxylation) during the photochemical decomposition using a HgXe-lamp and a starting concentration of 26 mmol L^{-1} in DMSO-d_6 as determined by ^1H NMR spectroscopy.

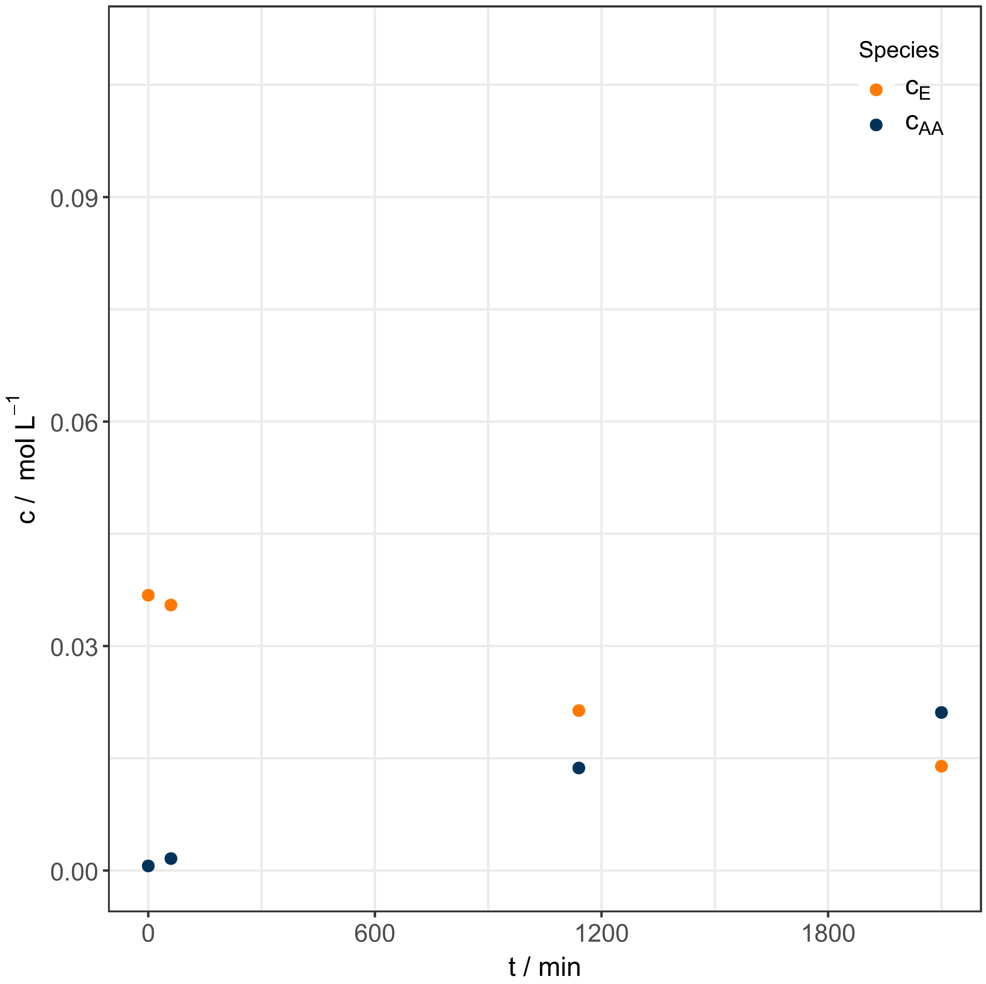

Figure S16. Concentration-time profiles for **8e** (educt) and acetate (acid anion) during the photochemical decomposition using a 275 nm LED and a starting concentration of 37 mmol L^{-1} in DMSO-d_6 as determined by ^{1}H NMR spectroscopy.

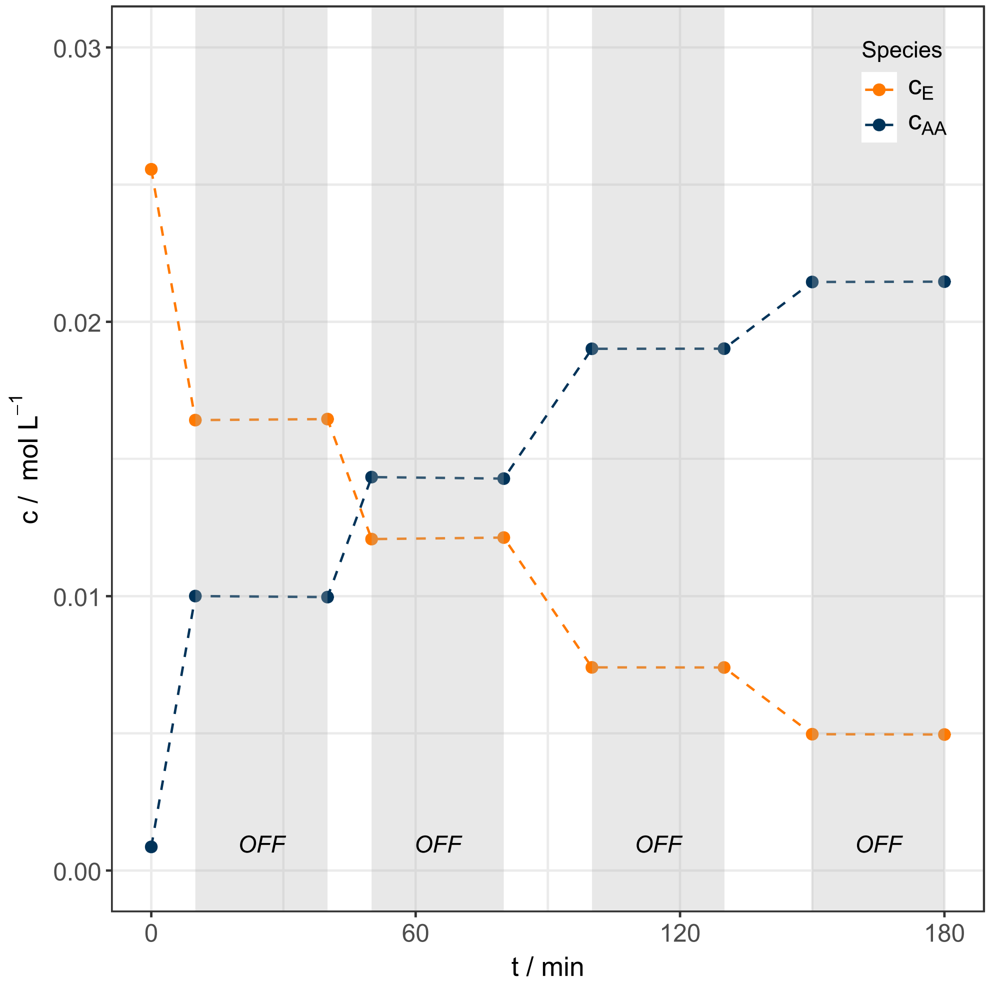

Figure S17. Concentration-time profiles for **7e** (educt) and acetate (acid anion) during the photochemical decomposition using a HgXe-lamp and a starting concentration of 26 mmol L^{-1} in DMSO-d_6 as determined by ^{1}H NMR spectroscopy. The experiment was performed as an ON/OFF study in which after each irradiation phase (ON) and an additional 30 min waiting time without irradiation (OFF) was introduced. At the end of each phase an ^{1}H NMR spectrum was recorded. As can be seen the decomposition is exclusively driven by light.

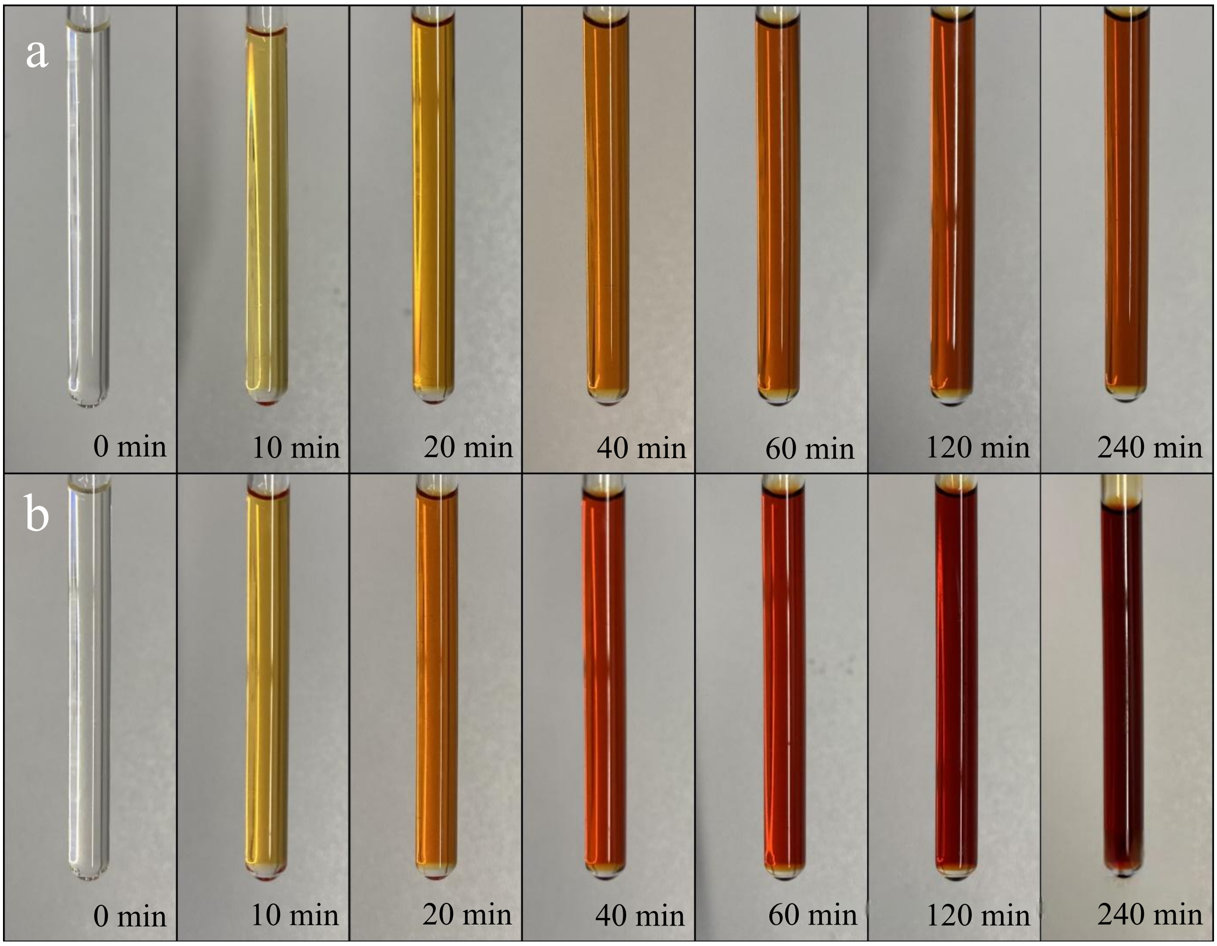

Figure S18. Images of the samples of **7e** after the respective irradiation time. For (a) c_0 = 25 mmol L^{-1} a clear discoloration of the solution can be seen and for (b) c_0 = 95 mmol L^{-1} the discoloration is significantly more intense.

3. UV/Vis spectra

Table S1. Extinction coefficients at 365 nm ε(365 nm) for all oNB esters.

Tag	c_0 [mmol L^{-1}]	ε(365 nm) [L mol^{-1} cm^{-1}]
**7e**	0.97	326
**7f**	0.78	327
**8a**	1.59	224
**8b**	1.49	444
**8c**	0.7	1333
**8d**	1.16	384
**8e**	1.26	333
**8f**	0.96	245
**8h**	1.19	372
**8i**	1.03	417
**9f**	0.04	316
**10f**	1.04	264

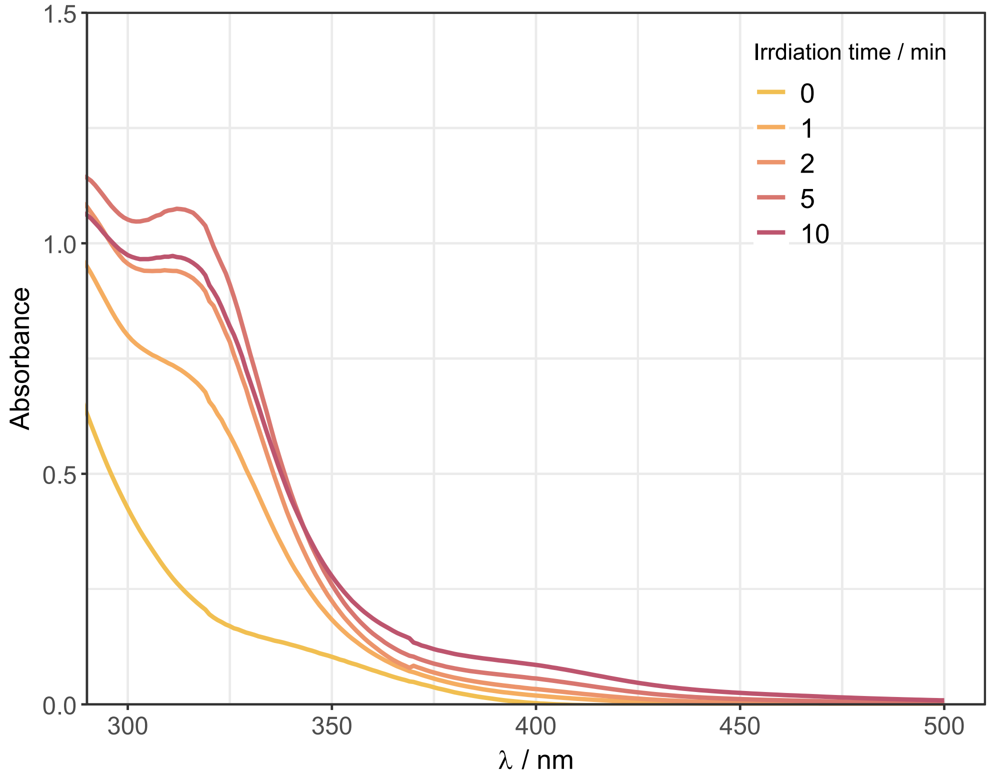

Figure S19. Irradiation time-dependent UV/Vis spectra for the photochemical decomposition of **7e** using a HgXe-lamp and a starting concentration of 0.2 mmol L^{-1} in acetonitrile.

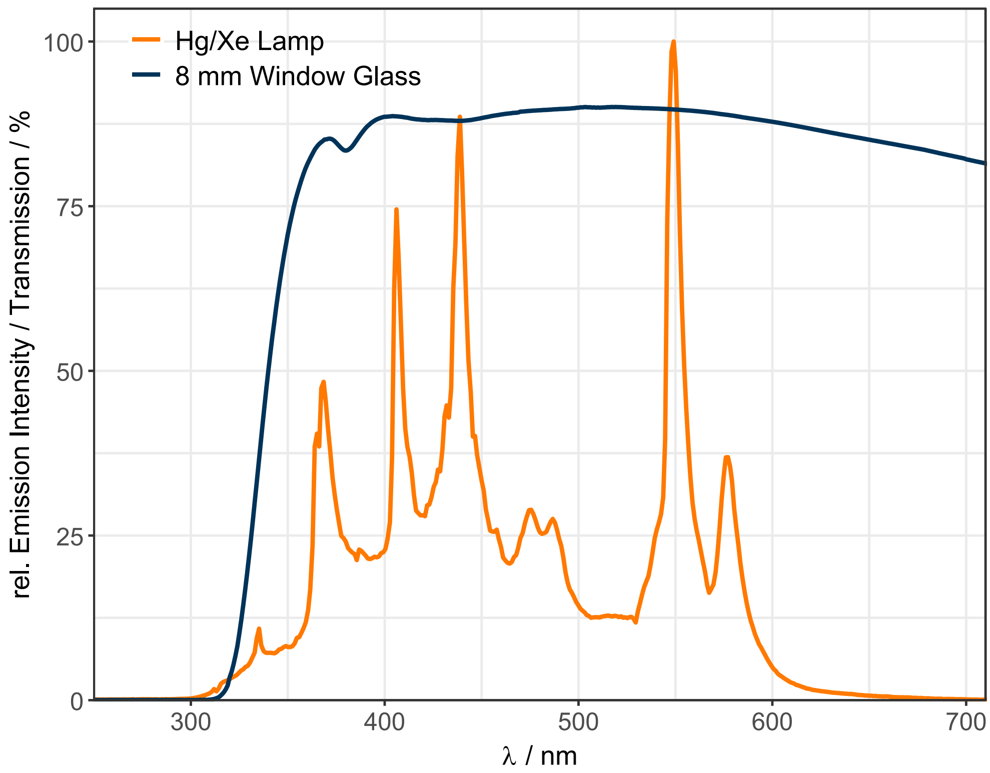

Figure S20. Relative emission spectrum of the utilized Hg/Xe lamp and transmission spectrum of the utilized 8 mm window glass to cut-off UV light during photolysis.

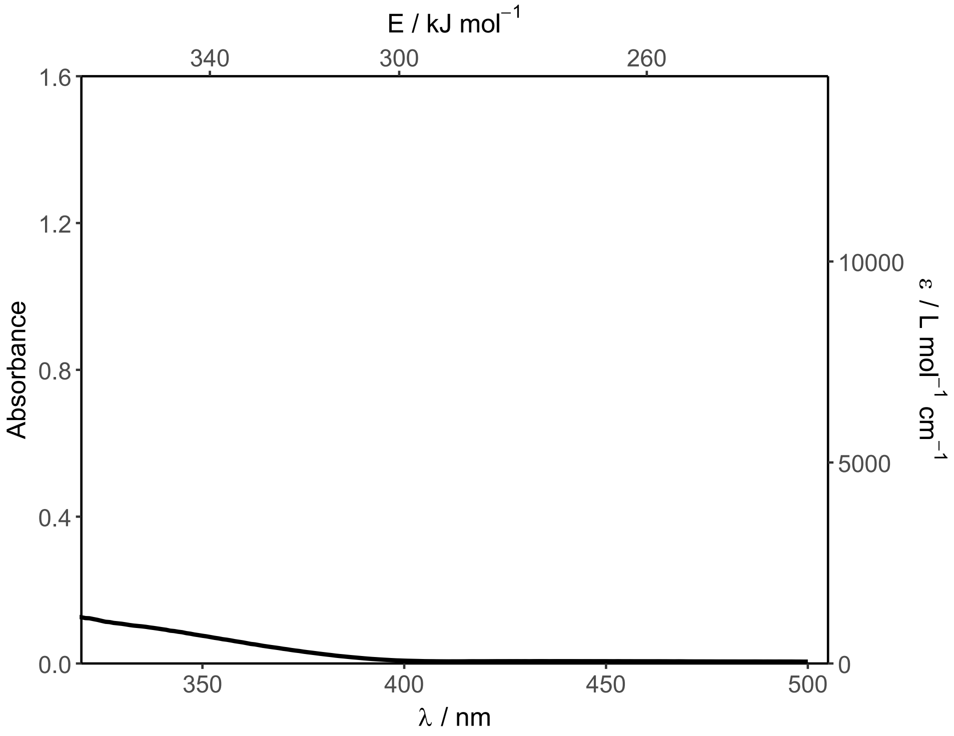

Figure S21. UV/Vis spectrum of triazole alcohol **6f** at 0.11 mmol L^{-1}.

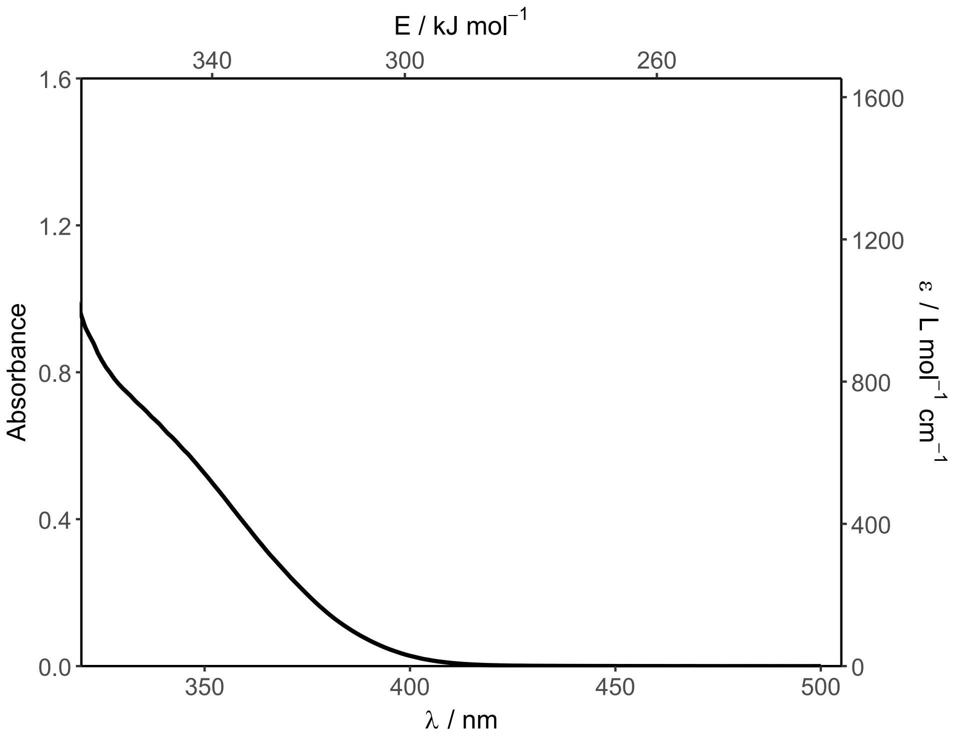

Figure S22. UV/Vis spectrum of azide 4-fluorobenzoate **7e** at 0.97 mmol L^{-1}.

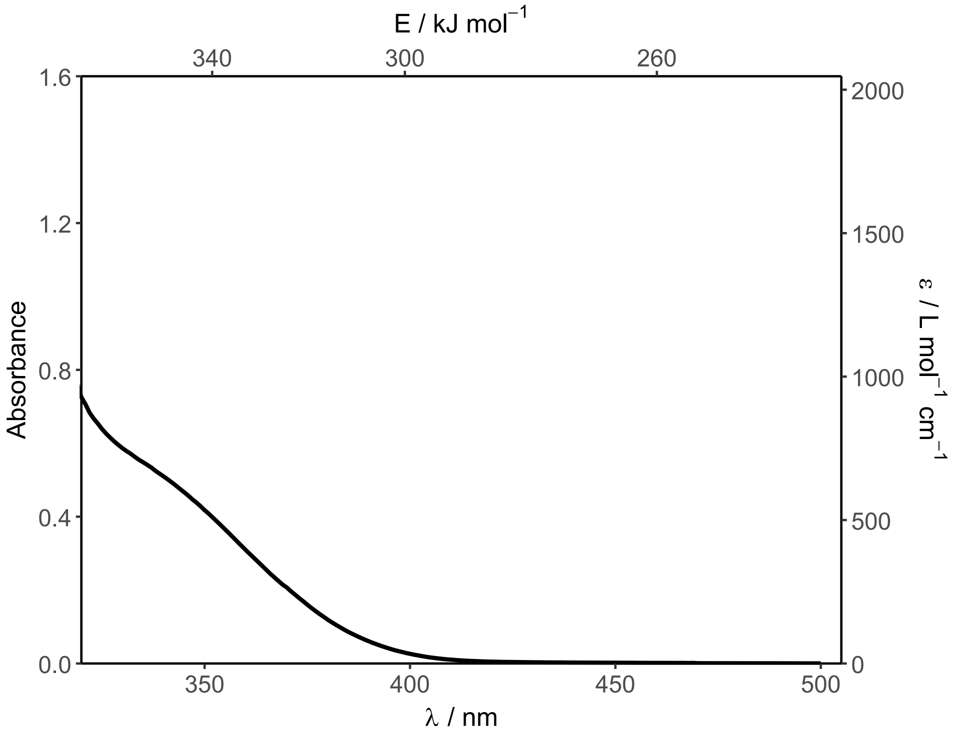

Figure S23. UV/Vis spectrum of triazole 4-fluorobenzoate **7f** at 0.78 mmol L^{-1}.

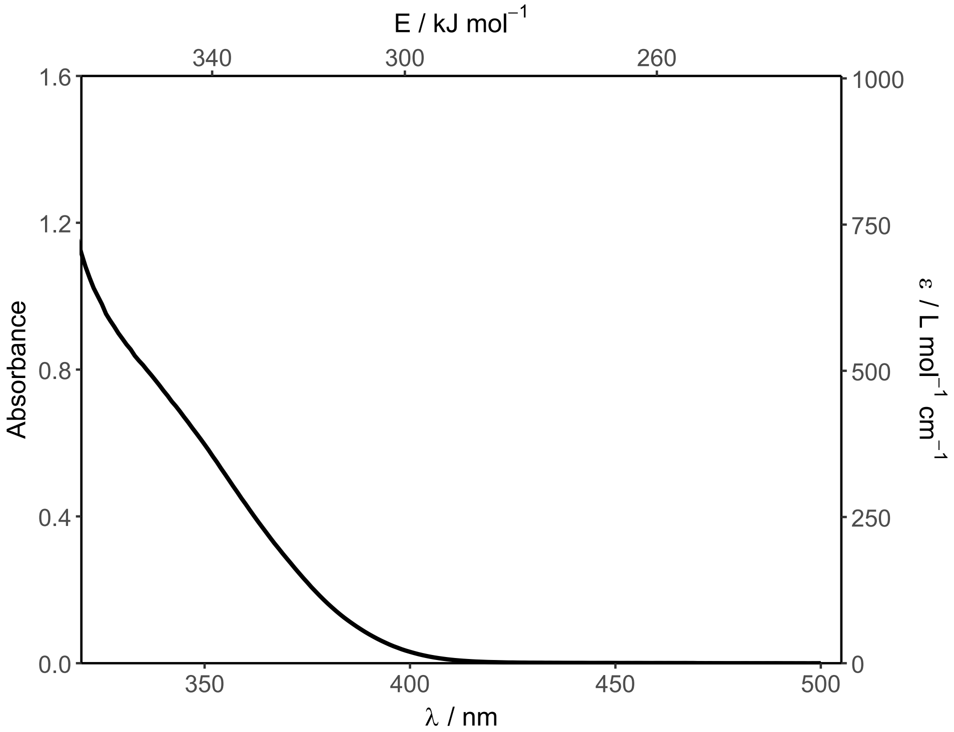

Figure S24. UV/Vis spectrum of acetate **8a** at 1.59 mmol L^{-1}.

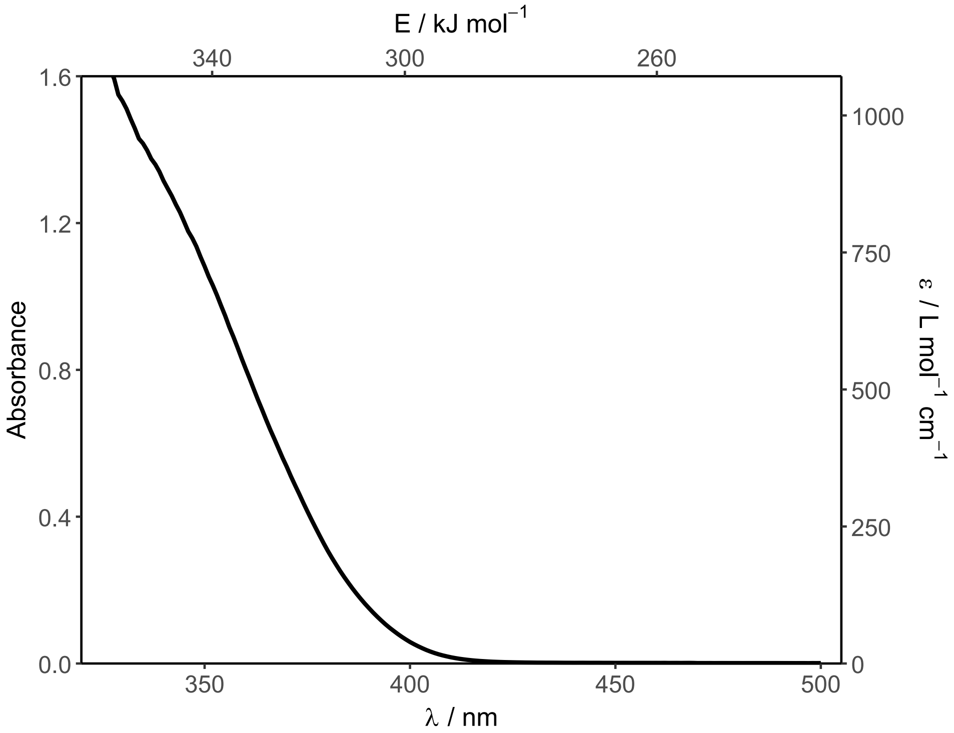

Figure S25. UV/Vis spectrum of methyl acetate **8b** at 1.49 mmol L^{-1}.

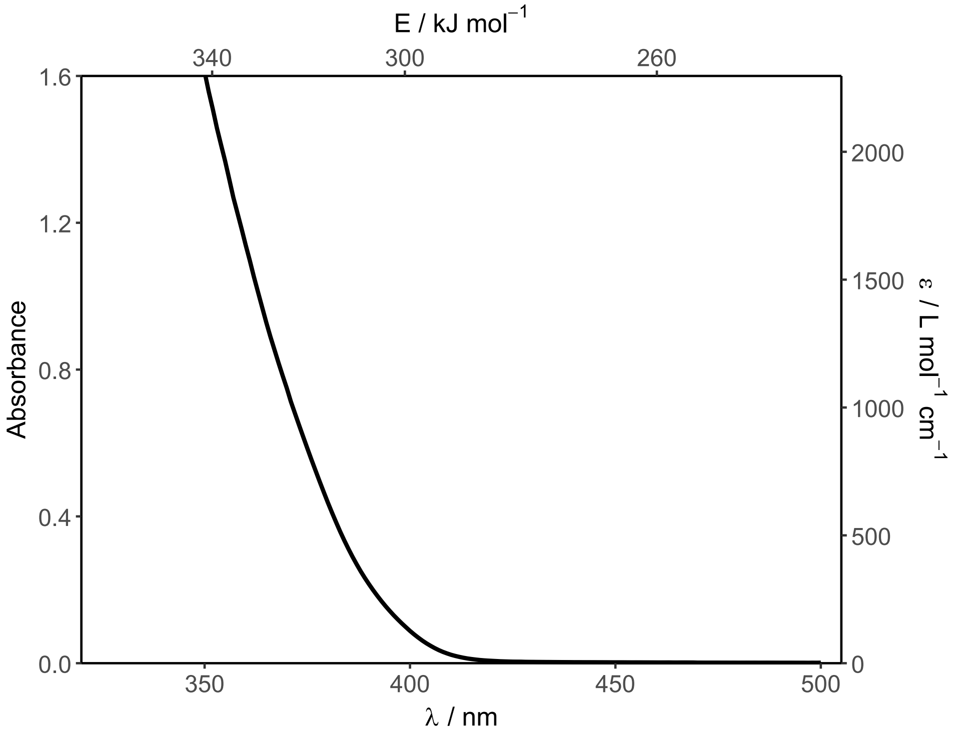

Figure S26. UV/Vis spectrum of methoxy acetate **8c** at 0.70 mmol L^{-1}.

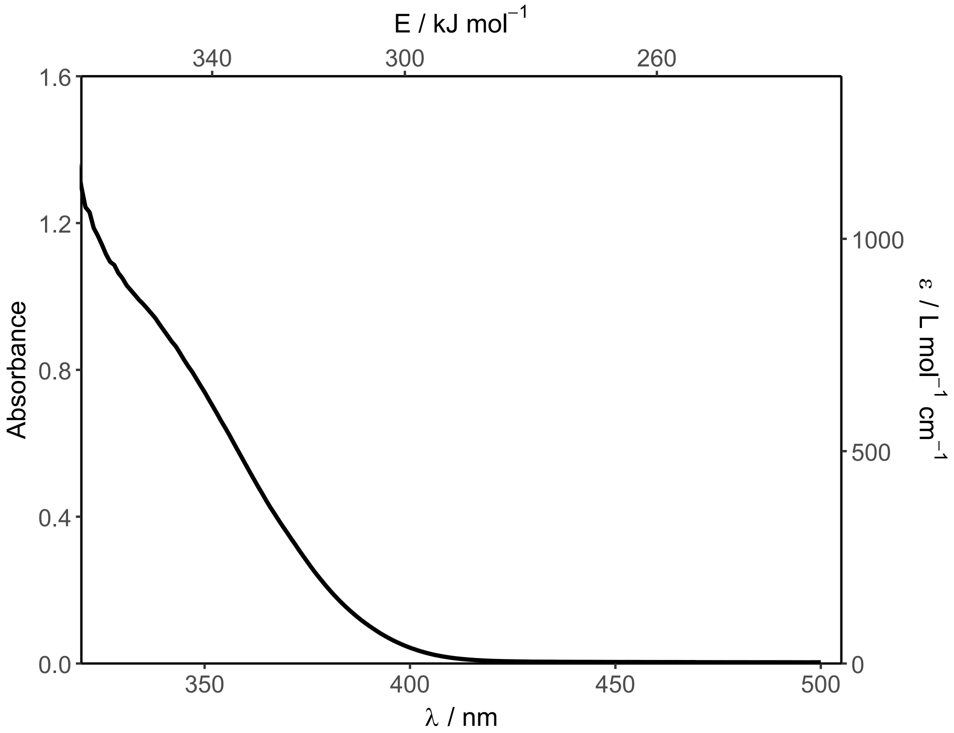

Figure S27. UV/Vis spectrum of bromo acetate **8d** at 1.16 mmol L^{-1}.

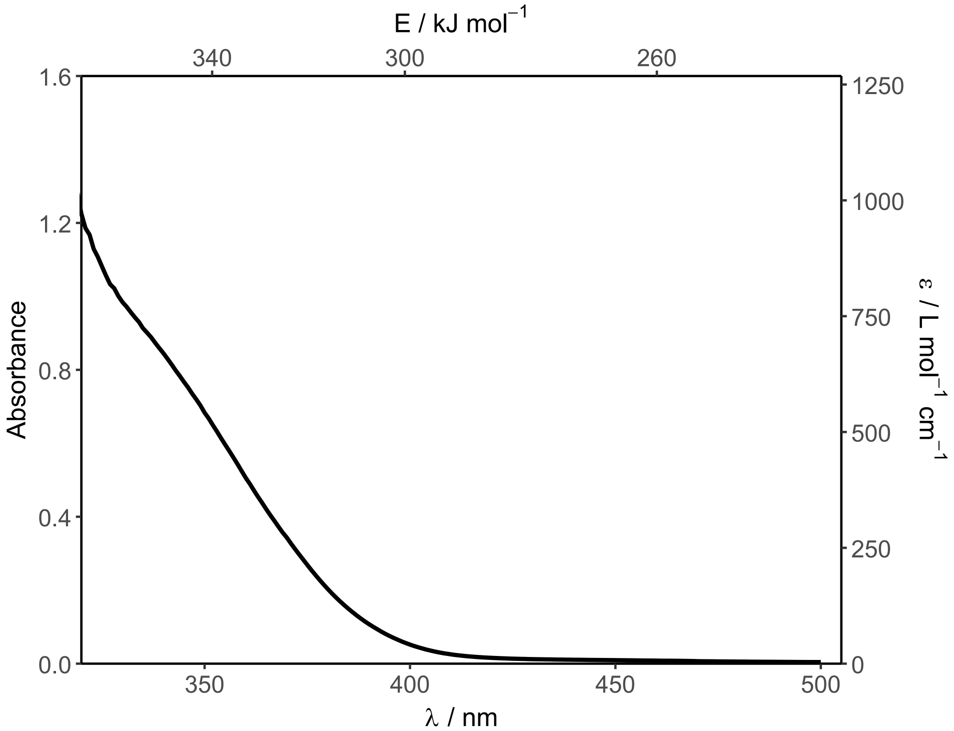

Figure S28. UV/Vis spectrum of azide acetate **8e** at 1.26 mmol L^{-1}.

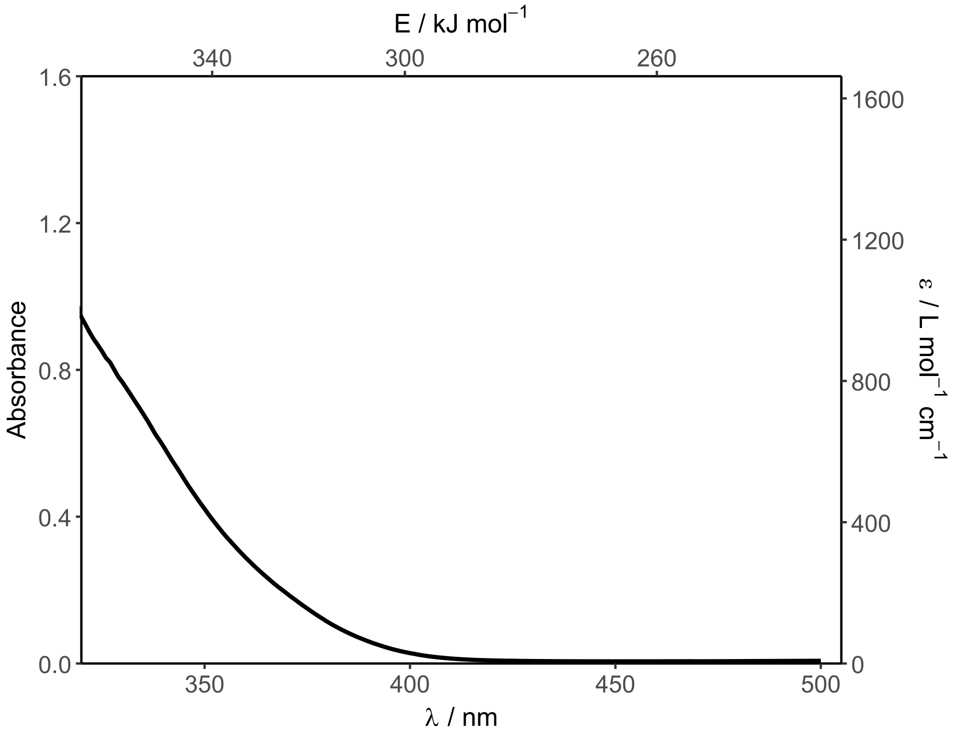

Figure S29. UV/Vis spectrum of triazole acetate **8f** at 0.96 mmol L^{-1}.

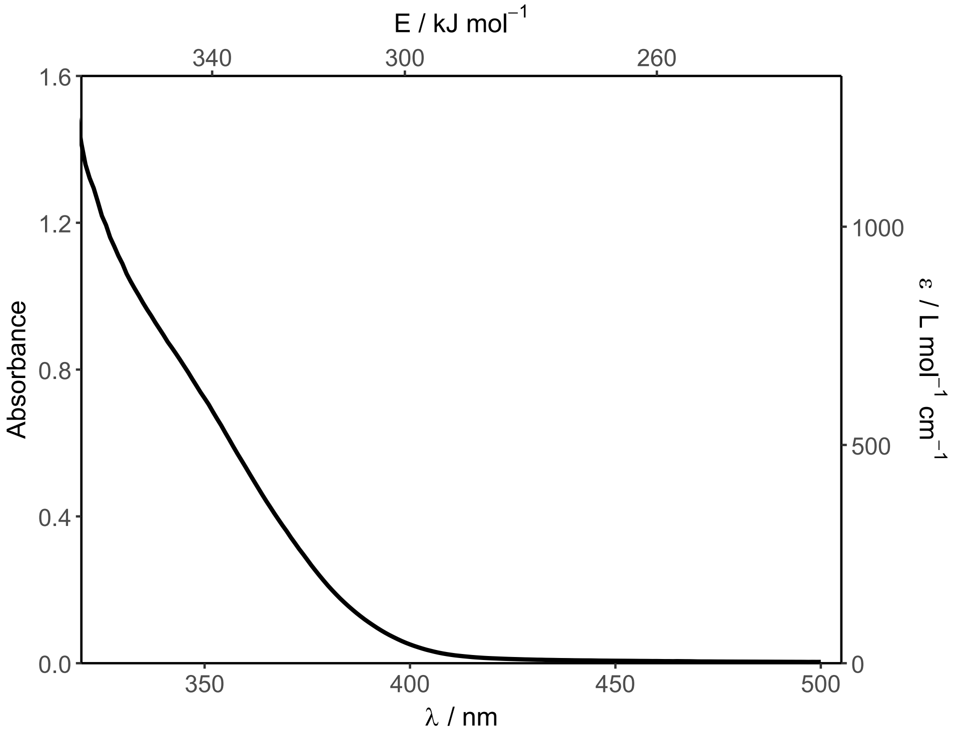

Figure S30. UV/Vis spectrum of amide acetate **8h** at 1.19 mmol L^{-1}.

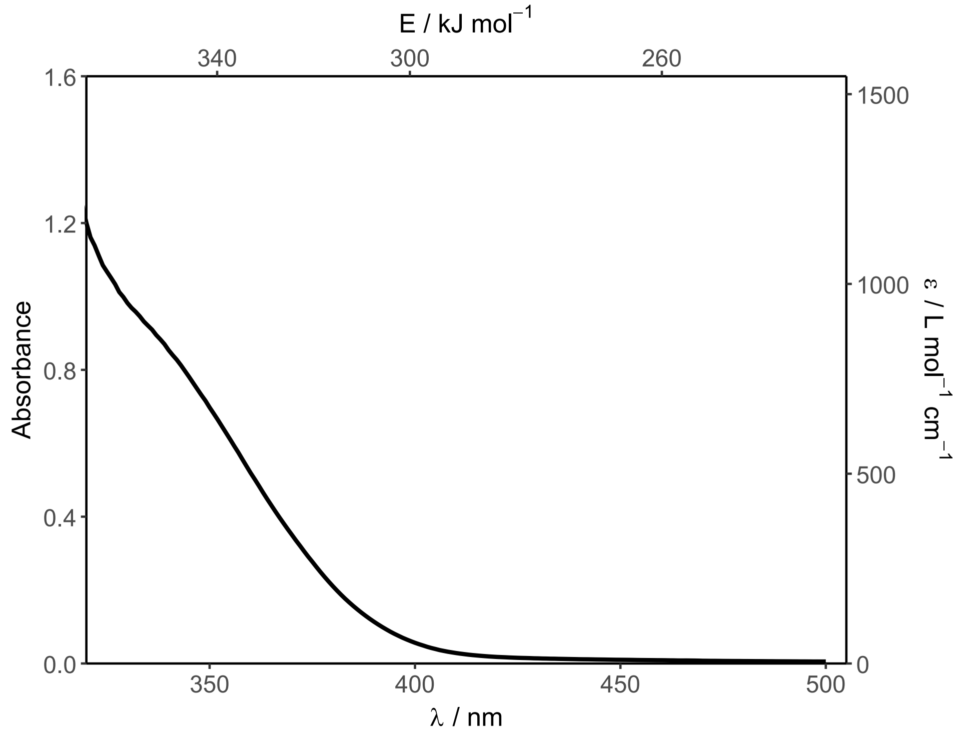

Figure S31. UV/Vis spectrum of imide acetate **8i** at 1.03 mmol L^{-1}.

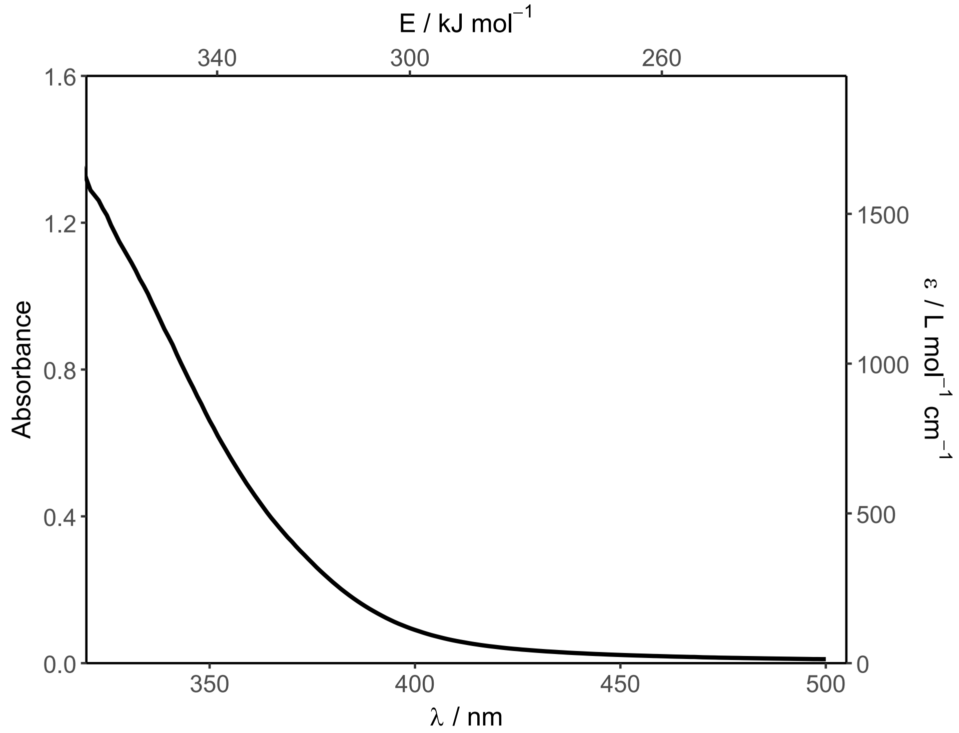

Figure S32. UV/Vis spectrum of triazole benzoate **9f** at 0.82 mmol L^{-1}.

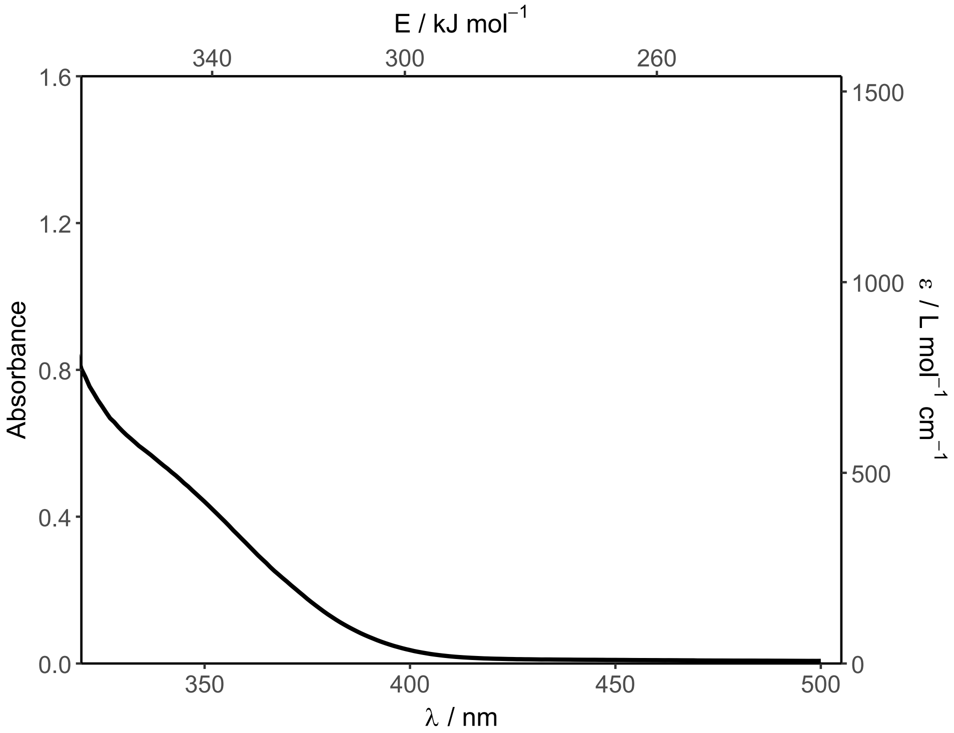

Figure S33. UV/Vis spectrum of triazole carbamate **10f** at 1.04 mmol L^{-1}.

4. Infrared spectra

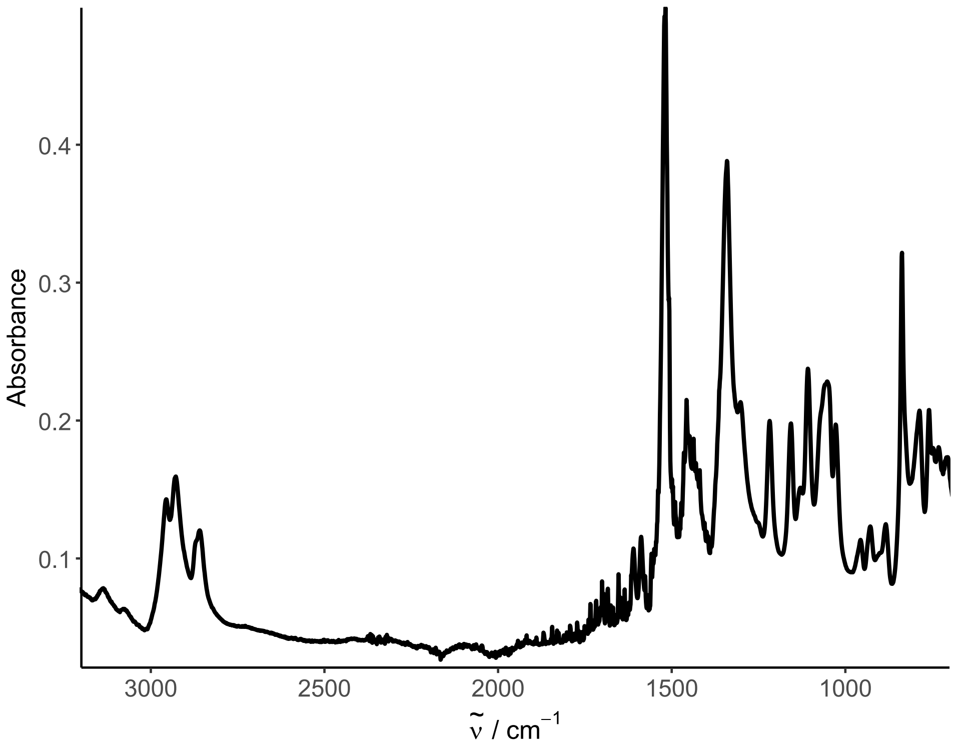

Figure S34. ATR-IR absorption spectrum of triazole alcohol **6f**.

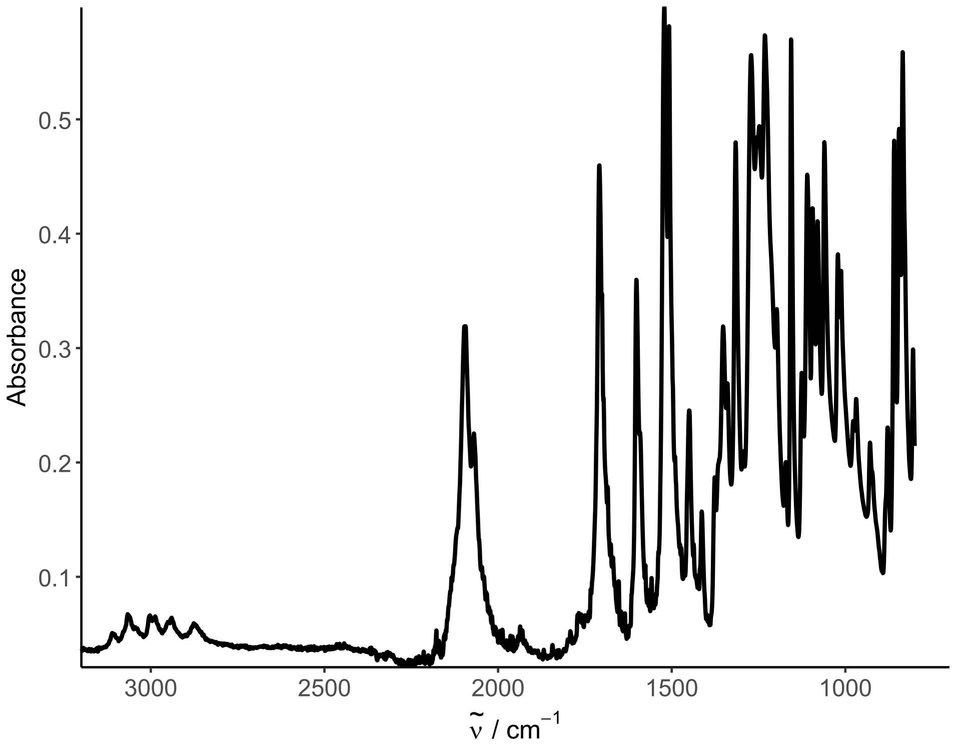

Figure S35. ATR-IR absorption spectrum of azide 4-fluorobenzoate **7e**.

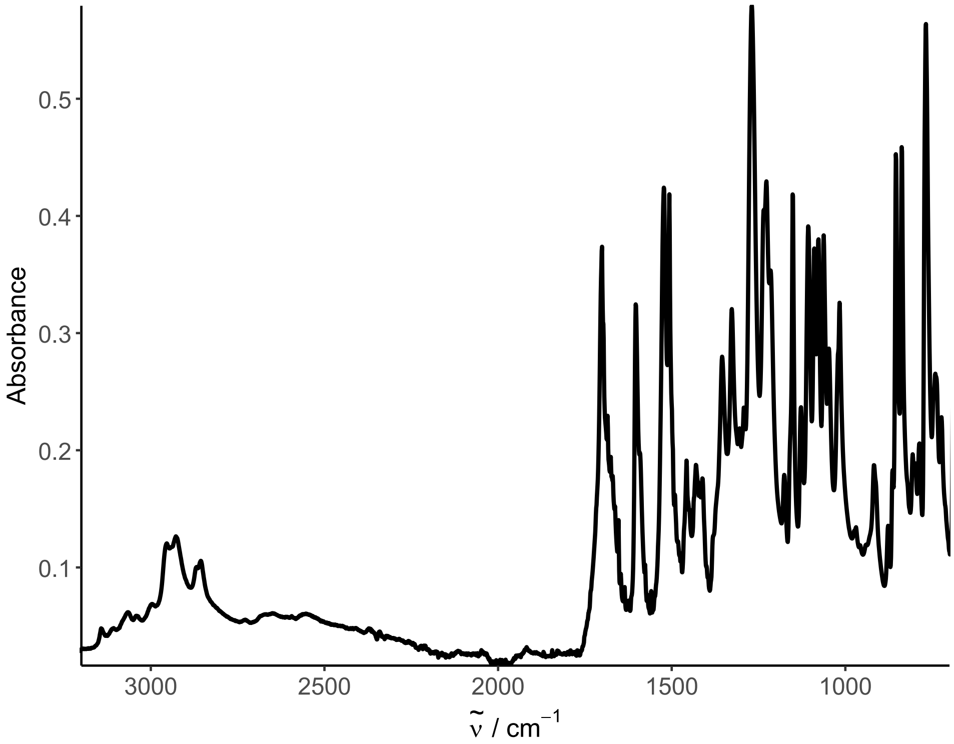

Figure S36. ATR-IR absorption spectrum of triazole 4-fluorobenzoate **7f**.

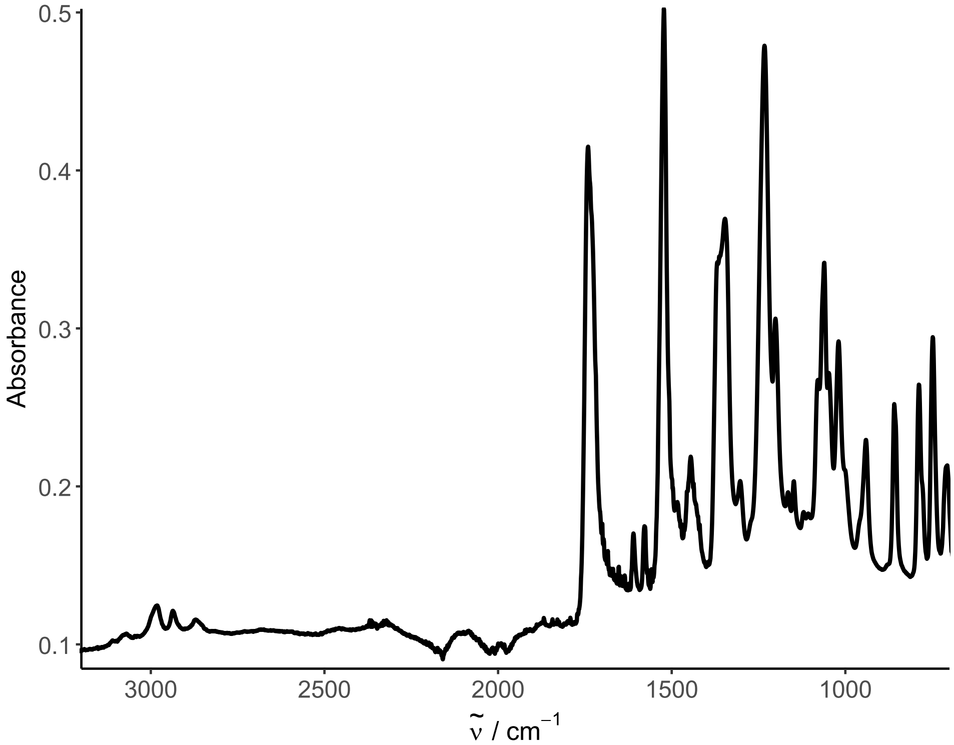

Figure S37. ATR-IR absorption spectrum of acetate **8a**.

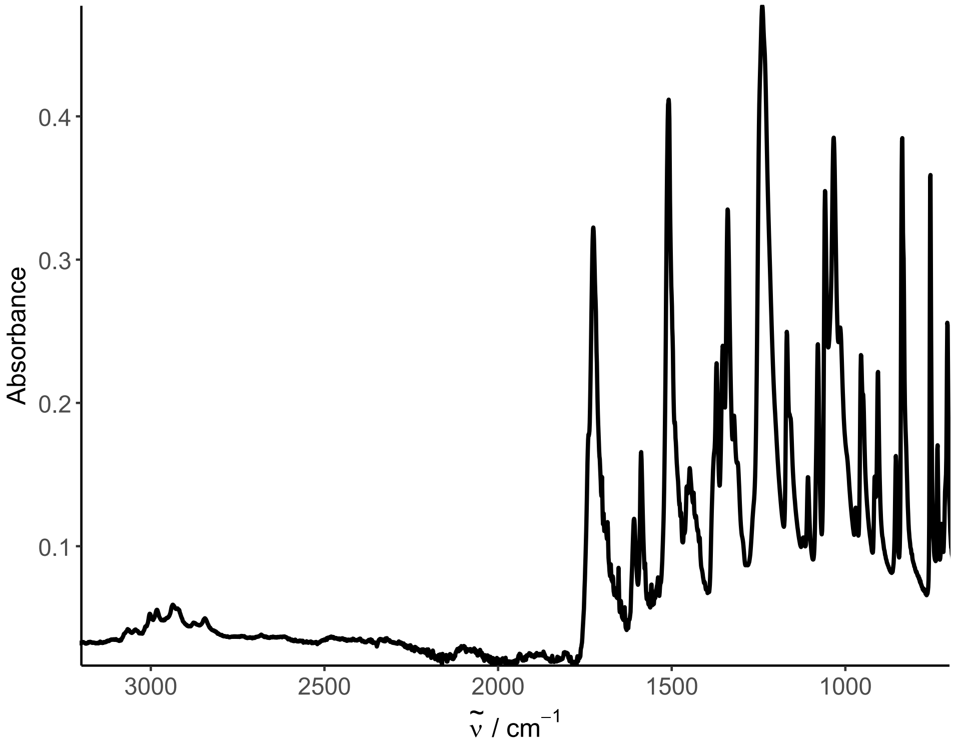

Figure S38. ATR-IR absorption spectrum of methyl acetate **8b**.

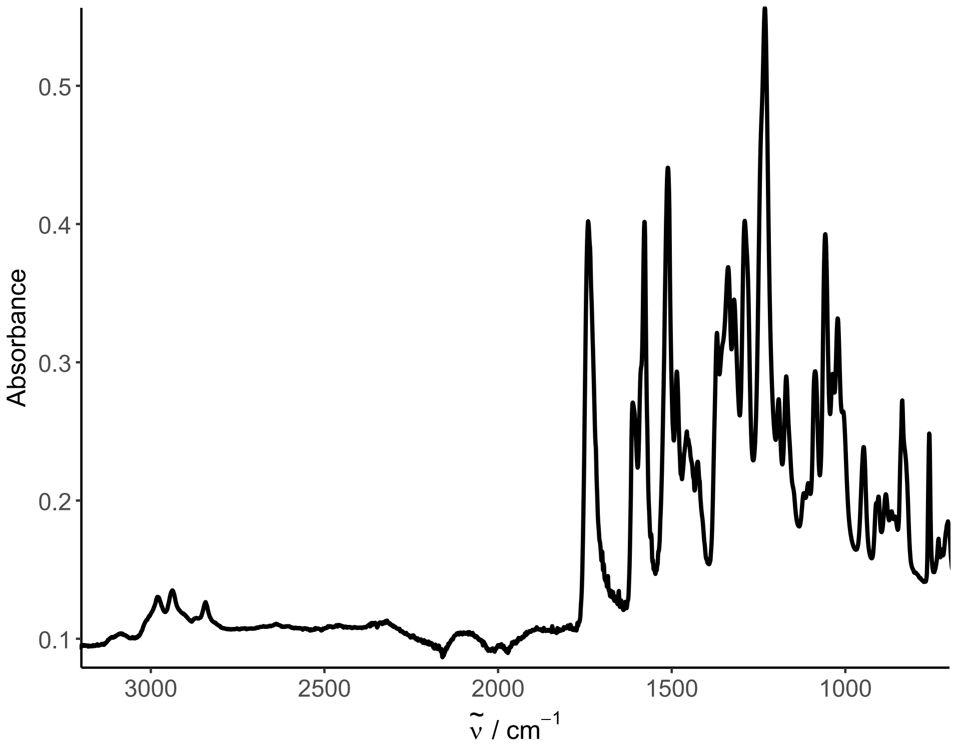

Figure S39. ATR-IR absorption spectrum of methoxy acetate **8c**.

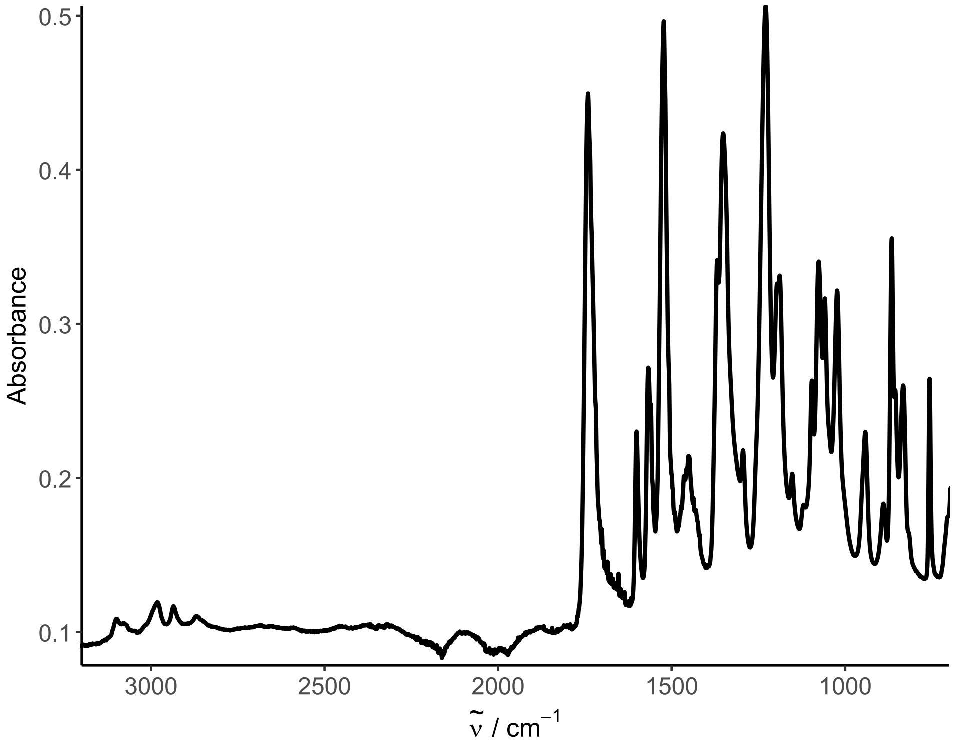

Figure S40. ATR-IR absorption spectrum of bromo acetate **8d**.

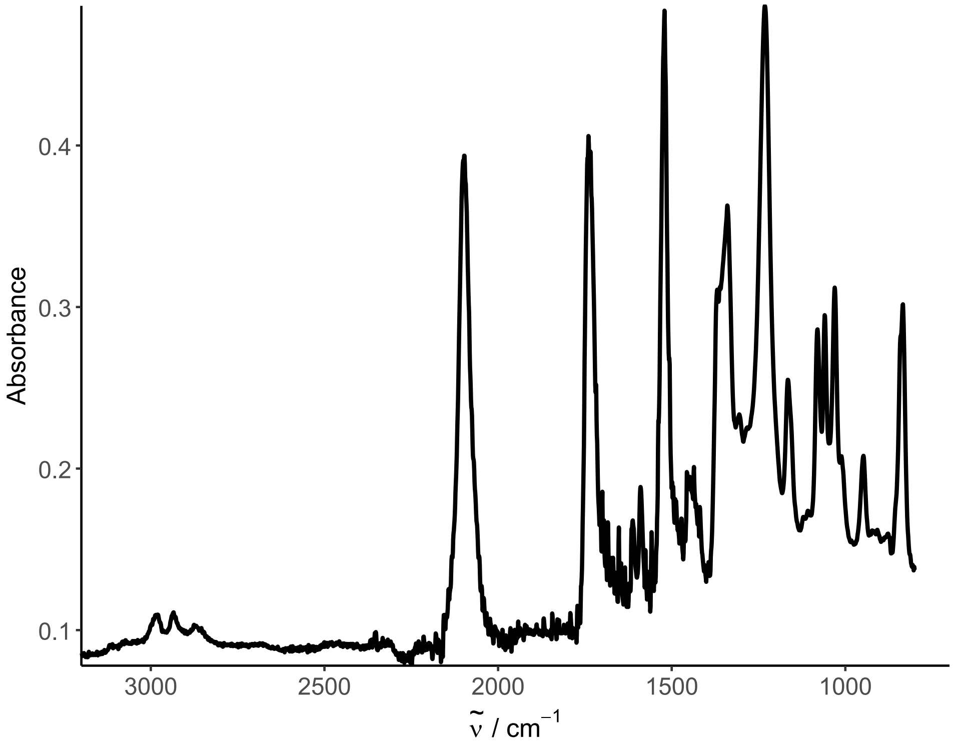

Figure S41. ATR-IR absorption spectrum of azide acetate **8e**.

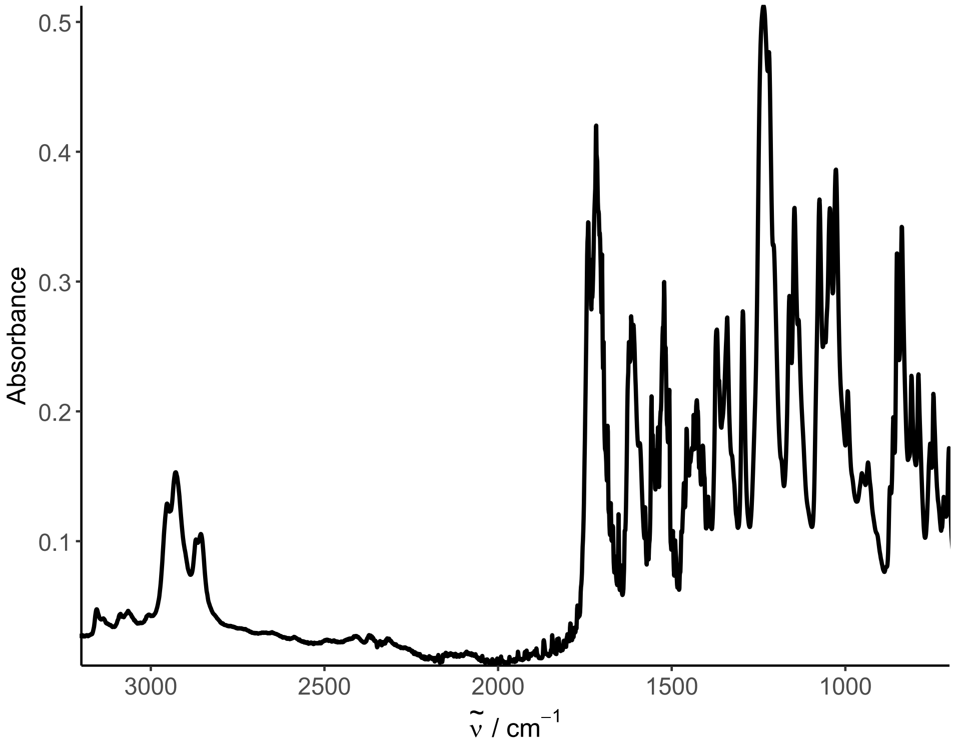

Figure S42. ATR-IR absorption spectrum of triazole acetate **8f**.

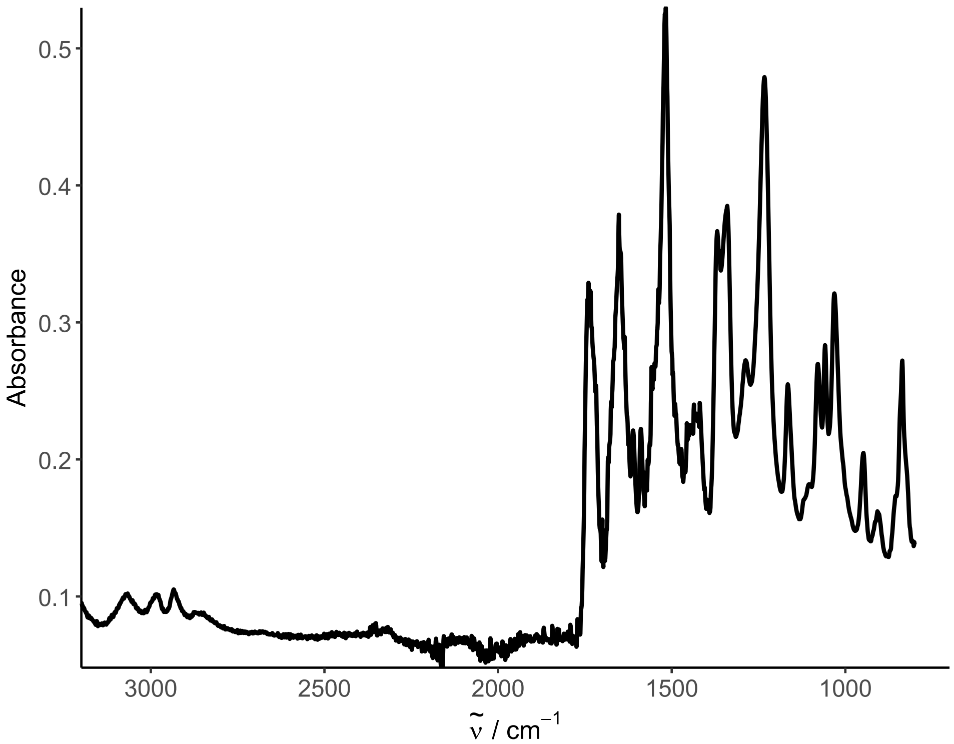

Figure S43. ATR-IR absorption spectrum of amide acetate **8h**.

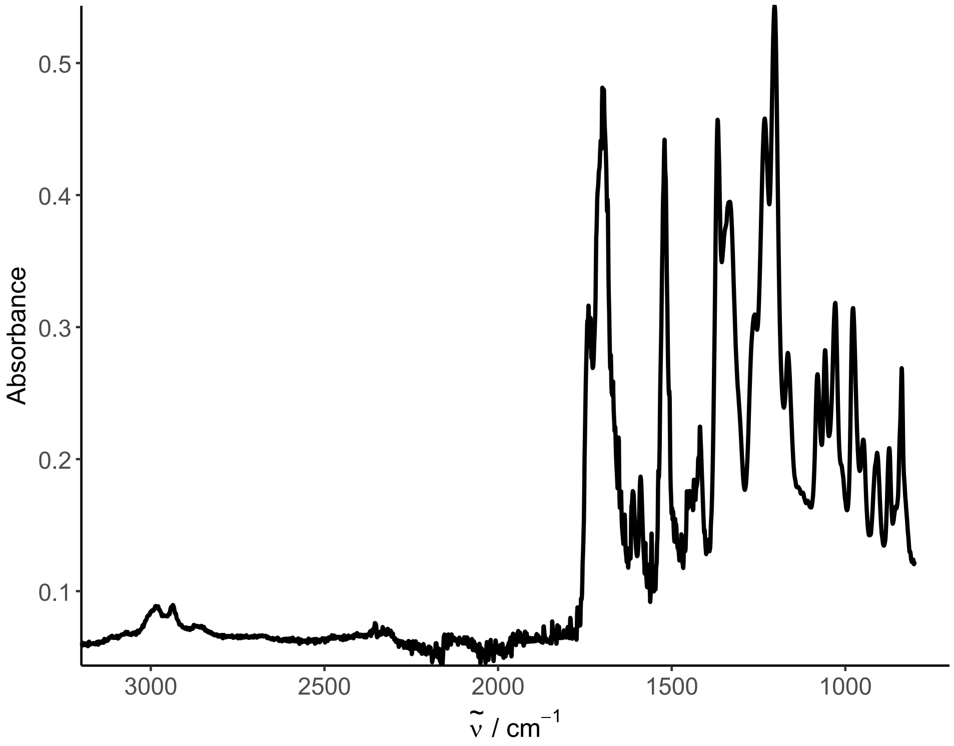

Figure S44. ATR-IR absorption spectrum of imide acetate **8i**.

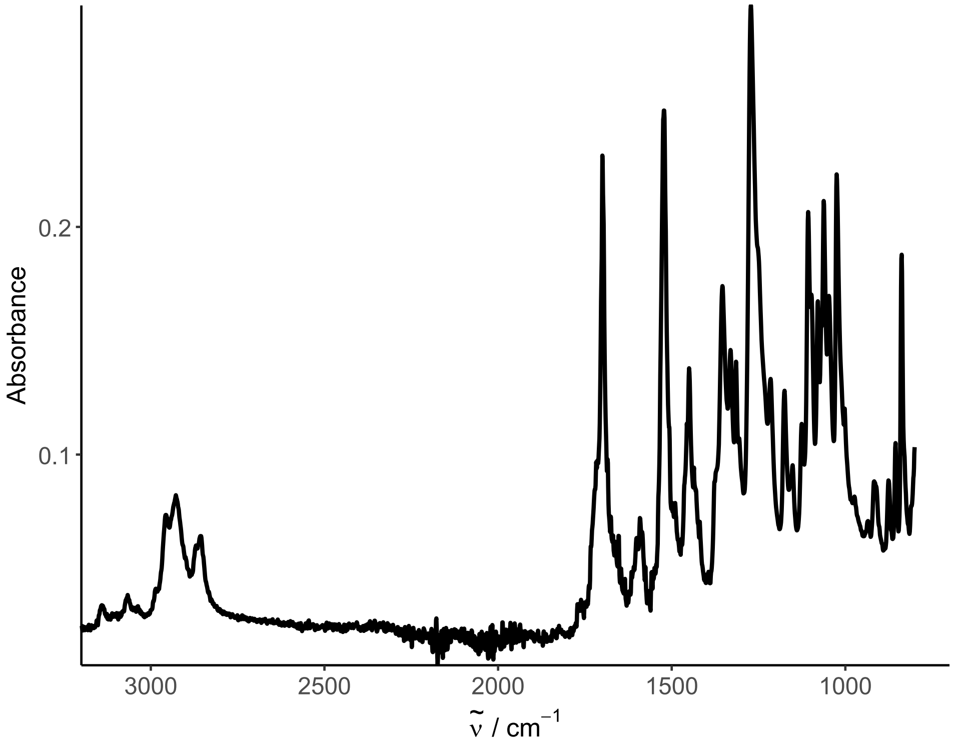

Figure S45. ATR-IR absorption spectrum of triazole benzoate **9f**.

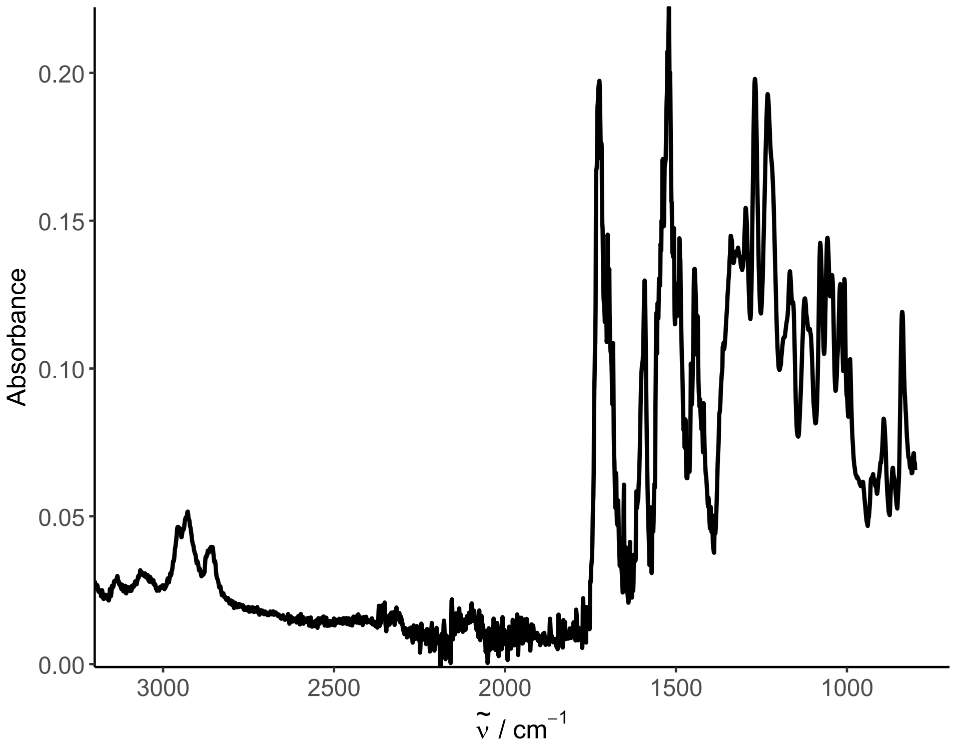

Figure S46. ATR-IR absorption spectrum of triazole carbamate **10f**.

5. Hammett parameters

As experimental Hammett parameters are not available for every substituent in this study, instead calculated Hammett parameters[4] were used. To investigate how well the calculated values σ^{calc} correlate with experimental ones[5] σ^{exp} a pairs plot was created (**Figure S47**). It reveals a very good correlation between calculated and experimental Hammett parameters, thus the use of calculated ones is justified.

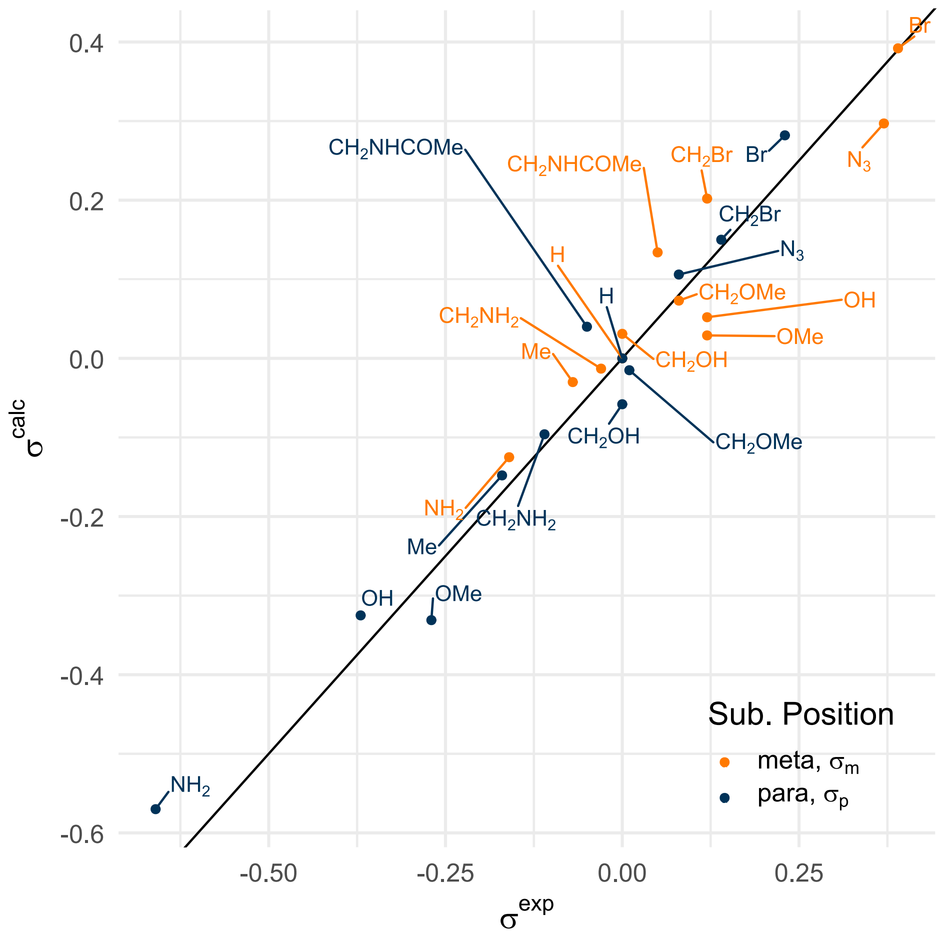

Figure S47. Pairs plot for calculated and experimental Hammett parameters.

6. References

(1) Saran, D.; Burke, D. H. A versatile photocleavable bifunctional linker for facile synthesis of substrate-DNA conjugates for the selection of nucleic acid catalysts. *Bioconjugate chemistry* **2007**, *18* (1), 275–279. DOI: 10.1021/bc060221f.

(2) Tang, L.; Yang, Y.; Wen, L.; Zhang, S.; Zha, Z.; Wang, Z. Supported gold-catalyzed and ammonia-promoted selective synthesis of quinazolines in aqueous media. *Org. Chem. Front.* **2015**, *2* (2), 114–118. DOI: 10.1039/C4QO00278D.

(3) Szychowski, J.; Mahdavi, A.; Hodas, J. J. L.; Bagert, J. D.; Ngo, J. T.; Landgraf, P.; Dieterich, D. C.; Schuman, E. M.; Tirrell, D. A. Cleavable biotin probes for labeling of biomolecules via azide-alkyne cycloaddition. *J. Am. Chem. Soc.* **2010**, *132* (51), 18351–18360. DOI: 10.1021/ja1083909. Published Online: Dec. 8, 2010.

(4) Ertl, P. A Web Tool for Calculating Substituent Descriptors Compatible with Hammett Sigma Constants**. *Chemistry Methods* **2022**, *2* (12). DOI: 10.1002/cmtd.202200041.

(5) Hansch, C.; Leo, A.; Taft, R. W. A survey of Hammett substituent constants and resonance and field parameters. *Chem. Rev.* **1991**, *91* (2), 165–195. DOI: 10.1021/cr00002a004.
